# Development of HDAC Inhibitors Exhibiting Therapeutic Potential in T-Cell Prolymphocytic Leukemia

**DOI:** 10.1021/acs.jmedchem.1c00420

**Published:** 2021-06-08

**Authors:** Krimo Toutah, Nabanita Nawar, Sanna Timonen, Helena Sorger, Yasir S. Raouf, Shazreh Bukhari, Jana von Jan, Aleksandr Ianevski, Justyna M. Gawel, Olasunkanmi O. Olaoye, Mulu Geletu, Ayah Abdeldayem, Johan Israelian, Tudor B. Radu, Abootaleb Sedighi, Muzaffar N. Bhatti, Muhammad Murtaza Hassan, Pimyupa Manaswiyoungkul, Andrew E. Shouksmith, Heidi A. Neubauer, Elvin D. de Araujo, Tero Aittokallio, Oliver H. Krämer, Richard Moriggl, Satu Mustjoki, Marco Herling, Patrick T. Gunning

**Affiliations:** † Department of Chemical and Physical Sciences, University of Toronto Mississauga, 3359 Mississauga Road, Mississauga, Ontario L5L 1C6, Canada; ‡ Department of Chemistry, University of Toronto, 80 St. George Street, Toronto, Ontario M5S 3H6, Canada; § Hematology Research Unit Helsinki, Helsinki University Hospital Comprehensive Cancer Center, Helsinki, 00029 HUS, Finland; ∥ Translational Immunology Research Program and Department of Clinical Chemistry and Hematology, University of Helsinki, Helsinki, 00014 Helsinki, Finland; ⊥ Institute for Molecular Medicine Finland (FIMM), HiLIFE, University of Helsinki, Helsinki, 00014 Helsinki, Finland; # Institute of Animal Breeding and Genetics, University of Veterinary Medicine Vienna, A-1210 Vienna, Austria; ∇ Department of Internal Medicine, Center for Integrated Oncology Aachen-Bonn-Cologne-Duesseldorf (CIO ABCD), University of Cologne (UoC), 50923 Cologne, Germany; ○ Excellence Cluster for Cellular Stress Response and Aging-Associated Diseases (CECAD), UoC, 50923 Cologne, Germany; ◆ Center for Molecular Medicine Cologne (CMMC), UoC, 50923 Cologne, Germany; ¶ Department of Cancer Genetics, Institute for Cancer Research, Oslo University Hospital, 0424 Oslo, Norway; + Oslo Centre for Biostatistics and Epidemiology, University of Oslo, 0316 Oslo, Norway; □ Centre for Medicinal Chemistry, University of Toronto Mississauga, 3359 Mississauga Road, Mississauga, Ontario L5L 1C6, Canada; △ Department of Toxicology, University Medical Center, 55131 Mainz, Germany; ● iCAN Digital Precision Cancer Medicine Flagship, 00014 Helsinki, Finland

## Abstract

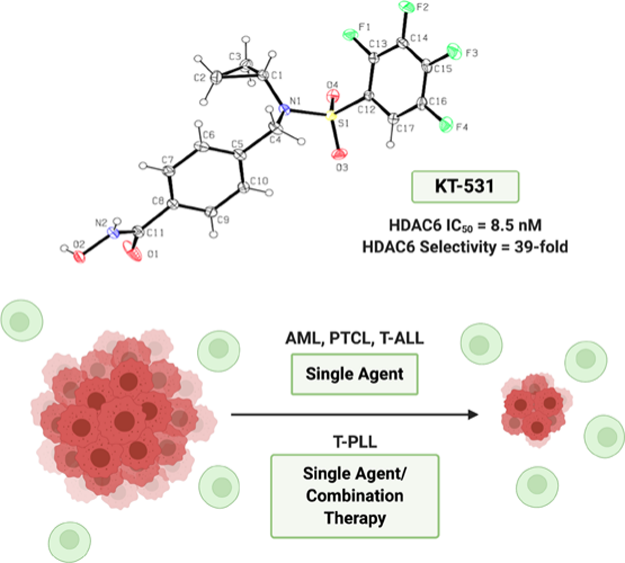

Epigenetic targeting has emerged as an efficacious therapy for hematological cancers. The rare and incurable T-cell prolymphocytic leukemia (T-PLL) is known for its aggressive clinical course. Current epigenetic agents such as histone deacetylase (HDAC) inhibitors are increasingly used for targeted therapy. Through a structure–activity relationship (SAR) study, we developed an HDAC6 inhibitor KT-531, which exhibited higher potency in T-PLL compared to other hematological cancers. KT-531 displayed strong HDAC6 inhibitory potency and selectivity, on-target biological activity, and a safe therapeutic window in nontransformed cell lines. In primary T-PLL patient cells, where *HDAC6* was found to be overexpressed, KT-531 exhibited strong biological responses, and safety in healthy donor samples. Notably, combination studies in T-PLL patient samples demonstrated KT-531 synergizes with approved cancer drugs, bendamustine, idasanutlin, and venetoclax. Our work suggests HDAC inhibition in T-PLL could afford sufficient therapeutic windows to achieve durable remission either as stand-alone or in combination with targeted drugs.

## Introduction

Epigenetic regulation of gene expression in the onset and progression of cancer has fueled therapeutic strategies against a number of molecular targets. Histone deacetylases (HDACs) have been identified as targets for reversing cancer-associated epigenetic states.^1−4^ HDACs remove acetyl groups from the amino-terminal lysine residues of histones and nonhistone proteins. This can lead to the formation of a condensed chromatin structure that consequently limits the binding of transcription factors to promoter sequences. Conversely, histone acetyltransferases (HATs) reverse this activity by catalyzing the transfer of an acetyl group from acetyl-CoA to lysine residues.^2,5^ These post-translational modifications have been identified as key regulatory strategies for a multitude of processes such as signaling networks, gene expression, transcription, cell cycle, and metabolism pathways. The activity of key regulatory proteins such as the central tumor suppressor p53, STATs, or HIF family members as well as key epigenetic regulatory proteins such as p300/CBP HATs themselves is also modulated by acetylation reactions. Hence, they also represent important targets of HDAC inhibitors (HDACi).^6−8^

The human HDAC superfamily consists of 18 nuclear and cytoplasmic proteins, distributed into 4 distinct classes according to their sequence similarity; Class I (1, 2, 3, 8), Class IIa (4, 5, 7, 9), Class IIb (6, 10), Class III (NAD^+^-dependent Sir2), and Class IV (11). Defined nonhistone substrates have been identified for certain HDAC isoforms.^3,4,7,9,10^ In particular, HDAC6 is responsible for the deacetylation of α-tubulin, HSP90, Cortactin, and Peroxiredoxin. HDAC6 is also recognized as a KDAC (lysine deacetylase). As such, HDAC6 plays a key role in microtubule dynamics and chaperone activities, DNA damage repair pathways, and oncogenic stress responses through the regulation of cell migration, immune-cell synapse formation, protein trafficking, and degradation.^11−13^ The upregulation of HDAC6 activity has been directly correlated with cellular proliferation, metastasis, and mitosis, highlighting the impact of this epigenetic regulator in cancer pathogenesis.^11,12,14^

In the past two decades, successful efforts have led to four HDACi receiving U.S. Food and Drug Administration (FDA) approval for hematological cancers: vorinostat (SAHA, **a**), romidepsin (FK-228, **b**), belinostat (PXD-101, **c**), and panobinostat (LBH-589, **d**), with several in clinical trials (quisinostat, **e**) (Figure S1a). In general, these HDACi exhibit broad spectrum HDAC activity, which limits their therapeutic tolerability owing to side effects such as fatigue, diarrhea, vomiting, anorexia, asthenia, weight loss, and thrombocytopenia.^19,20^ Selective HDACi are hypothesized to minimize side effects associated with pan-inhibitors.^2,15,16^ Since the identification of the first selective HDAC6 inhibitor tubacin (**f**) in 2003, a number of HDAC6-selective inhibitors have been developed, notably tubastatin A (**g**), ricolinostat (ACY-1215, **h**), citarinostat (ACY-241, **i**) (Figure S1a), KA2507, and marbostat-100. Ricolinostat, citarinostat, and KA2507 are currently under evaluation in advanced clinical trials for hematological and solid tumors.^17−19^ Although modest selectivity toward HDAC6 (5–6-fold) has been achieved over the nearest HDAC family member *in vitro*, the selectivity profile of these clinical candidates *in vivo* remains largely indiscriminatory toward other HDACs.^20^ Newer inhibitors such as marbostat-100 have attained higher selectivity than current clinical candidates and are well-tolerated *in vitro* and *in vivo*.^21,22^ The benign phenotype of HDAC6 knockout mice suggests that HDAC6 inhibition is a safe therapeutic strategy.^23^ Overexpression of HDAC6 and confirmed dependency profiles in multiple hematological malignancies have accelerated preclinical and clinical studies of HDAC6-selective inhibitors as single agents, and in combination with lenalidomide, pomalidomide, paclitaxel, bortezomib, or dexamethasone.^18,20,24−26^

T-cell prolymphocytic leukemia (T-PLL) is one of the most aggressive forms of hematological neoplasms.^27^ Systematic analysis, largely by sequencing efforts of T-PLL, has revealed insights into the molecular landscape of this condition, notably through recognition of recurrent *T-cell leukemia 1A* (*TCL1A*) oncogene rearrangements, damaging lesions of the *ataxia telangiectasia mutated* (*ATM*) tumor suppressor gene, and gain-of-function mutations of *Janus-activated kinase–signal transducer and activator of transcription factor* (JAK-STAT) molecules.^28−33^ These functional cancer genomic insights in T-PLL were also validated biochemically, suggesting that aberrant DNA damage and hyper-cytokine and -growth factor responses trigger neoplastic T-cell outgrowth. These responses are associated with fulminant migratory properties of T-PLL cells, and this migratory T-cell phenotype could be under HDAC6 control.^27,32,34^ Improved biological understanding has yet to translate into a therapeutic application, as patient outcomes remain poor (median overall survival <20 months), owing to the aggressive tumor growth and insufficient responses to conventional chemotherapy. The current first-line treatment for T-PLL, the monoclonal anti-CD52 antibody alemtuzumab, induces high response rates, although eventual relapses are dominant.^35^ Pan-HDACi have received FDA approval for the treatment of other mature T-cell neoplasms such as cutaneous T-cell lymphomas (CTCL) and peripheral T-cell lymphomas (PTCL) but not for T-PLL.^2,36−39^

In the absence of drugs that confer sustained tumor control in T-PLL, we focused on studying novel HDACi scaffolds for potential therapeutic use in these patients. Recent *in vitro* studies in T-PLL have also shown impressive efficacy of pan-HDACi and marked synergisms with the MDM2 inhibitor idasanutlin.^29,40^ The intertwined mechanisms of HDAC inhibition with the activity of tumor suppressor protein p53, whose repression is a hallmark in T-PLL, may be contributing to the therapeutic benefit of epigenetic modulation in this aggressive diseased state.^40−42^ Moreover, the BH3-mimetic venetoclax (ABT-199) has emerged as a breakthrough in the treatment of hematologic neoplasms. Venetoclax can induce apoptosis by targeting the B-cell lymphoma 2 (BCL-2) family of proteins. *Ex vivo* and human studies of T-PLL patients have highlighted an enhanced sensitivity to venetoclax.^41,43−45^

Here, we report the identification of a novel perfluorinated benzenesulfonamide HDAC inhibitor, KT-531 (**14**), which showed high *in vitro* HDAC6 selectivity (39-fold HDAC6 selectivity; compared to ∼6-fold selectivity of clinical candidates citarinostat and ricolinostat) with low nanomolar potency (IC_50_ = 8.5 nM) against HDAC6 in a functional *in vitro* activity assay. Furthermore, KT-531 demonstrated biological potency in multiple hematological cancer cell models (acute myeloid leukemia (AML), PTCL, and T-cell acute lymphoblastic leukemia (T-ALL)) and limited cytotoxicity in nonmalignant cell types as well as no observable toxicity *in vivo* (CD-1 mice). Notably, KT-531 exhibited strong potency (IC_50_ = 0.42 μM) in the T-ALL/T-PLL-like cell line SUP-T11. Data mining using Oncomine revealed *HDAC6* was the only HDAC member to be selectively overexpressed in T-PLL patient samples but not in other mature or immature T-cell malignancies, supporting the rationale for HDAC6-targeting in treatment of T-PLL.^46^ KT-531 was subsequently tested in ten primary T-PLL patient samples, where it exhibited promising drug sensitivity scores (DSS), with a therapeutic window for T-PLL over healthy donor-derived peripheral blood mononuclear cells (PBMCs). Finally, KT-531 showed high synergy with chemotherapeutic agents idasanutlin, bendamustine, and venetoclax in T-PLL patient samples, representing a novel, efficacious, and potentially safer combination for T-PLL treatment. To the best of our knowledge, KT-531 is the first HDAC6 inhibitor to show efficacy in T-PLL patient samples.

## Results

### Chemistry

**Figure 1:**
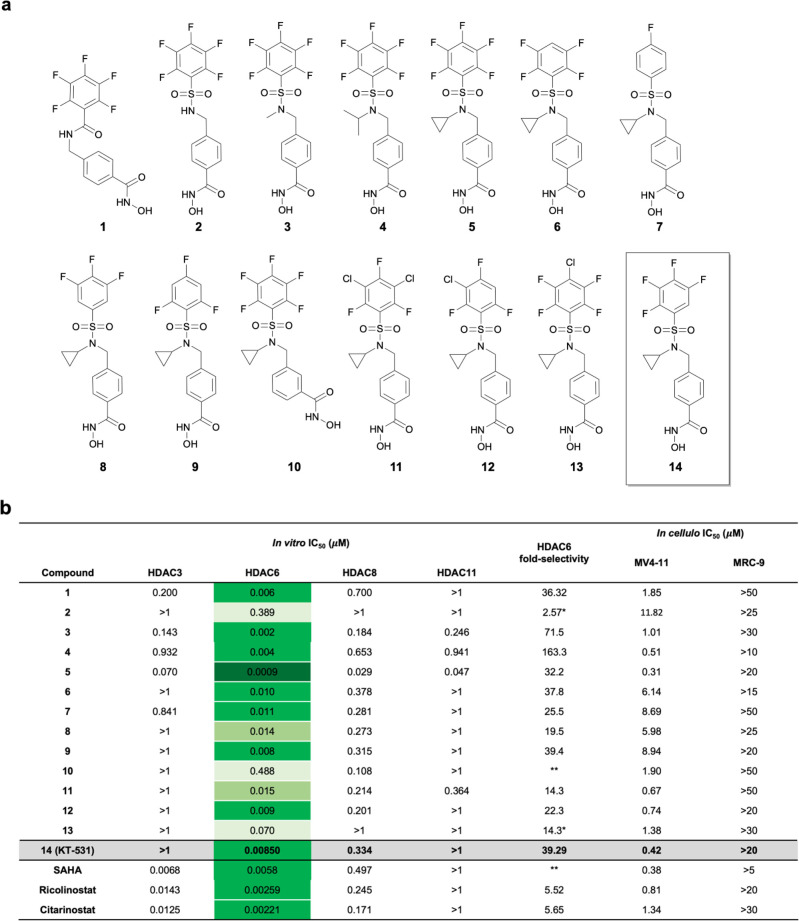
HDACi **1**−**14** and their *in vitro* activity and selectivity profiles. (a) Chemical structures of HDACi **1**–**14**. (b) Biochemical HDAC inhibition against recombinant enzymes (Nanosyn) and cellular cytotoxicity results in MV4-11 (AML) and MRC-9 (healthy fibroblasts). Note: **not HDAC6 selective, *selectivity ≥ indicated value (beyond limit of experiment). A color gradient is applied for visualization of differential HDAC6 activity.

Herein, we describe a new class of perfluorinated benzenesulfonamide HDAC6-selective inhibitors, which originated from our recently reported HDAC6-selective inhibitor, JG-265 (**1**).^47^ A comprehensive structure–activity relationship (SAR) study of **1** was designed to identify inhibitors that retained/amplified HDAC6 potency and selectivity, while improving the limited pharmacokinetic (PK) profile which precluded its advancement to preclinical studies (Figure 1a,b).^47^

**Scheme 1:**
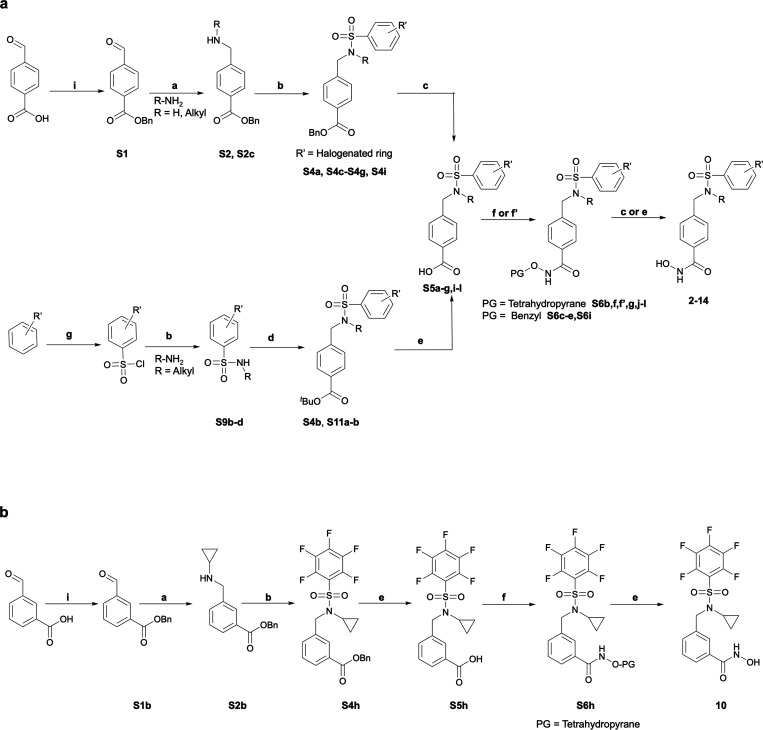
Synthetic Conditions for Preparation of **2**–**14** (a) Reagents and conditions to synthesize **2**–**9** and **11**–**14**. (i) BnBr, Cs_2_CO_3_, DMF, RT, 24 h; (a) (i) R–NH_2_, AcOH, DCE, RT, 2 h; (ii) NaBH(OAc)_3_, RT, 16 h; (b) R′SO_2_Cl, Et_3_N, CH_2_Cl_2_, 3–16 h, RT; (c) H_2_, 10% Pd/C, THF/MeOH (2:1), RT, 16 h; (g) HSO_3_Cl, 3 h, 150°C; (b) RNH_2_, Et_3_N, CH_2_Cl_2_, RT, 3–16 h **d)** C_12_H_15_BrO_2_, Cs_2_CO_3_, DMF, RT, 24 h; **e)** 4M HCl/dioxane, 0°C-RT, 3 h; (f) (i) (COCl)_2_, THF, DMF, 0°C, 1 h; (ii) H_2_N-OTHP, ^i^Pr_2_NEt, THF, RT, 16 h; (f′) H_2_N-OBn, EDCI, HOBt, Et_3_N, DMF, RT, 16–24 h; (c) H_2_, 10% Pd/C, THF/MeOH (2:1), RT, 16–24 h; (e) 4M HCl/dioxane, 0°C-RT, 3 h. **b** Reagents and conditions to synthesize **10. (i)** BnBr, Cs_2_CO_3_, DMF, RT, 24 h; **a)** (i) ^c^Pr-NH_2_, AcOH, DCE, RT, 2 h; (ii) NaBH(OAc)_3_, RT, 16 h; (b) PFBSCl, Et_3_N, CH_2_Cl_2_, 3–16 h, RT; (e) H_2_, 10% Pd/C, THF/MeOH (2:1), RT, 18 h; (f) (i) (COCl)_2_, THF, DMF, 0°C, 1 h; (ii) H_2_N-OTHP, ^i^Pr_2_NEt, THF, RT, 16 h; (e) H_2_, 10% Pd/C, THF/MeOH (2:1), RT, 6 h.

Synthetic pathways used to prepare the final compounds (Figure 1a) are outlined in Scheme 1. In order to synthesize a subset of the desired inhibitor library using Scheme 1a (upper branch), 4-formyl benzoic acid was protected using benzyl bromide to form benzyl 4-formylbenzoate (**S1**) (70%), which was reductively aminated with the appropriate amines to form the desired secondary amine (**S2**, **S2c**) (83%). The corresponding amine was coupled to polyhalogenated benzenesulfonyl chloride (**S3**) via an amine sulfonylation reaction to yield the sulfonamide precursors (**S4a**, **S4c–S4g**, **S4i**) (61–91%). If the precursor polyhalogenated benzenesulfonyl chloride (**S3**) was commercially unavailable, it was synthesized via the sulfonylation of the corresponding polyhalogenated benzene with chlorosulfonic acid at 150 °C for 2 h. After removal of the benzylic ester protecting group (**S5a**, **S5c**–**S5g**, **S 5f’**, **S5i**, **S5l**) (71–98%) from the sulfonamide precursors, it was coupled to tetrahydropyranyl (THP) or benzyl-protected hydroxylamines (**S6a**, **S6c**–**S6g**, **S6f’**, **S6i**, **S6l**) (61–76%). A final deprotection using *H*_2_(*g*), Pd/C, or 4 M HCl (35–81%) yielded the final molecules (**2**, **3**, **5**, **7**, **9**, **11**–**13**).

The alternate strategy (lower branch of Scheme 1a) was used to design compounds **4**, **6**, and **14**. Polyfluorobenzenesulfonyl chloride (prepared as previously described if not commercially available) was coupled to the respective amines to generate the desired sulfonamides (**S9b**–**d**) (68–83%), which were subsequently coupled to 4-bromomethylbenzoic *tert*-butyl ester to form the corresponding *tert*-butyl ester-protected benzoic acids (**S4b**, **S11a**,**b**) (27–82%). Following acid-mediated deprotection (**S5b**,**j**,**k**) (99%), coupling to O-(Tetrahydro-2H-pyran-2-yl)hydroxylamine yielded the protected hydroxamic acid (**S6b**,**j**,**k**) (88–98%) which was readily deprotected to reveal the final compounds **4** (50%), **6** (49%), and **14** (60%). Compound **1** was generated as previously reported.^47^

A similar synthetic route (Scheme 1b) was utilized to furnish 3-(((*N*-cyclopropyl-2,3,4,5,6-pentafluorophenyl)sulfonamido)methyl)-*N*-hydroxybenzamide, by beginning with the benzyl ester protection of 3-carboxybenzaldehyde (**S1b**) (98%) and reductively aminating with cyclopropylamine to yield benzyl 3-((cyclopropylamino)methyl)benzoate (**S2b**) (93%). Coupling to pentafluorobenzenesulfonyl chloride (**S4h**) (78%) and following the aforementioned deprotection yielded 3-(((*N*-cyclopropyl-2,3,4,5,6-pentafluorophenyl)sulfonamido)methyl)benzoic acid (**S5h**) (98%). The synthesis was completed through conversion to the hydroxamate ester (**S6h**) (70%) and deprotecting with acid to furnish compound **10** (66.7%).

### Structure–Activity Relationships, *In Vitro* HDAC Inhibition, and *In Cellulo* Cytotoxicity

The prepared library (Figure 1a) was screened against HDAC3, 6, 8, 11 (representative of groups I, II, and IV) to determine *in vitro* activity inhibition profiles (Figure 1b).^48,49^ Concurrently, cellular activity was analyzed in a model cancer cell line (MV4-11) and healthy fibroblasts (MRC-9) to correlate biochemical inhibition with cellular potency and therapeutic window, and other HDACi (SAHA/vorinostat, ricolinostat, and citarinostat), as well as parent compound **1**, were included for parallel comparison.

First, the role of the pentafluorobenzamide moiety was investigated by substituting it with a pentafluorobenzenesulfonamide (PFBS), **2**. This resulted in a substantial loss of activity against HDAC6 (IC_50_[HDAC6] = 389 nM) vs **1** (IC_50_[HDAC6] = 5.9 nM) and a subsequent loss in cellular potency (IC_50_[MV4-11] = 11.07 μM) vs **1** (IC_50_[MV4-11] = 1.85 μM). The introduction of an acidic sulfonamide group likely incurs unfavorable electrostatic interactions with tunnel residues. This hypothesis is supported by the *N*-methylated analogue, **3**, being ∼200 fold more potent. Moreover, substitutions on the *N*-sulfonamide with alkyl groups of different sizes (−CH_3_, **3**; −^i^Pr, **4**; −^c^Pr, **5**) showed improved HDAC6 potency (∼200-fold for **3**, ∼100-fold for **4**, and ∼430-fold for **5**) and cellular IC_50_’s (0.31–1.04 μM). One of the most notable improvements included the selectivity jump from 36-fold for the parent compound **1** to 163-fold for **4** (Figure 1b). Furthermore, **5**, with an *N*-^c^Pr substituent, exhibited picomolar inhibitory activity (IC_50_ = 0.9 nM) toward HDAC6.

The *N*-^c^Pr group of **5** was retained, and the importance of the halogenation and electrophilicity was explored next. Diminishing cellular cytotoxicity in MV4-11 cells was observed by successively removing of fluorine atoms from **5**. Although mono-, tri-, and tetrafluorobenzenesulfonamides (**6**–**9**) retained biochemical HDAC6 inhibition (IC_50_ = 8–14 nM), cellular efficacy was reduced by ∼5–10-fold (IC_50_ = 5–9 μM), suggesting that substitution of the phenyl sulfonamide ring is important for biological activity. HDAC selectivity profiles ranged from 20-fold for **8** to 163-fold for **4**. Selectivity toward HDAC isoforms was modulated by varying the position of the hydroxamic acid, specifically, *para-* to *meta-*, which switched selectivity toward HDAC8 (4-fold) (**10**).

Although covalent modification of HDAC6 was not observed (Figure S2), the PFBS group is susceptible to nucleophilic aromatic substitution by biological nucleophiles. A previously described high-throughput *in vitro* glutathione-reactivity assay was conducted, where compounds were incubated in the presence of glutathione (GSH) and the formation of a GSH-compound adduct was monitored over time via LC/MS/MS.^50^ Fluorinated analogues **3** and **4** were found to react readily with glutathione, which precluded further advancement (Table S1a). The exceptional picomolar HDAC6 potency of **5** and moderate GSH stability (half-life of 20 min) warranted further ADME analysis. In hepatocytes, **5** was cleared rapidly, with a half-life of 3 min (Table S1b). In contrast, **6**, lacking the *para* F, was unreactive toward GSH (Table S1a). However, in mouse plasma, **6** had a low half-life of 25 min (Table S2a).

**Figure 2:**
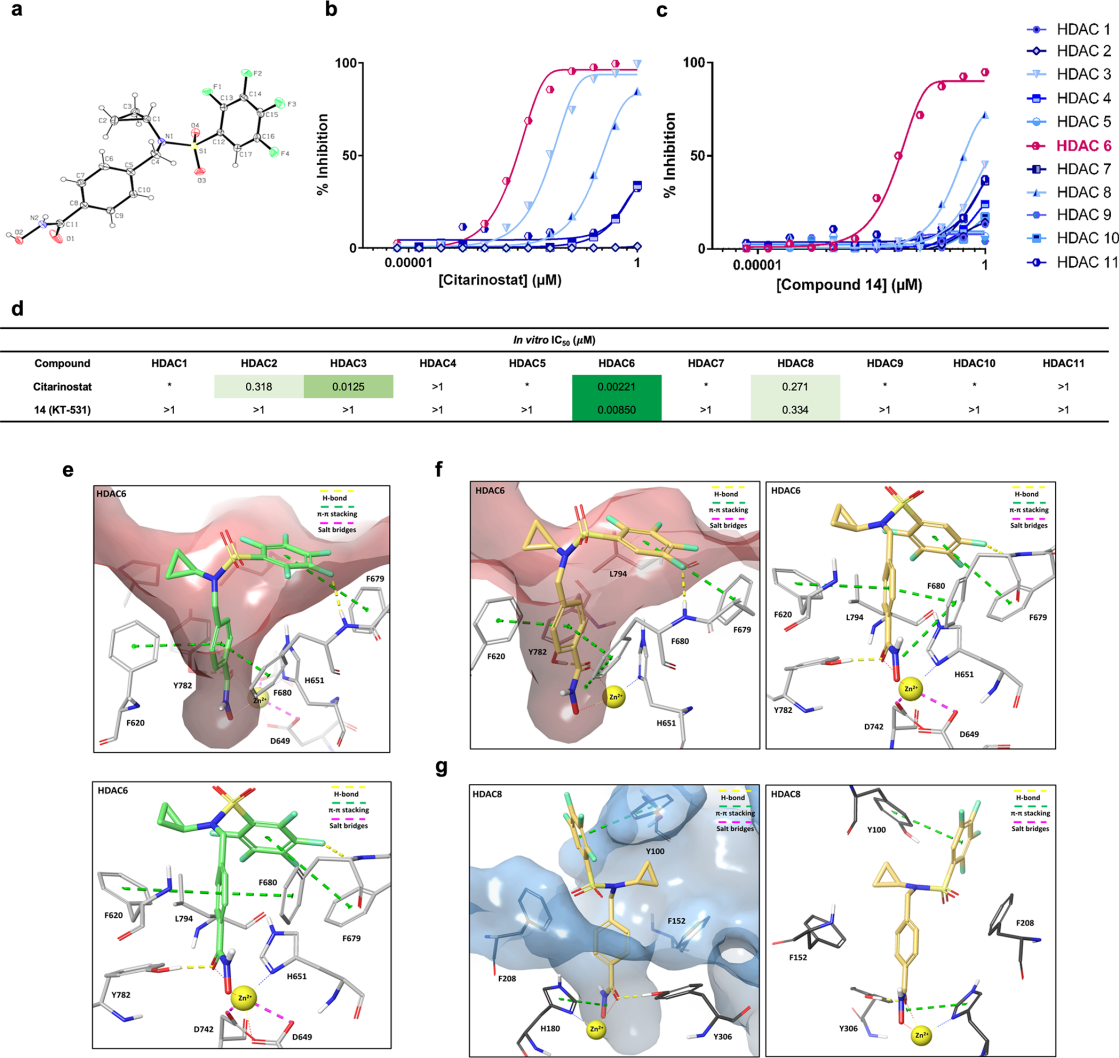
*In vitro* and *in silico* confirmation of superior HDAC6-selectivity and potency of compound **14** (KT-531). (a) X-ray crystal structure of **14**. (b) Dose–response curve representing the percent inhibition and HDAC6 selectivity of citarinostat against HDAC2–4,6,8,11 via EMSA activity assay (Nanosyn). (c) Dose–response curve representing the percent inhibition and HDAC6-selectivity of **14** against HDAC1–11. (d) *In vitro* HDAC inhibition IC_50_ values of citarinostat (i) and **14** against HDAC1–11. A color gradient is applied for visualization of differential activity. (e) *In silico* docking binding pose of **5** in human HDAC6 (PDB 5EDU), with Zn^2+^ (yellow sphere), hydrogen bonds (yellow dashed line), π–π stacking (green dashed line), and salt bridges (purple dashed line) (N, blue; O, red; C, green; F, blue-green). Catalytic triad residues and portions of the protein have been omitted for clarity. (f) *In silico* docking binding pose of **14** in human HDAC6 (PDB 5EDU), with Zn^2+^ (yellow sphere), hydrogen bonds (yellow dashed line), π–π stacking (green dashed line), and salt bridges (purple dashed line) (N, blue; O, red; C, yellow; F, green). Catalytic triad residues and portions of the protein have been omitted for clarity. (g) *In silico* docking binding pose of **14** in human HDAC8 (PDB 1T64).

We next investigated strategies to improve stability by reducing the electrophilicity of the perfluorinated cap group. The phenyl group was substituted with a variety of halogen combinations (**11**–**14**). A homologue of **5**, missing the 4-fluoro substituent, **13**, showed significantly reduced potency toward HDAC6 (∼700-fold). The loss of HDAC6 inhibition was concomitant with reduced biological activity in the cytotoxicity experiments. Analogues where fluorine substituents were replaced with less electronegative chlorine atoms exhibited comparable HDAC6 inhibition and cellular activity, but reduced selectivity. Inhibitor **14** (KT-531) (Figure 2a), with a tetrafluorobenzene substituent led to a substantial increase in stability. While no discernible reactivity with GSH was observed after 24 h (Table S1a), KT-531 showed cellular activity (IC_50_ [MV4-11] = 0.42 μM), single digit nanomolar potency for HDAC6 (IC_50_ = 8.5 nM), and a promising selectivity profile (39-fold over the next closest target, HDAC8) (Figure 1b). KT-531 exhibited a superior *in vitro* profile when compared to the current literature HDAC6 inhibitor, Nexturastat, which had comparable HDAC6 potency (IC_50_ = 12.4 nM) but a lower selectivity margin (19-fold; next nearest target HDAC3 with IC_50_ = 238 nM) (Table S2b).^51,52^ KT-531 also demonstrated higher cytotoxicity than Nexturastat in MV4-11 cancer cells (IC_50-Nexturastat_ = 1.68 μM, IC_50-KT-531_ = 0.42 μM) (Table S2b, Figure 1b).

The binding kinetics profile of KT-531 was also investigated by determining the association (*k*_on_)/dissociation (*k*_off_) rates as well as residence time parameters. The *in vitro* residence time (1/*k*_obs_) for the KT-531–HDAC6 complex was determined to be 23.18 min, as compared to 10.72 min for **1**. This is a significant improvement over **5**, which exhibited a residence time of 1.09 min (Table S3). Reversible ligand binding was confirmed for **5** and **14** with unchanged IC_50_ values in the 6 h time-dependent assay (Figure S2). The *K*_i_ of KT-531 for HDAC6 was determined to be 17.3 nM in a time-dependent inhibition *K*_on_ experiment (Figure S3).

The selectivity profile of KT-531 for all HDAC families (Class I, II, and IV) was determined using an activity-based EMSA assay (Nanosyn, USA). KT-531 showed a clean selectivity profile, with an IC_50_ of >1000 nM for HDAC1–5, 7, and 9–11 and modest potency against HDAC8 (IC_50_ = 334 nM). This ∼40-fold selectivity was higher than that of HDAC6-selective clinical candidates, ricolinostat and citarinostat (both 5–6-fold selective, Figures 1b and 2b–d). Unlike KT-531, both clinical candidates inhibit Class I HDACs, specifically HDAC3 (IC_50_ of citarinostat = 12.5 nM).

### *In Silico* Modeling and Docking Studies

Docking simulations between *hs*HDAC6 (PDB 5EDU, catalytic domain 2) and **5** using Schrödinger’s Maestro v11.9 (Schrödinger, LLC, New York, 2019) were performed to understand the role of the PFBS ring in deriving potency toward HDAC6 (Figures 2e and S4).^53−55^ The core interactions in the catalytic tunnel included sandwich-type π–π interactions of **5** with Phe620 and Phe680, coupled with an additional T-shaped π–π stacking of the PFBS ring with Phe679 (Figure 2e). Hydrogen bonding interactions between the fluorine substituents and the nearby backbone of Phe679 likely contributes to the increase in overall affinity. The flexible sulfonyl center and the benzylic −CH_2_– kink atom serve to accommodate these interactions (Δ*G*_B_ = −8.84 kcal mol^–1^). The fluorine atoms were deemed crucial for efficacy and HDAC6 pocket-occupancy, as replacement of the *para* fluorine to a chlorine atom led to a significant decline in potency (∼77-fold loss in potency from 0.9 to 70 nM). Through docking simulations of **13**, loss of activity was predicted to be due to the bulkier chlorine substituent not being well accommodated in the HDAC6 catalytic tunnel outlet (Δ*G*_B_ = −6.67 kcal mol^–1^) (Figure S5).

Similar analyses of **14** (KT-531), showed the same key interactions with Phe620 and Phe680 in the HDAC6 pocket (Δ*G*_B_ = −8.30 kcal mol^–1^), and hydrogen bonding with the nearby Phe679 amide, despite the loss of F (Figure 2f). When docked in the HDAC8 catalytic pocket, KT-531 lost the tunnel π–π stacking interactions, and only retained a T-shaped stacking interaction with the proximal Tyr100 (Δ*G*_B_ = −5.70 kcal mol^–1^) (Figure 2g). The fluorinated ring was unable to effectively bind to the opening of a catalytic tunnel characterized by an intractable, sterically hindered topology. Although π–F interactions are also likely, Maestro does not have the functionality to distinguish such interactions. Overall, these findings provide a structural rationale for the high potency of KT-531 toward HDAC6 (IC_50_ = 8.5 nM), and the corresponding lower affinity toward HDAC8 (IC_50_ = 334 nM).

### *In Cellulo* Pharmacology

**Figure 3:**
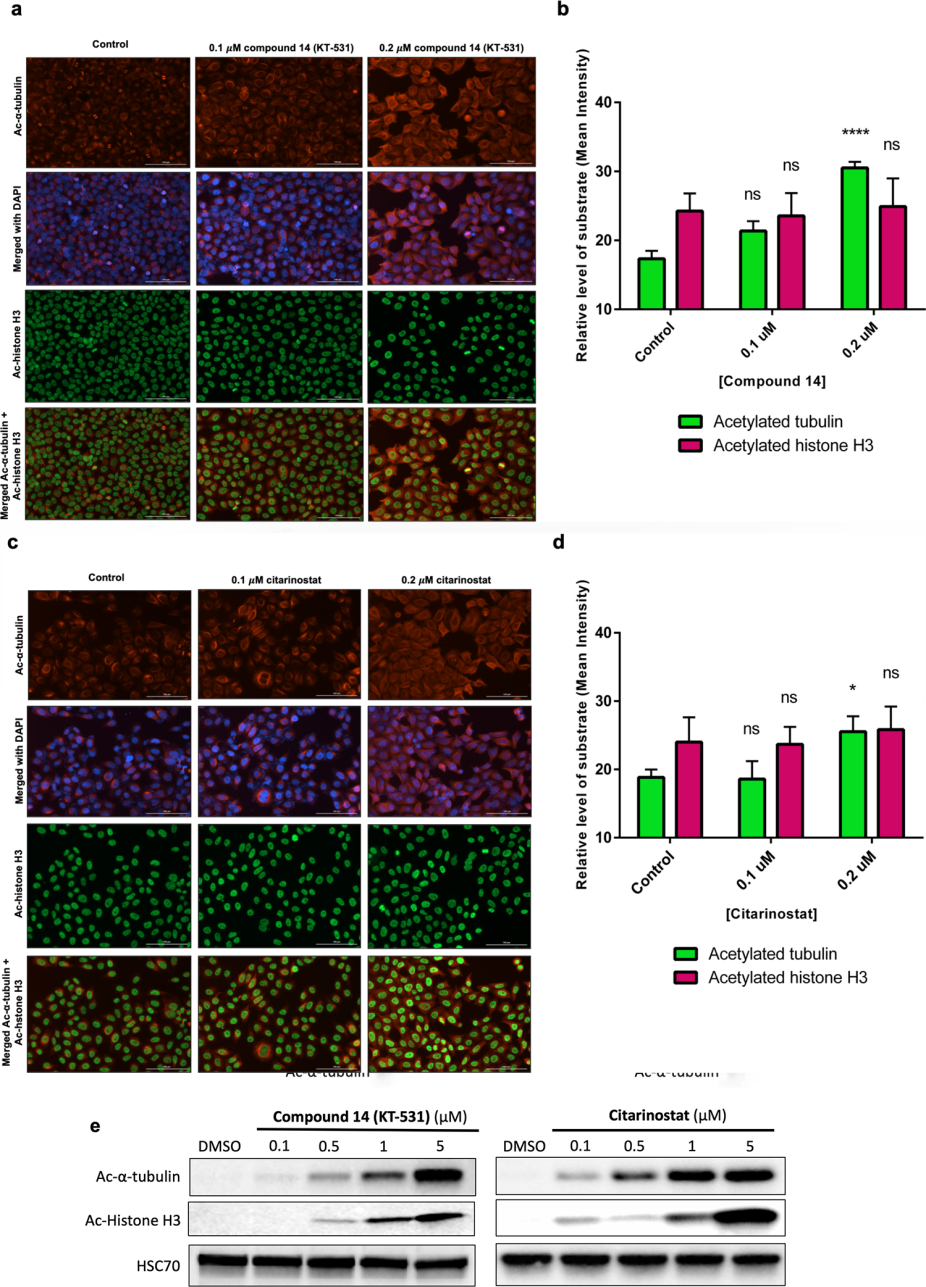
*In cellulo* pharmacology of compound **14** (KT-531) in comparison to citarinostat. (a) Immunofluorescence analysis of α-tubulin acetylation and histone H3 acetylation following 6 h treatment of KT-531 in cells. (acetylated α-tubulin in red, acetylated histone H3 in green, and nuclear stain DAPI in blue). This data is representative of three independent experiments. (b) Quantification of fluorescent signals that correspond to levels of acetylated α-tubulin and acetylated histone H3 in cells dosed with KT-531 at indicated concentrations. (c) Immunofluorescence analysis of α-tubulin acetylation and histone H3 acetylation following 6 h treatment of citarinostat in cells (acetylated α-tubulin in red, acetylated histone H3 in green, and nuclear stain DAPI in blue). This data is representative of three independent experiments. (d) Quantification of fluorescent signals that correspond to levels of acetylated α-tubulin and acetylated histone H3 in cells dosed with citarinostat at indicated concentrations. *p ≤ 0.05; ****p ≤ 0.001; ns, nonsignificant; two-way ANOVA with Tukey’s multiple comparisons test. (e) Western blot illustrating α-tubulin acetylation and histone H3 acetylation levels in MV4-11 AML cells following 6 h treatment with varying concentrations of KT-531 and clinical candidate citarinostat (i). Protein extracts were prepared, resolved by SDS-PAGE and immunoblotted with acetylated α-tubulin, acetylated histone H3, and HSC70 antibodies. A representative Western blot of three independent experiments is shown.

KT-531 was examined for its ability to induce acetylation of α-tubulin, a prominent substrate of HDAC6, and histone H3 (HDAC Class I substrate).^56^ Initially, model cancer cells (HeLa) were subjected to 6 h incubation with increasing doses of KT-531 or citarinostat and target engagement was analyzed via immunofluorescence assays. In KT-531-treated cells, significant acetylation of α-tubulin was observed, with no observable change in acetylation of histone H3 (Figure 3a,b). Citarinostat induced weaker acetylation as compared to KT-531 (Figure 3c,d).

Next, KT-531-treatment of a hematopoietic cancer cell line, MV4-11, revealed that, at higher inhibitor concentrations, acetylated α-tubulin levels were comparable between citarinostat and KT-531 (Figures 2d and 3e). Off-target acetylated histone H3 levels were higher with citarinostat in comparison to KT-531, aligning with *in vitro* findings (Figures 2d and 3e).

**Figure 4:**
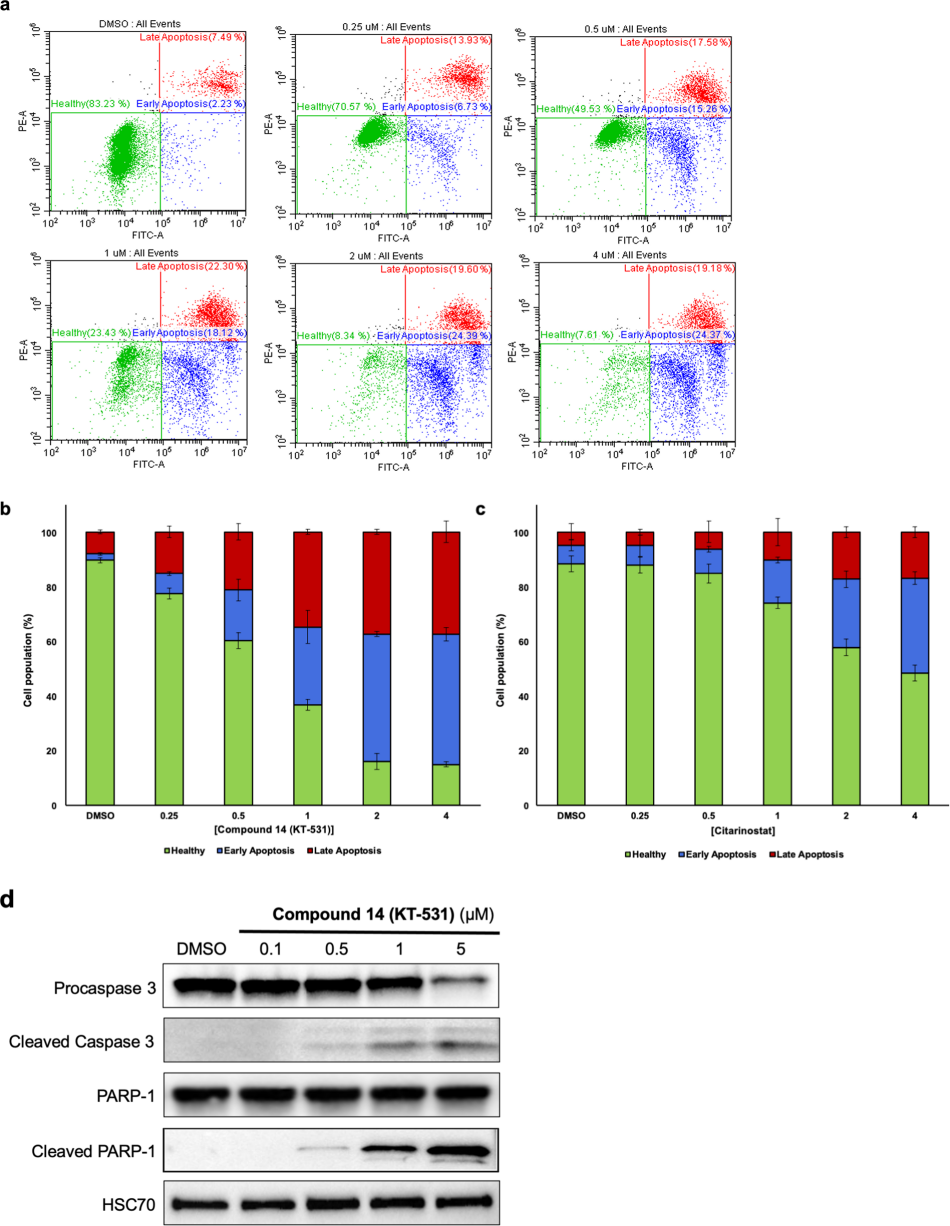
Compound **14** (KT-531) induces apoptosis in a dose-dependent manner. (a) Flow cytometric data of MV4-11 cells treated with increasing concentrations of KT-531 (0–4 μM) for 18 h and analyzed for apoptosis via Annexin V/PI staining. Representative dot blots from three independent experiments are presented. (b) Percentage of cell populations in healthy, early apoptosis, and late apoptosis phases following treatment with KT-531 for 18 h at indicated concentrations. (c) Percentage of cell populations in healthy, early apoptosis, and late apoptosis phases following treatment with citarinostat for 18 h at indicated concentrations. Flow cytometric data for citarinostat is available in Figure S6. (d) Cleavage of caspase-3 and PARP-1 upon dose-escalation of KT-531 for 6 h in MV4-11 cells. Protein extracts were prepared and subjected to SDS-PAGE and immunoblotting with procaspase 3, cleaved caspase-3, PARP-1, cleaved PARP-1, and HSC70 antibodies. A representative Western blot of three independent experiments is shown. Data for citarinostat is available in Figure S7.

To understand the origin of the cytotoxic activity, fluorescence-activated cell sorting (FACS) was employed to evaluate KT-531 (Figure 4a,b). Annexin V/PI staining revealed 15% of cells underwent early apoptosis (Annexin V+/PI) upon 18 h treatment with 0.5 μM KT-531, in contrast to 9% for citarinostat at the same dose (Figures 4c and S6). At 2 μM KT-531, only 8% of cells remained healthy and nonapoptotic, whereas 47% of cells remained as nonapoptotic following 2 μM treatment with citarinostat. Similar trends were observed for late apoptotic populations (Annexin V+/PI+) (22% for KT-531 versus 10% for citarinostat at a 1 μM dose). Cleavage of PARP-1 and Caspase-3 at 0.5 μM treatment of KT-531 confirmed the apoptotic response signature (Figure 4d). The absence of cleaved Caspase-3 and PARP-1 at higher concentrations of citarinostat (5 μM) recapitulated FACS findings that KT-531 (**14**) induces apoptosis more strongly than the clinical molecule (Figure S7).

### Therapeutic Potential of KT-531 in T-Cell Neoplasms

**Figure 5:**
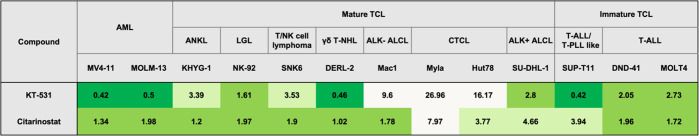
KT-531 shows selective cellular cytotoxicity in PTCL malignancies. Heat map of IC_50_ values calculated from drug response analysis of KT-531 (**14**) and citarinostat from one representative out of three independent experiments. Cell lines are classified according to the respective disease subtype. Abbreviations: AML, acute myeloid leukemia; ANKL, aggressive NK-leukemia; LGL, large granular lymphocyte leukemia; T-NHL, T-cell non-Hodgkin’s lymphoma; ALK^+^ ALCL, anaplastic large cell lymphoma (anaplastic lymphoma kinase positive); CTCL, cutaneous T-cell lymphoma; T-ALL, T-cell acute lymphoblastic leukemia.

Similarities in the mutational landscape of myeloproliferative and lymphoid neoplasms suggest that similar treatments might be effective.^57,58^ Since biological activity was demonstrated in AML, KT-531 was further assessed in a collection of PTCL (*n* = 8) and T-ALL (*n* = 3) model cell lines covering various subtypes to understand if efficacy was transferable (Figure 5).

KT-531 displayed selective cytotoxicity in different PTCL cell lines. A striking observation of this screen in PTCL and T-ALL models was the indiscriminate behavior of citarinostat toward all branches of these malignancies, in contrast to the more selective cellular behavior of KT-531 in certain subtypes. Unlike citarinostat and FDA-approved pan-HDACi romidepsin and vorinostat, KT-531 was not very active in CTCL models, such as Myla and Hut78 (Figure 5).^40,43,59^ Another distinction of KT-531 from citarinostat was its strong activity in the T-ALL/T-PLL-like cell line SUP-T11 (IC_50_ of 0.42 μM; 9-fold more potent than citarinostat). SUP-T11 (derived from a patient with mature T-ALL) harbors a TRA/TRD-TCL1A translocation and consequent aberrant expression of the TCL1A protein, which is considered the genetic hallmark of T-PLL. SUP-T11 is, hence, the closest T-PLL-model derived from a patient so far.^57,59,60^ These findings also correlate with the HDAC6 mRNA expression levels available in Broad Institute Cancer Cell Line Encyclopedia (CCLE; Figure S8). The poor activity of KT-531 in Hut78 cells is consistent with the relatively low HDAC6 expression. The highest sensitivity to KT-531 was observed for T-ALL cell models SUP-T11, DND-41 and MOLT4, which had much higher HDAC6 expression levels. Indeed, the sensitivity of HDAC6 inhibitor KT-531 exhibits significant correlation with the available HDAC6 mRNA expression levels in T-cell lines (*R*^2^ = 0.9119, Figure S8b).

**Figure 6:**
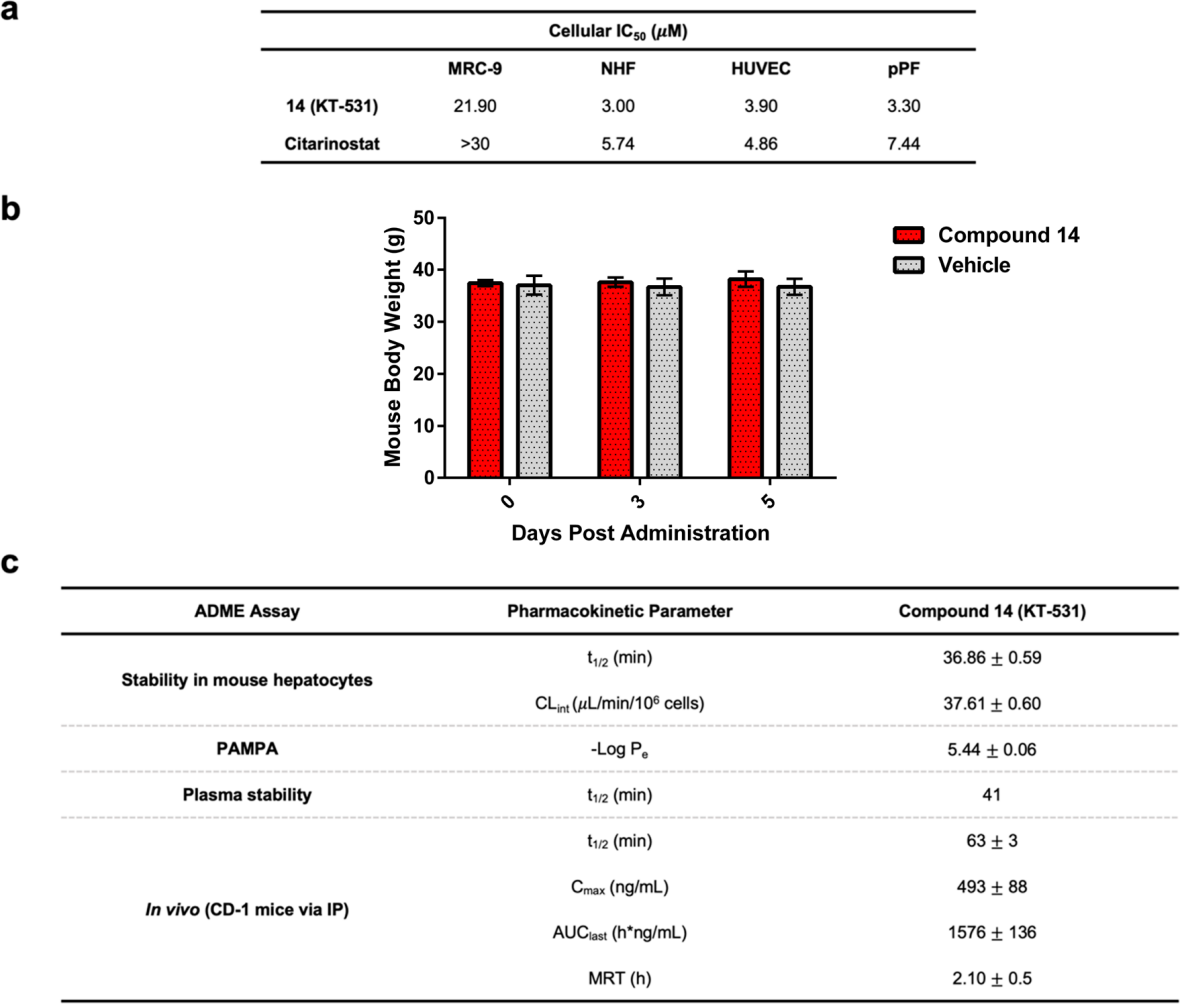
Preclinical findings show promising therapeutic window and pharmacokinetic properties of **14** (KT-531). (a) Cytotoxicity of KT-531 (**14**) and citarinostat in healthy noncancerous cell lines (MRC-9, normal human fibroblasts (NHF), human umbilical vein endothelial cells (HUVEC), primary pooled fibroblasts from 250 donors (pPF)). (b) Average mouse weight of CD-1 mice (20 mg/kg, P.O.) dosed with KT-531 (**14**) and vehicle daily for 5 days. (c) Pharmacokinetic assessment of KT-531 (**14**) in mouse hepatocytes, PAMPA assay, mouse plasma and *in vivo* in CD-1 mice (IP, 20 mg/kg).

The inhibitors were also assessed in nontransformed cellular systems to determine their therapeutic window. KT-531 displayed an IC_50_ of 22 μM in MRC-9 (lung) cells, revealing a clear therapeutic window. This supports the improved safety profile of HDAC6-targeting inhibitors compared to pan-HDACi. NHF (normal human fibroblasts), HUVEC (primary human umbilical vein endothelial cells), and pPF (primary pooled fibroblasts from 250 donors) were also assessed against citarinostat and KT-531, and while still possessing a therapeutic window both molecules were significantly more potent in these normal cell types (Figure 6a). The cellular safety margins for KT-531 are comparable to those of citarinostat and the FDA-approved HDACi SAHA (vorinostat) and belinostat (Figure 6a, Table S4). In addition, the safety of KT-531 was tested *in vivo* with a 5 day treatment of CD-1 mice (20 mg/kg via P.O. (oral administration)), which revealed no visible signs of toxicity or weight loss (Figure 6b).

### HDAC Expression in T-PLL Tumor Cells

**Figure 7:**
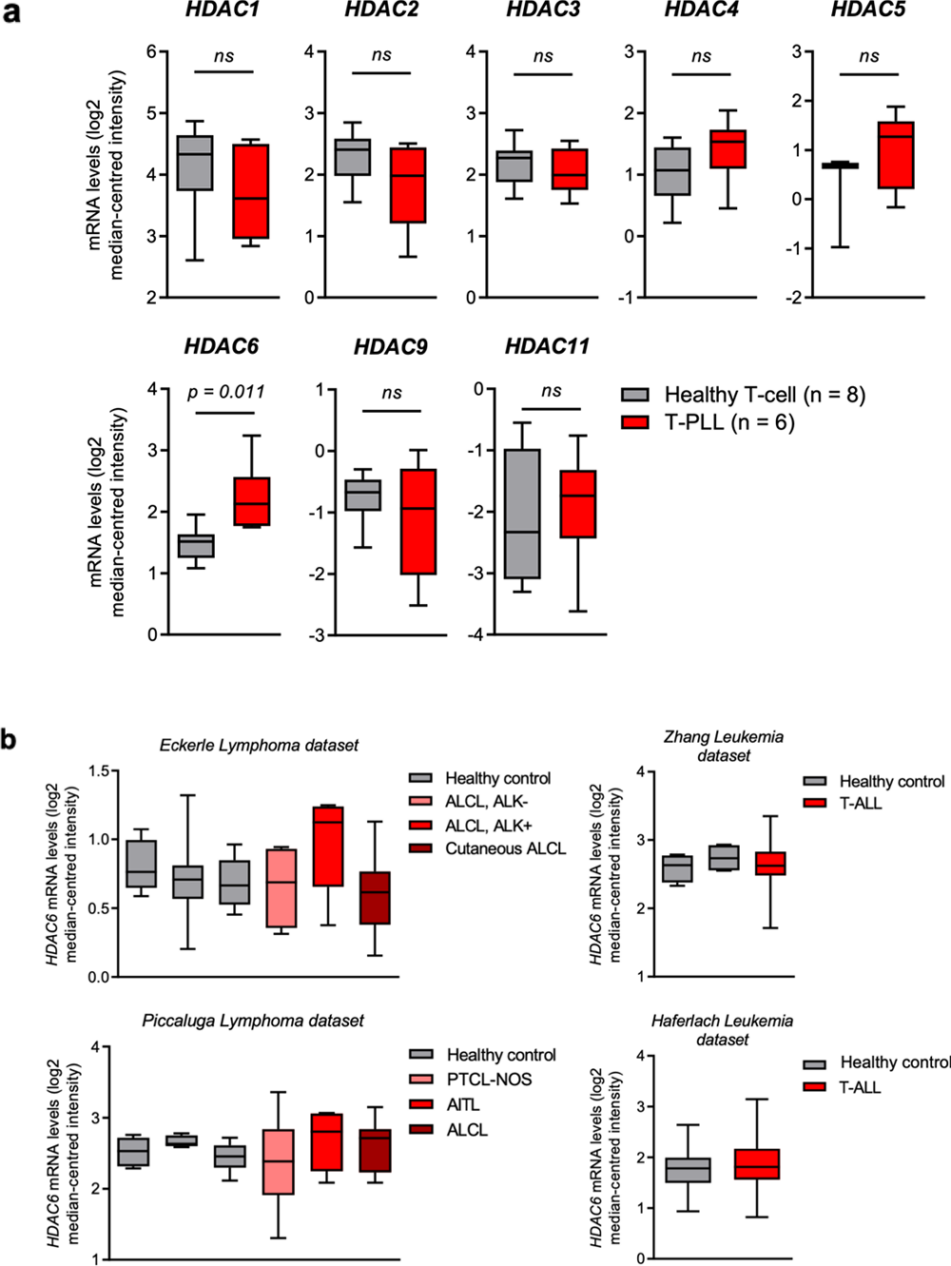
HDAC6 is significantly overexpressed in T-PLL patient samples, but not in other T-cell cancers. (a) mRNA expression levels of various *HDAC* genes (1–6, 9, and 11) in T-cell prolymphocytic leukemia (T-PLL) patient samples, compared to healthy T-cell (CD3^+^) control samples. Data were extracted from the Oncomine Platform, from the Durig Leukemia data set. *HDAC7*, *8*, and *10* expression levels were not reported in this data set. ns; not significant (*p* > 0.05). (b) mRNA expression levels of *HDAC6* in various T-cell neoplasia patient samples, compared to healthy control cells (from left to right, Eckerle Lymphoma: NK cell, T-cell, NK/T-cell; Piccaluga Lymphoma: CD4^+^ T-cell, CD8^+^ T-cell, T-cell; Zhang Leukemia: hematogone, bone marrow; Haferlach Leukemia: peripheral blood mononuclear cells). Data were extracted from the Oncomine Platform, from the Eckerle Lymphoma, Piccaluga Lymphoma, Zhang Leukemia, and Haferlach Leukemia data sets. All comparisons have a *p*-value > 0.05 and/or a fold-change of ≤1.2 and are not considered significant. Representation: boxes as interquartile range, horizontal line as the mean, and whiskers as lower and upper limits. Abbreviations: ALCL, anaplastic large cell lymphoma; ALK, anaplastic lymphoma kinase; PTCL-NOS, peripheral T-cell lymphoma, not otherwise specified; AITL, angioimmunoblastic T-cell lymphoma; NK, natural killer.

Oncomine database mining of published T-PLL patient gene expression data in relation to healthy T-cells revealed specific overexpression of *HDAC6* (1.7-fold) compared to other HDAC family members in T-PLL (Figure 7a).^46,59^ Interestingly, no increase in *HDAC6* expression levels was observed in available data sets of patients with other mature or immature T-cell malignancies, including anaplastic large cell lymphoma (ALCL), angioimmunoblastic T-cell lymphoma (AITL), PTCL not otherwise specified (PTCL-NOS), and T-ALL (Figure 7b), suggesting that *HDAC6* overexpression may be specific to T-PLL cancer. These data support the hypothesis that HDAC6 could be a beneficial target in T-PLL, and adverse effects of pan-HDAC inhibition could be minimized through the specific abrogation of HDAC6.

### KT-531 Shows Anticancer Activity and Therapeutic Margin in T-PLL Patient Samples

A high-throughput *ex vivo* drug sensitivity and resistance testing (DSRT) platform covering 306 approved and investigational oncology drugs has identified a potential therapeutic role for HDACi in T-PLL.^61^ Drug sensitivity scores (DSS) that capture and integrate multiparametric dose–response relationships into a single metric have previously identified T-PLL selective drug responses of FDA-approved HDACi panobinostat and belinostat.^61,62^ Therefore, we performed similar studies with KT-531 on primary T-PLL patient samples.

**Figure 8:**
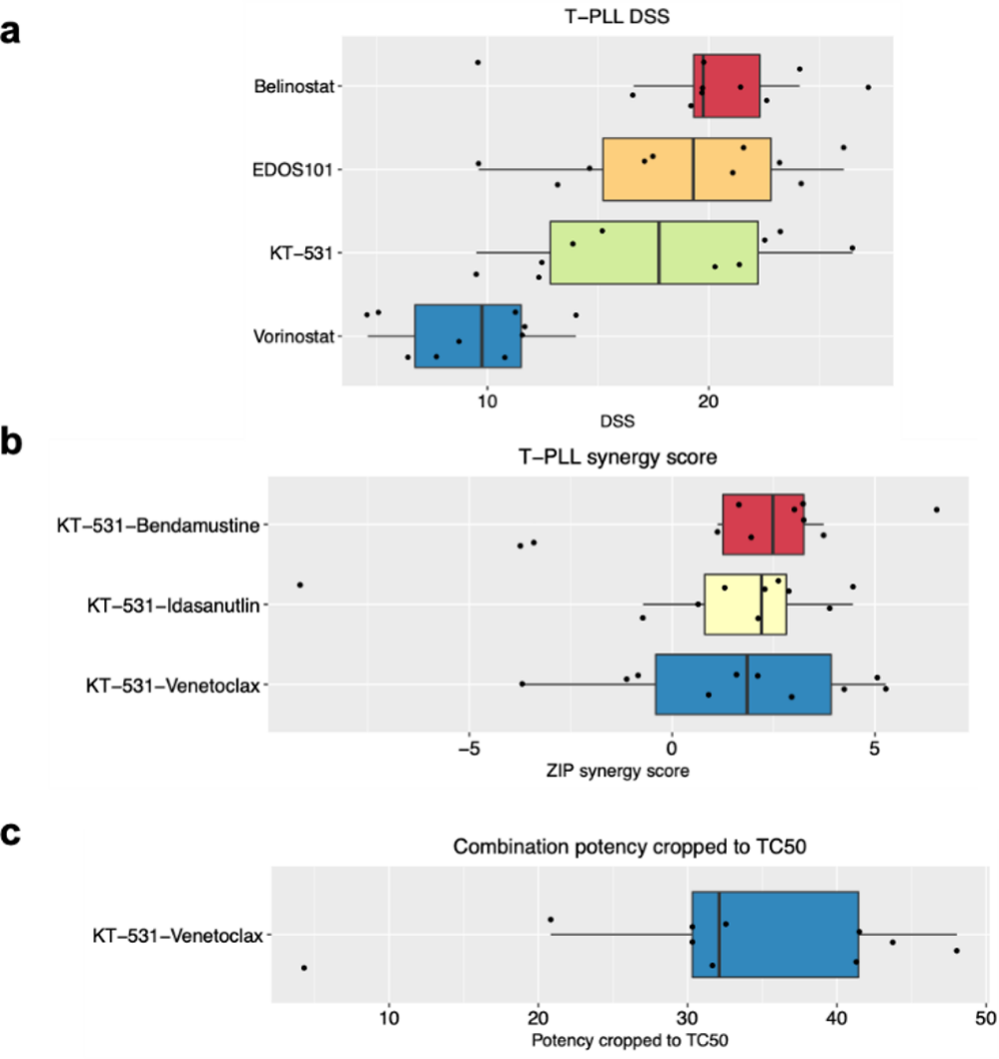
KT-531 (**14**) shows promising efficacy and safety in T-PLL primary patient samples. (a) Drug sensitivity scores (DSS)^62^ of selected HDACi in T-PLL primary patient samples. (b) Box plot of T-PLL synergy scores of KT-531 (**14**) with bendamustine, idasanutlin, and venetoclax in T-PLL patient samples. (c) Box plot of combination potency cropped to TC50 (toxic concentration in 50% of PBMC population) of KT-531 (**14**) and venetoclax in T-PLL patient samples and healthy PBMC controls.

In comparison to the pan-HDACi, vorinostat (SAHA), KT-531 resulted in a more potent drug response (*p* = 0.0007; Wilcoxon test), which was also comparable to other pan-HDACi, belinostat and tinostamustine (EDOS101) (Figure 8a). Anticancer effects of HDACi have been synergistically potentiated by other chemotherapeutic agents in hematopoietic cancers.^63−66^ Combinations of KT-531 with promising investigational drugs for T-PLL, notably idasanutlin, bendamustine, and venetoclax, were performed to identify potential synergisms. All combinations revealed synergistic relationships in patient samples, highlighting that HDAC inhibition may offer potential advantages in T-PLL (Figure 8b).^67^ Comparison of the potency in T-PLL patient samples to potency in PBMCs from healthy donors revealed a therapeutic margin for this novel combination of HDACi and venetoclax (Figure 8c). Collectively, the data supports the hypothesis that HDAC6 inhibition, alone or in drug combination, could be a promising therapy for T-PLL.

### Pharmacokinetic Studies

A preliminary *in vitro* ADME assessment of selected inhibitors was conducted. The pentafluorobenzene-sulfonamide analogue (**5**) exhibited a poor stability profile (*t*_1/2_ = ∼20 min in incubation with GSH, *t*_1/2_ < 2.26 min in mouse hepatocytes, *t*_1/2_ = 3.84 min in human hepatocytes, Table S1b). However, the removal of more than one fluorine from **5** was shown to be detrimental for antileukemic activity (**6**–**9**), leaving KT-531 as the most prominent lead for initial PK evaluation. Notably, no discernible S_N_Ar reaction with GSH was observed with KT-531 within 24 h of incubation (Table S1a).

The parallel artificial membrane permeability assay (PAMPA) and human plasma were used to evaluate the permeability and stability of compound KT-531, respectively. A −log *P*_e_ value of 5.44 was reported for KT-531 in the PAMPA assay, and *t*_1/2_ of 37 min in mouse hepatocytes and 41 min in human plasma showed promise for *in vivo* bioavailability (Figure 6c). *In vivo* pharmacokinetic studies on KT-531 were performed via intraperitoneal (IP) administration of compound in male CD-1 mice, at a dose of 20 mg/kg. Compound KT-531 reached a maximum concentration (*C*_max_) of 493 ng/mL, with *t*_1/2_ of 63 min and an overall exposure of 1576 h·ng/mL (Figure 6c). In comparison, and under the same conditions, citarinostat was reported to have a shorter *t*_1/2_ of 15 min but higher *C*_max_ and overall exposure (*C*_max_ = 5640 ng/mL, AUC = 4163 h·ng/mL; Figure S9), supporting the hypothesis that citarinostat is a more readily absorbed compound.

## Discussion and Conclusion

T-PLL is a very aggressive T-cell malignancy that does not benefit from “classical treatments” and urgently demands rationally designed targeted therapies. Available treatment options do not prevent inevitable patient relapses, and a novel treatment approach specifically tailored to treat T-PLL may resolve the hurdles of short survival (<20 months) and absence of curative therapy. The promising efficacy but limited tolerability of pan-HDAC inhibitors in this group of patients highlights the need to selectively and safely target the T-PLL-relevant HDAC isoforms. Critically, only the *HDAC6* isozyme of the HDAC family of enzymes was found to be overexpressed in primary T-PLL patient samples.

A comprehensive SAR study led to the identification of a preclinical candidate, KT-531 which demonstrated exceptional anticancer effects in T-PLL. This lead candidate exhibited (i) nanomolar inhibition of HDAC6 in a functional EMSA assay (IC_50_ = 8.5 nM), (ii) 39-fold *in vitro* selectivity for HDAC6 over other HDAC isozymes, and (iii) inhibition of cellular proliferation in a variety of hematologic model cell lines (AML, PTCL, T-ALL) while possessing a therapeutic window in heathy cells (MRC-9, NHF, pPF, HUVEC) and safety in 5 day toxicity trials in CD-1 mice. Since HDAC8 was the next nearest target of KT-531, HDAC8 selective inhibitors published in the literature were tested and used as control compounds to ensure that biological activity is not reliant on HDAC8 inhibition. Both PCI-34051 (120-fold selective for HDAC8) and MMH-410 (15-fold selective for HDAC8) exhibited nanomolar HDAC8 potency but no observable activity in MV4-11 and MRC9 cells (Table S5).^68,69^ Given the established pathogenetic role of HDAC modulation in AML and PTCL, cell lines representing these disease types were used to profile the library and investigate the cellular pharmacology of KT-531.

The promising cellular potency of KT-531 in the T-PLL quasi-model SUP-T11 prompted further investigation in this novel indication. The absence of activity in some branches of hematological neoplasms, such as CTCL, indicated that the higher selectivity of KT-531 in contrast to FDA-approved pan-HDAC drugs may be driving its unique and selective cytotoxicity profile. KT-531 exhibited comparable potency to clinically used HDACi, belinostat and vorinostat, in T-PLL patient cells, with a significant therapeutic window when compared to cytotoxicity in treated PBMCs from healthy donors. Given the well-validated synergism displayed by epigenetic agents with other compounds, KT-531 was tested in combination with promising investigational drugs for T-PLL, namely, idasanutlin, bendamustine, and venetoclax. The marked synergy of all pairings supports the clinical combination of more selective HDAC6i with these chemotherapeutic agents.

When coupled with the reported increased frequency of upregulated *HDAC6*, as compared to other HDAC isoforms in primary T-PLL patient cells, HDAC6i is a potential therapeutic avenue in this incurable condition and warrants more in-depth exploration. Further studies are necessary to explain the superior effects of KT-531 over citarinostat. Although the *in cellulo* and *in vitro* studies support KT-531 as a more HDAC6-selective inhibitor than citarinostat, the ∼39-fold HDAC6 enzymatic selectivity of KT-531 is not fully translated with same selectivity margins in cells. This is evident from the Western blots, where acetylation levels of histone H3 were concomitant with acetylation levels of α-tubulin, suggesting that the mechanism of action may not be solely dependent on HDAC6 inhibition. Such weak cellular selectivity profiles are common, as observed in multiple clinical trials of the modestly HDAC6-selective drug candidate ricolinostat where acetylation of histones was similar to acetylation of α-tubulin.^20,26^ Based on the data generated, an HDACi combination approach will be investigated in preclinical models of T-PLL, specifically through development of HDAC6 inhibitors with greater cellular selectivity and enhanced pharmacokinetic properties. Subsequent studies will be focused on deciphering the functional significance and mechanism of action of selective HDAC6 inhibition in T-PLL.

## Experimental Section

### General Procedures

All solvents and chemicals were used as purchased without further purification. Reactions were carried out in oven-dried glassware and monitored by thin-layer chromatography (TLC) using Merck silica gel 60 F_254_ on aluminum sheets (visualized by 254/365 nm UV light and/or staining with KMnO_4_). Column chromatography was carried out using Biotage Isolera One and Isolera Prime purification systems, with industry-standard SNAP 10, 25, 50, and 100 g cartridges loaded with 40–60 μm silica gel from VWR International (average pore size 60 Å), eluting at 12–40 mL/min and detecting compounds by UV measuring at 254 and 298 nm. Semipreparative HPLC was conducted using a Waters 2487 Dual λ Absorbance Detector, equipped with a Symmetry C18 4.6 mm × 150 mm cartridge. Compounds were detected by UV at 254 nm with sensitivity set to between 0.0001 and 4.0000 AUFS (default settings), eluting at 20 mL/min and using gradient mixtures of (A) Milli-Q water with 0.1% (v/v) TFA and (B) HPLC-grade acetonitrile.

Inhibitor purity was evaluated by using a Hewlett-Packard Series 1100 analytical HPLC system fitted with a Phenomenex Luna 5.0 μm C18 4.6 mm × 150 mm cartridge, with eluent flow set at 1.200 mL/min and using gradient mixtures of (A) Milli-Q water with 0.1% (v/v) TFA and (B) HPLC-grade acetonitrile. Retention times for the target compound are given, followed by purities in their respective order. Biologically evaluated compounds are ≥95% chemically pure.

A 400 MHz Bruker NMR instrument was utilized to obtain ^1^H, ^13^C, and ^19^F NMR spectra in CDCl_3_ (99.8 atom % D), CD_3_CN (99.8 atom % D), or MeOH-*d*_4_ (99.8 atom % D), as indicated (^1^H at 400 MHz, ^13^C at 100 MHz, and ^19^F at 54 MHz, unless otherwise stated). Samples were analyzed with 16 scans for ^1^H, 256 scans for ^13^C, and 8 scans for ^19^F, in 8” 3 mm A-grade glass tubes. Chemical shifts (δ) are reported in parts per million (ppm), after calibration to residual isotopic solvent, and coupling constants (*J*) are reported in hertz (Hz). Multiplicities are described as singlet (s), doublet (d), triplet (t), quartet (q), pentet (p), sextet (sex), septet (sep), multiplet (m), broad (br), or a combination of these. Low-resolution mass spectrometry (LRMS) was carried out using a Waters LC-MS system in ESI mode, fitted with a Micromass ZQ MS and an Alliance 2690 LC. Analysis was performed on a Waters Xterra C18 3.0 × 150 mm column containing 3.5 μm beads by direct injection into the mass spectrometer. High-resolution mass spectrometry (HRMS) was carried out using an Agilent 6538 UHD Q-TOF MS system in ESI mode with a mass accuracy ±1 mDa.

### Synthetic Procedures

#### Reductive Amination (a)

The appropriate alkyl amine (1.1 equiv) was added in one go to a solution of the appropriate aldehyde (1.0 equiv) in 1,2-DCE (0.1 M) and stirred at RT for 10 min before addition of glacial acetic acid (2.0 equiv) and further stirring for 30 min. Sodium triacetoxyborohydride (2.0 equiv) was added in one go, and the reaction was stirred for 24 h before pouring over saturated aqueous sodium bicarbonate. The layers were separated, and the aqueous layer was extracted with CH_2_Cl_2_. The combined organic layer was washed with saturated aqueous sodium bicarbonate, followed by brine, dried (MgSO_4_), filtered, and concentrated *in vacuo*. Column chromatography isolated the target compound.

#### Amine Sulfonylation (b)

The sulfonyl chloride (1.0–1.1 equiv) was added in one go to an ice-cooled solution of the appropriate amine (1.1 equiv) and triethylamine or DIPEA (2.0 equiv) in THF, CH_2_Cl_2_, or CH_3_Cl (0.1–0.2 M) and stirred at room temperature, and the disappearance of the limiting agent was monitored by TLC (hexanes/EtOAc 8:2). After 3–16 h, the reaction was quenched with 1 M HCl. The layers were separated, and the aqueous layer was extracted with CH_2_Cl_2_. The organic layer was dried over MgSO_4_, filtered, concentrated *in vacuo*, and purified by column chromatography to isolate the target compound.

#### Benzyl Deprotection by Hydrogenation (c)

To a nitrogen-purged solution of the benzyl or hydroxamate ester (1.0 equiv) in 2:1 mixture of THF and methanol or 100% methanol (0.05–0.1 M) was charged 10% Pd/C (0.1 equiv). The flask was purged with hydrogen, and then the reaction was stirred for 2 h under hydrogen atmosphere (balloon) before filtration through Celite, washed with THF, and concentrated *in vacuo*. Carboxylic acids were isolated without further purification. Preparative HPLC was used to isolate hydroxamic acids.

#### SN_2_ Substitution with Benzyl Bromides/Iodides (d)

Benzyl bromide/iodide (1.0 equiv) was added to a solution of the amine (1 equiv) and Cs_2_CO_3_ (1.5 equiv) in DMF (0.1 M). After 3–16 h (reaction monitored by TLC, hexanes/EtOAc 6:4). The reaction mixture was then diluted in EtOAc and saturated aqueous sodium bicarbonate. The organic layer was washed with saturated aqueous sodium bicarbonate (1×), water (3×), and brine (1×), and the aqueous layer was extracted once with EtOAc. The combined organic layer was dried over MgSO_4_, filtered, and concentrated *in vacuo* to isolate the target compound without further purification.

#### Acid-Mediated Hydrolysis of Carboxylate or Hydroxamate Esters (e)

The carboxylate or hydroxamate ester was charged in a round-bottom flask with 4 M HCl in dioxane (0.3 M final concentration) at RT in air. After 3–16 h, the solvent was removed *in vacuo*. Carboxylic acid intermediates were used in the next step without further purification, whereas final hydroxamic acids were purified using preparative HPLC.

#### Formation of Hydroxamate Esters (f)

Oxalyl chloride (4 equiv) was added dropwise to a solution of the appropriate carboxylic acid (1.0 equiv) in THF (0.05–0.2 M) and DMF (1 to 2 drops) at 0 °C and stirred for 50 min −3 h. The reaction was concentrated to dryness *in vacuo* before redissolving in dry THF (0.2 M) and mixing with diisopropylethylamine or triethylamine (2.0 equiv) followed by the *O*-protected hydroxylamine or its hydrochloride salt (1.0–2.0 equiv). After 16 h, the reaction was quenched with 1 M HCl and the layers were separated. The organic layer was washed with 1 M HCl and the combined aqueous layer was extracted with EtOAc or CH_2_Cl_2_. The organic layer was dried (MgSO_4_), filtered, and concentrated *in vacuo*, and purified by column chromatography to isolate the target compound.

#### Alternate Procedure for Formation of Hydroxamate Esters (f′)

Triethylamine (3.0 equiv) was charged in one go to a solution of the appropriate carboxylic acid (1.0 equiv), 1-(3-(dimethylamino)propyl)-3-ethylcarbodiimide hydrochloride (EDC.HCl, 1.5 equiv), and 1-hydroxybenzotriazole monohydrate (HOBt, 1.1 equiv) in DMF (0.1–0.2 M) at RT in air. After 10 min, the O-protected hydroxylamine (2.0 equiv) was added in one go, and the reaction was stirred for 16–24 h before quenching with 0.1 M HCl. The layers were separated, the organic layer was washed with 0.1 M HCl, and the aqueous layer was extracted with EtOAc or CH_2_Cl_2_. The organic layer was dried (MgSO_4_), filtered, and concentrated *in vacuo*, and purified by column chromatography to isolate the target compound.

#### Preparation of Sulfonyl Chlorides S3 from Polyhalogenated Benzene Rings (g)

The polyhalogenated benzene ring was added to neat chlorosulfonic acid (5 equiv) in a round-bottom flask under argon atmosphere, and the resulting solution stirred at RT for 5 min. The reaction was then heated to reflux (150 °C) for 2 h. The reaction turned dark over this period of time. The reaction was cooled to RT, and the crude material was added slowly to a mixture of ice and water. The aqueous phase was extracted three times with EtOAc, washed with brine, and concentrated to afford a dark brown oil that was flushed through a bed of silica and used without purification in subsequent step.

#### *tert-*Butyloxycarbonyl (Boc) Protection (h)

Di-*tert*-butyl dicarbonate (2.0 equiv) in THF (0.63 M) was added in one go to a solution of the appropriate amine hydrochloride (1.0 equiv) in THF and distilled water (1:1). Sodium bicarbonate (3.0 equiv) in distilled water (0.95 M) was added to the reaction mixture in one go and stirred at RT in air. After 19 h, the reaction was quenched by addition of 1 M HCl until pH 3 and the layers were separated. The aqueous layer was extracted with EtOAc and the combined organic layer was dried (MgSO_4_), filtered, and concentrated *in vacuo* to isolate the target compound without further purification.

#### Carboxyl Benzylation (i)

The appropriate benzoic acid (1.0 equiv) and cesium carbonate (1.1–1.2 equiv) were suspended in DMF (0.33–0.5 M) and stirred at RT for 20 min in air, before addition of benzyl bromide (1.0 equiv) in one go. After 24 h, the reaction was concentrated *in vacuo* and partitioned between EtOAc and distilled water. The layers were separated, and the aqueous layer was extracted with EtOAc. The combined organic layer was dried (MgSO_4_), filtered, and concentrated *in vacuo*. Column chromatography isolated the target compound.

#### Boc Deprotection (j)

The appropriate carbamate (1.0 equiv) was charged with ice-cooled 4 M HCl in dioxane (0.1 M) and stirred at RT in air. After 3 h, the solvent was removed *in vacuo*, azeotroped with CH_2_Cl_2_, to isolate the target compound without further purification.

### 2,3,4,5,6-Pentafluoro-*N*-(4-(hydroxycarbamoyl)benzyl)benzamide (**1**)

The product was obtained using synthetic procedure (c) as a white-solid (39%). ^1^H NMR δ/ppm (400 MHz, DMSO-*d*_*6*_) 4.54 (d, *J* = 5.9 Hz, 2H), 7.38 (d, *J* = 8.3 Hz, 2H), 7.74 (d, *J* = 8.3 Hz, 2H), 9.02 (s, 1H), 9.51 (t, *J* = 5.9 Hz, 1H), 11.19 (s, 1H). ^13^C NMR δ/ppm (100 MHz, DMSO-*d*_*6*_) 42.5, 127.1, 127.1, 131.7, 135.8, 138.1, 141.4, 141.9, 144.2, 156.8, 164.0. ^19^F NMR δ/ppm (54 MHz, DMSO-*d*_6_) −161.1 to −161.3 (m, 2F), −152.9 (t, *J* = 22.1 Hz, 1F), −142.0 to −142.2 (m, 2F). LRMS (ESI^+^) *m*/*z* calcd for [C_15_H_9_F_5_N_2_O_3_Na]^+^: 383.04, found: 383.16. HRMS (ESI^+^) *m*/*z* calcd for [C_15_H_10_F_5_N_2_O_3_]^+^: 361.0606, found: 361.0607. HPLC (I) *t*_R_ = 10.48 min (98.7%).

### *N*-Hydroxy-4-(((perfluorophenyl)sulfonamido)methyl)benzamide (**2**)

The product was obtained using synthetic procedure (e) after purification using preparative HPLC and lyophilization as a white powder (66.7%). ^1^H NMR (500 MHz, acetonitrile-*d*_3_) δ 10.73 (s, 1H), 7.78 (d, *J* = 8.4 Hz, 2H), 7.44 (d, *J* = 8.3 Hz, 2H), 4.47 (s, 2H), *hydroxamic acid OH, NH, and sulfonamide NH protons were not observed*. ^13^C NMR (126 MHz, cd_3_cn) δ 162.6, 140.0, 131.7, 130.1, 129.0, 126.5, 126.3, 125.0, 46.2. ^19^F NMR (376 MHz, acetone-*d*_6_) δ −136.68 to −139.04 (m, 2F), −151.43 to −153.09 (m, 1F), −161.00 to −162.87 (m, 2F). HRMS (ESI^+^) *m*/*z* calcd for [C_14_H_10_F_5_N_2_O_4_S]^+^: 397.03, found: 397.0269. HPLC (I) *t*_R_ = 16.99 min (97%).

### *N*-Hydroxy-4-(((2,3,4,5,6-pentafluoro-*N*-methylphenyl)sulfonamido)methyl)benzamide (**3**)

The product was obtained using synthetic procedure (c) after purification using preparative HPLC and lyophilization as a white powder (35.2%). ^1^H NMR (400 MHz, CD_3_CN) δ 7.83 (d, *J* = 8.4 Hz, 2H rotamer #1), 7.78 (d, *J* = 8.3 Hz, 2H rotamer#2), 7.47 (d, *J* = 8.3 Hz, 2H rotamer#2), 7.37 (d, *J* = 8.2 Hz, 2H rotamer#1), 4.48 (s, 2H, rotamer#2), 4.44 (s, 2H rotamer#1), 2.85 (s, 3H), *hydroxamic acid NH and OH protons were not observed*. ^13^C NMR (126 MHz, acetonitrile-*d*_3_) δ 160.6, 139.5, 136.6, 133.6, 131.5, 129.0, 128.2, 53.2, 34.1, 23.9. ^19^F NMR (376 MHz, chloroform-*d*) δ −134.19 to −136.14 (m, 2F), −146.11 (tt, *J* = 21.1, 6.7 Hz, 1F), −157.50 to −159.50 (m, 2F). HRMS (ESI^+^) *m*/*z* calcd for [C_15_H_12_F_5_N_2_O_4_S]^+^: 411.04; found 411.0425. HPLC (I) *t*_R_ = 14.54 min (97%).

### *N*-Hydroxy-4-(((2,3,4,5,6-pentafluoro-*N*-isopropylphenyl)sulfonamido)methyl)benzamide (**4**)

The product was obtained using synthetic procedure (e) after purification using preparative HPLC and lyophilization as a white powder (50%). ^1^H NMR (400 MHz, acetonitrile-*d*_3_) δ 9.76 (s, 1H), 7.72 (d, 2H), 7.51 (d, 2H), 4.60 (s, 2H), 4.31 (p, *J* = 6.8 Hz, 1H), 1.11 (d, *J* = 6.8 Hz, 6H), *hydroxamic acid OH proton was not observed*. ^13^C NMR (126 MHz, acetonitrile-*d*_3_) δ 165.1, 145.7, 143.7, 142.9, 130.9, 127.9, 127.0, 125.6, 117.4, 51.4, 46.3, 20.4. ^19^F NMR (376 MHz, acetonitrile-*d*_3_) δ −137.37 to −137.57 (m, 2F), −149.44 (tt, *J* = 20.2, 6.6 Hz, 1F), −161.26 to −161.46 (m, 2F). HRMS (ESI^+^) *m*/*z* calcd for [C_17_H_16_F_5_N_2_O_4_S]^+^: 439.07; found 439.0740. HPLC (I) *t*_R_ = 14.24 min (100%).

### 4-(((*N*-Cyclopropyl-2,3,4,5,6-pentafluorophenyl)sulfonamido)methyl)-*N*-hydroxybenzamide (**5**)

The product was obtained using synthetic procedure (c) after purification using preparative HPLC and lyophilization as a white powder (53%). ^1^H NMR (400 MHz, acetone-*d*_6_) δ: 10.83 (s, 1H), 7.87 (d, *J* = 8.3 Hz, 2H), [7.52 (d, *J* = 8.3 Hz, 2H rotamer #1), 7.46 (d, *J* = 8.0 Hz, 2H rotamer #2)], [4.68 (s, 2H rotamer #1), 4.65 (s, 2H rotamer #2)], 2.59–2.49 (m, 1H), 0.86–0.51 (m, 4H), *hydroxamic acid OH proton was not observed*. ^13^C NMR (101 MHz, acetone) δ 160.1, 144.4, 141.5, 133.9, 129.8, 128.0, 124.6, 54.2, 31.1, 13.1, 8.2. ^19^F NMR (376 MHz, CDCl_3_) δ: −134.84 to −135.26 (m, 2F), −145.36 (t, *J* = 20.5 Hz, 1F), −157.84 to −158.96 (m, 2F). HRMS (ESI^+^) *m*/*z* calcd for [C_17_H_14_F_5_N_2_O_4_S]^+^: 437.0589, found: 437.0588. HPLC (I) *t*_R_ = 20.23 min (97.5%).

### 4-(((*N*-Cyclopropyl-2,3,5,6-tetrafluorophenyl)sulfonamido)methyl)-*N*-hydroxybenzamide (**6**)

The product was obtained using synthetic procedure (e) after purification using preparative HPLC and lyophilization as a white powder (49%). ^1^H NMR (400 MHz, acetone-*d*_6_) δ 10.81 (s, 1H), 8.02–7.79 (m, 3H), 7.53 (d, *J* = 7.9 Hz, 2H rotamer#1), 7.46 (d, *J* = 7.7 Hz, 2H rotamer#2), 4.70 (s, 1H) (rotamer #1), 4.67 (s, 1H) (rotamer #2), 2.56 (p, *J* = 6.4, 3.5, 2.8 Hz, 1H), 0.80–0.61 (m, 4H), *hydroxamic acid OH proton was not observed*. ^13^C NMR (126 MHz, acetonitrile-*d*_3_) δ 161.8, 147.6, 140.9, 138.4, 131.2, 128.3, 127.2, 125.7, 120.5, 111.1, 53.7, 30.4, 6.4. ^19^F NMR (376 MHz, acetone-*d*_6_) δ −137.15 to −137.73 (m, 2F), −137.79 to −138.20 (m, 2F). LRMS (ESI^–^) *m*/*z* calcd for [C_17_H_13_F_4_N_2_O_4_S]^−^: 417.05, found: 417.48. HRMS (ESI^+^) *m*/*z* calcd for [C_17_H_14_F_4_N_2_O_4_S]^+^: 419.0683, found: 419.0677. HPLC (I) *t*_R_ = 20.29 min (100%).

### 4-(((*N*-Cyclopropyl-4-fluorophenyl)sulfonamido)methyl)-*N*-hydroxybenzamide (**7**)

The product was obtained using synthetic procedure (c) after purification using preparative HPLC and lyophilization as a white powder (59%). ^1^H NMR (400 MHz, acetone-*d*_6_) δ: 10.28 (s, 1H), 8.03 (d, *J* = 8.9, 5.2 Hz, 2H), 7.85 (d, *J* = 8.3 Hz, 2H), 7.55 (d, *J* = 8.2 Hz, 2H), 7.45 (d, *J* = 8.8 Hz, 2H), 4.52 (s, 2H), 2.26–2.19 (m, 1H), 0.79–0.68 (m, 4H), *hydroxamic acid OH proton was not observed*. ^13^C NMR (101 MHz, acetone-*d*_6_) δ: 130.7, 130.6, 128.5, 127.0, 116.4, 116.2, 54.1, 31.1, 6.8. ^19^F NMR (376 MHz, acetone-*d*_6_) δ: −107.29 to −107.46 (m, 1F). HRMS (ESI^+^) *m*/*z* calcd for [C_17_H_18_FN_2_O_4_S]^+^: 365.0966, found: 365.0969. HPLC (I) *t*_R_ = 23.04 min (99%).

### 4-(((*N*-Cyclopropyl-3,4,5-trifluorophenyl)sulfonamido)methyl)-*N*-hydroxybenzamide (**8**)

The product was obtained using synthetic procedure (c) after purification using preparative HPLC and lyophilization as a white powder (60%). ^1^H NMR (400 MHz, acetone-*d*_6_) δ: 10.83 (s, 1H), 7.84 (d, *J* = 8.3 Hz, 2H), 7.79–7.69 (m, 2H), [7.48 (d, *J* = 8.3 Hz, 2H rotamer #1), 7.42 (d, *J* = 8.0 Hz, 2H rotamer #2)], [4.53 (s, 2H rotamer #1), 4.50 (s, 2H rotamer #2)], 2.35–2.25 (m, 1H), 0.77–0.64 (m, 4H), *hydroxamic acid OH proton was not observed*. ^13^C NMR (101 MHz, acetone) δ 202.5, 170.7, 152.6, 142.0, 136.9, 129.3, 127.9, 114.0, 113.7, 55.0, 31.9, 7.6. ^19^F NMR (376 MHz, acetone-*d*_6_) δ: −132.40 to −132.79 (m, 1F), −155.19 to −155.55 (m, 2F). HRMS (ESI^+^) *m*/*z* calcd for [C_17_H_16_F_3_N_2_O_4_S]^+^: 401.0777, found: 401.0772. HPLC (I) *t*_R_ = 26.05 min (98.8%).

### 4-(((*N*-Cyclopropyl-2,4,6-trifluorophenyl)sulfonamido)methyl)-*N*-hydroxybenzamide (**9**)

The product was obtained using synthetic procedure (c) after purification using preparative HPLC and lyophilization as a white powder (63%). ^1^H NMR (400 MHz, acetone-*d*_6_) δ: 10.86 (s, 1H), 7.89–7.82 (m, 2H), 7.52 (d, *J* = 8.0 Hz, 1H), 7.46 (d, *J* = 7.9 Hz, 1H), 7.28–7.18 (m, 2H), [4.66 (s, 2H rotamer #1), 4.63 (s, 2H rotamer #2)], 2.62–2.37 (m, 1H), 0.70–0.62 (m, 4H), *hydroxamic acid OH proton was not observed*. ^13^C NMR (101 MHz, acetone-*d*_6_) δ: 160.5, 141.2, 138.8, 131.6, 128.3, 128.2, 127.1, 125.7, 102.8, 53.6, 30.1, 6.2. ^19^F NMR (376 MHz, acetone-*d*_6_) δ: −100.83 to −101.14 (m, 1F), −103.06 (dt, *J* = 39.8, 10.5 Hz, 2F). HRMS (ESI^+^) *m*/*z* calcd for [C_17_H_16_F_3_N_2_O_4_S]^+^: 401.0777, found: 401.0772. HPLC (I) *t*_R_ = 15.16 min (99%).

### 3-(((*N*-Cyclopropyl-2,3,4,5,6-pentafluorophenyl)sulfonamido)methyl)-*N*-hydroxybenzamide (**10**)

The product was obtained using synthetic procedure (e) after purification using preparative HPLC and lyophilization as a white powder (66.7%). ^1^H NMR (400 MHz, MeOD) δ 7.78 (s, 1H), 7.70 (d, *J* = 7.7, 1.5 Hz, 1H), 7.61 (d, *J* = 7.7, 1.6 Hz, 1H), 7.48 (dd, *J* = 7.7 Hz, 1H), 4.64 (s, 2H), 2.48 (p, *J* = 6.4, 3.2 Hz, 1H), 0.80–0.61 (m, 4H). *hydroxamic acid OH and NH protons were not observed*. ^13^C NMR (101 MHz, chloroform-*d*) δ 139.4, 135.6, 130.8, 87.8, 31.6, 30.9, 30.4, 22.7, 14.1, 7.0. ^19^F NMR (376 MHz, chloroform-*d*) δ −134.85 (2F), −145.19 (1F), −158.25 (d, *J* = 18.1 Hz, 2F). HRMS (ESI^+^) *m*/*z* calcd for [C_17_H_13_F_5_N_2_O_4_S]^+^: 436.05, found: 437.05, HPLC (I) *t*_R_ = 19.59 min (98.1%).

### 4-(((3,5-Dichloro-*N*-cyclopropyl-2,4,6-trifluorophenyl)sulfonamido)methyl)-*N*-hydroxybenzamide (**11**)

The product was obtained using synthetic procedure (e) after purification using preparative HPLC and lyophilization as a white powder (81%). ^1^H NMR (400 MHz, CD_3_CN) δ 9.91 (s, 1H), 7.75 (d, *J* = 7.8 Hz, 2H), 7.48 (d, *J* = 7.7 Hz, 2H), 4.63 (s, 2H), 2.49 (p, *J* = 6.6, 3.5 Hz, 1H), 0.74–0.65 (m, 4H), *hydroxamic acid OH proton was not observed*. ^13^C NMR (101 MHz, CD_3_CN) δ 177.2, 157.5, 157.1, 153.2, 140.3, 129.2, 128.1, 54.7, 31.4, 7.4. ^19^F NMR (376 MHz, CD_3_CN) δ −105.08 (t, *J* = 6.9 Hz, 1F), −107.87 (d, *J* = 6.9 Hz, 2F). HRMS (ESI^+^) *m*/*z* calcd for [C_17_H_14_F_3_Cl_2_N_2_O_4_S]^+^: 468.9998, found: 468.9997. HPLC (I) *t*_R_ = 20.78 min (100%).

### 4-(((3-Chloro-*N*-cyclopropyl-2,4,6-trifluorophenyl)sulfonamido)methyl)-*N*-hydroxybenzamide (**12**)

The product was obtained using synthetic procedure (e) after purification using preparative HPLC and lyophilization as a white powder (77%). ^1^H NMR (400 MHz, CD_3_CN) δ: 7.75 (d, *J* = 7.8 Hz, 2H), 7.48 (d, *J* = 7.6 Hz, 2H), 7.29–7.16 (m, 1H), 4.61 (s, 2H), 2.59–2.40 (m, 1H), 0.77–0.56 (m, 4H), *hydroxamic acid OH proton was not observed*. ^13^C NMR (101 MHz, CD_3_CN) δ 148.0, 145.6, 144.6, 141.5, 132.1, 129.8, 128.1, 104.5, 104.0, 103.6, 54.6, 30.8, 6.7. ^19^F NMR (376 MHz, CD_3_CN) δ: −104.04 (q, *J* = 7.5 Hz, 1F), −105.46 (t, *J* = 10.4 Hz, 1F), −105.71 (d, *J* = 7.7 Hz, 1F). HRMS (ESI^+^) *m*/*z* calcd for [C_17_H_15_F_3_ClN_2_O_4_S]^+^: 435.0388, found: 435.0392. HPLC (I) *t*_R_ = 19.19 min (99.2%).

### 4-(((4-Chloro-*N*-cyclopropyl-2,3,5,6-tetrafluorophenyl)sulfonamido)methyl)-*N*-hydroxybenzamide (**13**)

The product was obtained using synthetic procedure (e) after purification using preparative HPLC and lyophilization as a white powder (71%). ^1^H NMR (400 MHz, acetone) δ 7.87 (d, *J* = 7.8 Hz, 2H), 7.52 (d, *J* = 7.6 Hz, 2H), 4.69 (s, 2H), 2.89–2.45 (m, 1H), 1.05–0.52 (m, 4H). ^19^F NMR (376 MHz, acetone) δ −136.45 to −136.81 (m, 2F), −140.16 to −140.46 (m, 2F). ^13^C NMR (101 MHz, acetone) δ 146.0, 145.8, 143.0, 140.7, 131.7, 128.3, 127.2, 118.6, 53.7, 30.3, 6.4. HRMS (ESI^+^) *m*/*z* calcd for [C_17_H_14_F_4_ClN_2_O_4_S]^+^: 453.0293, found: 453.0294. HPLC (I) *t*_R_ = 20.45 min (100%).

### 4-(((*N*-Cyclopropyl-2,3,4,5-tetrafluorophenyl)sulfonamido)methyl)-*N*-hydroxybenzamide (**14**)

The product was obtained using synthetic procedure (e) after purification using preparative HPLC and lyophilization as a white powder (60%). ^1^H NMR (400 MHz, DMSO) δ 11.28 (s, 1H), 8.04–7.84 (m, 1H), 7.81–7.69 (m, 2H), 7.58–7.20 (m, 2H), 4.59 (s, 2H), 2.48 (p, *J* = 7.1, 3.9 Hz, 1H), 0.76–0.61 (m, 4H), *hydroxamic acid OH proton was not observed*. ^13^C NMR (126 MHz, acetone) δ 141.0, 132.5, 130.8, 129.2, 129.1, 128.0, 126.2, 113.7, 113.5, 53.5, 31.2, 31.0, 28.1, 7.4. ^19^F NMR (376 MHz, acetone-*d*_6_) δ: −134.84 (ddt, *J* = 21.3, 14.0, 7.4 Hz, 1F), −138.26 to −138.43 (m, 1F), −149.58 (tt, *J* = 20.0, 8.2 Hz, 1F), −153.95 (ddt, *J* = 21.9, 19.2, 3.4 Hz, 1F). HRMS (ESI^+^) *m*/*z* calcd for [C_17_H_15_F_4_N_2_O_4_S]^+^: 419.0681, found: 419.0683. HPLC (I) *t*_R_ = 20.43 min (100%).

### Intermediate Compound Characterization for All New Molecules

#### Benzyl 4-Formylbenzoate (**S1**)

The product was obtained using synthetic procedure (i) as a light yellow oil (70%). ^1^H (400 MHz, CDCl_3_) δ: 10.10 (s, 1H); 8.23 (d, *J* = 8.3 Hz, 2H), 7.95 (d, *J* = 8.5 Hz, 2H), 7.35–7.47 (m, 5H), 5.40 (s, 2H). ^13^C (101 MHz, CDCl_3_) δ: 191.6, 165.4, 139.2, 135.6, 135.1, 130.3, 128.7, 128.5, 128.4, 67.3. LRMS (ESI) *mass not observed*.

#### Benzyl 4-((Cyclopropylamino)methyl)benzoate (**S2**)

The product was obtained using synthetic procedure (a) as a pale yellow oil (83%). ^1^H (400 MHz, CDCl_3_) δ: 8.07 (d, *J* = 8.2 Hz, 2H), 7.48 (d, *J* = 7.3 Hz, 2H), 7.45–7.36 (m, 5H), 5.39 (s, 2H), 3.92 (s, 2H), 2.16 (dd, *J* = 6.6, 3.7 Hz, 1H), 0.46 (s, 2H), 0.41 (s, 2H). ^13^C (101 MHz, CDCl_3_) δ: 166.4, 146.2, 136.2, 129.8, 128.7, 128.6, 128.2, 128.1, 66.6, 53.4, 53.1, 51.6. LRMS (ESI+) *m*/*z* calcd for [C_18_H_20_NO_2_]^+^: 282.14, found: 282.30.

#### *N*-Cyclopropyl-2,3,4,5,6-pentafluorobenzenesulfonamide (**S9a**)

The product was obtained using synthetic procedure (b) as a clear oil without further purification (80%). ^1^H NMR (400 MHz, CDCl_3_) δ: 5.47 (s, 1H), 2.47–2.39 (m, 1H), 0.82–0.70 (m, 4H). ^13^C NMR (101 MHz, CDCl_3_) δ: 156.8, 145.5, 144.8, 116.5, 39.9, 27.8. ^19^F NMR (376 MHz, CDCl_3_) δ: −137.91 to −138.14 (m, 2F), −148.02 (s, 1F), −160.05 to −161.11 (m, 2F). LRMS (ESI+) *m*/*z* calcd for [C_9_H_6_F_5_NO_2_SNa]^+^: 309.99, found: 310.24.

#### *N*-Cyclopropyl-2,3,4,5-tetrafluorobenzenesulfonamide (**S9b**)

The product was obtained using synthetic procedure (b) as a yellow oil without further purification (83%). ^1^H NMR (400 MHz, CD_3_CN) δ: 7.92–7.30 (m, 1H), 6.40 (s, 1H), 2.34 (p, *J* = 6.9, 3.6 Hz, 1H), 0.65–0.53 (m, 4H). ^13^C NMR (101 MHz, CDCl_3_) δ: 156.8, 145.5, 144.8, 140.4, 117.3, 23.9, 4.9. ^19^F NMR (376 MHz, CDCl_3_) δ: −135.25 (s, 1F), −135.39 to −135.78 (m, 1F), −146.02 to −147.09 (m, 1F), −151.30 (s, 1F). LRMS (ESI-) *m*/*z* calcd for [C_9_H_6_F_4_NO_2_S]^−^: 268.01, found: 268.02.

#### 2,3,4,5-Tetrafluorobenzenesulfonyl Chloride Used for **S9b**

The product was obtained using synthetic procedure (g) as a clear, yellow oil without further purification (80%). ^1^H NMR (400 MHz, acetonitrile-*d*_3_) δ 7.92–7.66 (m, 1H). ^13^C NMR (101 MHz, acetonitrile-*d*_3_) δ 147.8, 147.3, 146.7, 145.3, 144.6, 140.5, 116.6. ^19^F NMR (376 MHz, acetonitrile-*d*_3_) δ −133.82 to −134.01 (m, 1F), −135.65 to −136.10 (m, 1F), −142.87 (s, 1F), −151.06 to −151.57 (m, 1F). LRMS (ESI+) found 229.04 (corresponding to M-1 for the acid showing rapid compound hydrolysis under MS conditions).

#### Benzyl 4-(((*N*-Cyclopropyl-2,3,4,5,6-pentafluorophenyl)sulfonamido)methyl)benzoate (**S4a**)

The product was obtained using synthetic procedure (b) as a viscous pale yellow oil (75%). ^1^H NMR (400 MHz, CDCl_3_) δ: 8.09 (d, *J* = 8.3 Hz, 2H), 7.53–7.46 (m, 4H), 7.45–7.35 (m, 3H), 5.40 (s, 2H), 4.62 (s, 2H), 2.39 (p, *J* = 5.3 Hz, 1H), 0.72 (s, 4H). ^13^C NMR (101 MHz, CDCl_3_) δ: 166.0, 141.4, 135.9, 130.1, 129.9, 128.6, 128.3, 128.3, 128.2, 66.8, 54.2, 30.4, 6.9. ^19^F NMR (376 MHz, CDCl_3_) δ: −134.77 (s, 2F), −145.18 to −145.41 (m, 1F), −158.10 to −158.36 (m, 2F). LRMS (ESI+) *m*/*z* calcd for [C_24_H_18_F_5_NO_4_SNa]^+^: 534.08, found: 534.32.

#### *tert*-Butyl 4-(((*N*-Cyclopropyl-2,3,4,5-tetrafluorophenyl)sulfonamido)methyl)benzoate (**S4b**)

The product was obtained using synthetic procedure (d) as a viscous pale yellow viscous oil (82%). ^1^H NMR (400 MHz, CDCl_3_) δ: 8.09 (d, *J* = 8.3 Hz, 2H), 7.68–7.55 (m, 1H), 7.51–7.45 (m, 4H), 7.45–7.33 (m, 3H), 5.40 (s, 2H), 4.61 (s, 2H), 2.32 (p, *J* = 6.8, 5.6, 4.5, 1.0 Hz, 1H), 0.65 (d, *J* = 4.4 Hz, 4H). ^13^C NMR (101 MHz, CDCl_3_) δ: 166.0, 141.7, 136.0, 130.3, 130.0, 129.8, 128.6, 128.3, 128.2, 128.1, 112.7, 66.7, 54.1, 30.3, 7.1. ^19^F NMR (376 MHz, CDCl_3_) δ: −133.29 (s, 1F), −134.73 to −136.53 (m, 1F), −145.42 to −146.92 (m, 1F), −150.93 to −151.01 (m, 1F). LRMS (ESI+) *m*/*z* calcd for [C_24_H_19_F_4_NO_4_SNa]^+^: 516.09, found: 516.36.

#### Benzyl 4-(((*N*-Cyclopropyl-3,4,5-trifluorophenyl)sulfonamido)methyl)benzoate (**S4c**)

The product was obtained using synthetic procedure (b) as a yellow oil (91%). ^1^H NMR (400 MHz, THF-*d*_8_) δ: 8.07 (d, *J* = 8.3 Hz, 2H), 7.76 (t, *J* = 6.6 Hz, 2H), 7.55–7.49 (m, 4H), 7.45–7.09 (m, 3H), 5.40 (s, 2H), 4.51 (s, 2H), 2.20 (tt, *J* = 7.0, 3.4 Hz, 1H), 0.85–0.48 (m, 2H). ^13^C NMR (101 MHz, THF-*d*_8_) δ: 165.2, 150.1, 143.7, 142.6, 141.2, 136.5, 135.1, 129.7, 129.5, 128.4, 128.3, 127.9, 112.9, 54.2, 27.7, 6.7. ^19^F NMR (376 MHz, THF-*d*_8_) δ: −132.18 (s, 1F), −154.98 (s, 2F). LRMS (ESI+) *m*/*z* calcd for [C_24_H_20_F_3_NO_4_SNa]^+^: 498.10, found: 497.60.

#### Benzyl 4-(((*N*-Cyclopropyl-2,4,6-trifluorophenyl)sulfonamido)methyl)benzoate (**S4d**)

The product was obtained using synthetic procedure (b) as a yellow oil (81%). ^1^H NMR (400 MHz, CDCl_3_) δ: 8.08 (d, *J* = 8.3 Hz, 2H), 7.51–7.45 (m, 2H), 7.44–7.33 (m, 2H), 6.81 (t, *J* = 8.6 Hz, 1H), 5.39 (s, 2H), 4.61 (s, 2H), 2.36 (p, *J* = 7.0, 3.9 Hz, 1H), 0.97–0.56 (m, 4H). ^13^C NMR (101 MHz, CDCl_3_) δ: 166.0, 142.3, 136.0, 129.9, 129.6, 128.6, 128.2, 128.1, 126.5, 120.8, 102.1, 66.7, 54.0, 30.3, 6.8. ^19^F NMR (376 MHz, CDCl_3_) δ: −98.44 (s, 1F), −101.59 (s, 2F). LRMS (ESI+) *m*/*z* calcd for [C_24_H_20_F_3_NO_4_SNa]^+^: 498.10, found: 498.30.

#### Benzyl 4-(((*N*-Cyclopropyl-4-fluorophenyl)sulfonamido)methyl)benzoate (**S4e**)

The product was obtained using synthetic procedure (b) as a yellow oil (92%). ^1^H NMR (400 MHz, THF) δ 8.07 (d, *J* = 8.3 Hz, 2H), 8.03–7.97 (m, 2H), 7.57–7.48 (m, 4H), 7.46–7.32 (m, 5H), 5.40 (s, 2H), 4.46 (s, 2H), 2.15–2.04 (m, 1H), 0.71–0.53 (m, 4H). ^13^C NMR (101 MHz, THF-*d*_8_) δ: 166.4, 165.3, 163.9, 136.6, 135.0, 130.5, 129.5, 129.5, 128.4, 128.0, 127.9, 116.1, 115.8, 54.1, 31.0, 6.6. ^19^F NMR (376 MHz, THF-*d*_8_) δ: −107.05 (s, 1F). LRMS (ESI+) *m*/*z* calcd for [C_24_H_22_FNO_4_SNa]^+^: 462.11, found: 462.46.

#### Benzyl 4-(((3,5-Dichloro-*N*-cyclopropyl-2,4,6-trifluorophenyl)sulfonamido)methyl)benzoate (**S4f**)

The product was obtained using synthetic procedure (b) as a yellow oil (61%). ^1^H NMR (400 MHz, CDCl_3_) δ: 8.09 (s, 2H), 7.57–7.44 (m, 4H), 7.45–7.34 (m, 3H), 5.40 (s, 2H), 4.63 (s, 2H), 2.41 (p, *J* = 5.8, 5.3 Hz, 1H), 0.99–0.20 (m, 4H). ^13^C NMR (101 MHz, CDCl_3_) δ: 130.0, 128.6, 128.2, 128.2, 126.7, 125.6, 123.4, 107.8, 67.9, 67.7, 29.1, 23.9, 7.0. ^19^F NMR (376 MHz, CDCl_3_) δ: −101.81 (s, 1F), −105.89 (s, 2F). LRMS (ESI) *mass not observed*.

#### Benzyl 4-(((4-Chloro-*N*-cyclopropyl-2,3,5,6-tetrafluorophenyl)sulfonamido)methyl)benzoate (**S4g**)

The product was obtained using synthetic procedure (b) as a yellow oil (61%). ^1^H NMR (400 MHz, CDCl_3_) δ: 8.09 (d, *J* = 8.3 Hz, 2H), 7.53–7.46 (m, 4H), 7.46–7.34 (m, 3H), 5.40 (s, 2H), 4.63 (s, 2H), 2.46–2.37 (m, 1H), 0.77–0.67 (m, 4H). ^13^C NMR (101 MHz, CDCl_3_) δ: 165.9, 141.4, 135.9, 134.5, 130.0, 129.9, 129.5, 128.6, 128.3, 128.2, 66.8, 54.2, 30.4, 7.0. ^19^F NMR (376 MHz, CDCl_3_) δ: −134.91 to −135.16 (m, 2F), −137.36 to −137.61 (m, 2F). LRMS (ESI+) *m*/*z* calcd for [C_24_H_18_ClF_4_NO_4_SNa]^+^: 550.05, found: 550.30.

#### 4-(((*N*-Cyclopropyl-2,3,4,5,6-pentafluorophenyl)sulfonamido)methyl)benzoic Acid (**S5a**)

The product was obtained using synthetic procedure (c) as a white solid (98%). ^1^H NMR (400 MHz, CDCl_3_) δ: 8.12 (d, *J* = 8.3 Hz, 2H), 7.52 (d, *J* = 8.1 Hz, 2H), 4.65 (s, 2H), 2.42 (p, *J* = 5.2 Hz, 1H), 0.83–0.63 (m, 4H). ^13^C NMR (101 MHz, DMSO-*d*_6_) δ: 167.5, 165.6, 165.4, 150.9, 142.1, 130.7, 130.0, 128.4, 53.6, 30.7, 6.8. ^19^F NMR (376 MHz, CDCl_3_) δ: −134.65 to −134.83 (m, 2F), −145.18 (s, 1F), −158.16 to −159.22 (m, 2F). LRMS (ESI-) *m*/*z* calcd for [C_17_H_11_F_5_NO_4_S]^−^: 420.03, found: 420.38.

#### 4-(((*N*-Cyclopropyl-3,4,5-trifluorophenyl)sulfonamido)methyl)benzoic Acid (**S5c**)

The product was obtained using synthetic procedure (c) as an amorphous solid in a quantitative yield. ^1^H NMR (400 MHz, DMSO-*d*_6_) δ: 7.92 (d, *J* = 8.0 Hz, 2H), 7.84 (m, 2H), 7.40 (d, *J* = 8.0 Hz, 2H), 4.46 (s, 2H), 2.25 (p, *J* = 8.8, 6.8, 3.7 Hz, 1H), 0.74–0.53 (m, 4H). ^13^C NMR (101 MHz, DMSO-*d*_6_) δ: 129.7, 128.3, 113.5, 97.6, 66.1, 54.1, 33.6, 31.4, 23.7, 7.2. ^19^F NMR (376 MHz, DMSO-*d*_6_) δ: −130.95 to −131.32 (m, 2F), −153.44 (s, 1F). LRMS (ESI-) *m*/*z* calcd for [C_17_H_13_F_3_NO_4_S]^−^: 384.05, found: 383.98.

#### 4-(((*N*-Cyclopropyl-2,4,6-trifluorophenyl)sulfonamido)methyl)benzoic Acid (**S5d**)

The product was obtained using synthetic procedure (c) as an amorphous solid in a quantitative yield. ^1^H NMR (400 MHz, THF-*d*_8_) δ: 8.03 (d, *J* = 7.9 Hz, 2H), 7.52 (d, *J* = 7.9 Hz, 2H), 7.17 (t, *J* = 9.4 Hz, 2H), 4.63 (s, 2H), 2.20 (p, *J* = 7.6 Hz, 1H), 0.94–0.47 (m, 2H). ^13^C NMR (101 MHz, CD_3_CN) δ: 169.2, 142.4, 140.7, 140.1, 139.8, 130.1, 110.0, 60.6, 30.5, 6.2. ^19^F NMR (376 MHz, THF-*d*_8_) δ: −100.67 (s, 1F), −102.55 (s, 2F). LRMS (ESI-) *m*/*z* calcd for [C_17_H_13_F_3_NO_4_S]^−^: 384.05, found: 384.21.

#### 4-(((3,5-Dichloro-*N*-cyclopropyl-2,4,6-trifluorophenyl)sulfonamido)methyl)benzoic Acid (**S5f**)

The product was obtained using synthetic procedure (c) as an amorphous solid. **S5f** and byproduct **S5f’** were isolated by reverse phase chromatography water/ACN (100:0 → 0:100) as amorphous solids in a quantitative yield, in a 75:25 proportion, respectively. ^1^H NMR (400 MHz, methanol-*d*_4_) δ: 8.05 (d, *J* = 8.3 Hz, 2H), 7.54 (d, *J* = 8.4 Hz, 2H), 4.69 (s, 2H), 2.55 (p, *J* = 6.7, 4.9, 3.6 Hz, 1H), 0.80–0.66 (m, 4H). ^13^C NMR (101 MHz, CD_3_CN) δ: 170.0, 141.1, 140.5, 140.1, 133.2, 132.1, 130.0, 130.1, 111.5, 70.9, 31.3, 7.0. ^19^F NMR (376 MHz, methanol-*d*_4_) δ: −105.06 (s, 1F), −107.80 (s, 2F). LRMS (ESI+) *m*/*z* calcd for [C_16_H_11_BrF_5_NO_2_SNa]^+^: 454.96, found: 454.31.

#### 4-(((4-Chloro-*N*-cyclopropyl-2,3,5,6-tetrafluorophenyl)sulfonamido)methyl)benzoic Acid (**S5g**)

The product was obtained using synthetic procedure (c) as an amorphous solid in a quantitative yield. ^1^H NMR (400 MHz, CDCl_3_) δ: 8.10 (d, *J* = 7.9 Hz, 2H), 7.50 (d, *J* = 7.9 Hz, 2H), 4.64 (s, 2H), 2.48–2.32 (m, 1H) 0.79–0.63 (m, 4H). ^13^C NMR (101 MHz, CDCl_3_) δ: 161.2, 150.9, 150.8, 145.6, 145.2, 107.7, 107.4, 106.1, 67.5, 29.0, 23.7. ^19^F NMR (376 MHz, CDCl_3_) δ: −135.11 to −135.34 (m, 2F), −137.65 to −137.84 (m, 2F). LRMS (ESI-) *m*/*z* calcd for [C_17_H_11_ClF_4_NO_4_S]^−^: 436.00, found: 436.31.

#### *N*-(Benzyloxy)-4-(((*N*-cyclopropyl-2,3,4,5,6-pentafluorophenyl)sulfonamido)methyl)benzamide (**S6a**)

The product was obtained using synthetic procedure (f) as an amorphous solid (62%). ^1^H NMR (400 MHz, CDCl_3_) δ: 9.11 (s, 1H), 7.68 (d, *J* = 8.2 Hz, 2H), 7.52–7.31 (m, 7H), 5.03 (s, 2H), 4.57 (s, 2H), 2.37 (p, *J* = 5.5, 4.8 Hz, 1H), 0.73–0.64 (m, 4H). ^13^C NMR (101 MHz, CDCl_3_) δ: 163.4, 155.9, 154.3, 145.6, 140.4, 135.2, 131.6, 129.3, 128.8, 128.6, 128.4, 127.5, 78.4, 54.1, 30.4, 6.9. ^19^F NMR (376 MHz, CDCl_3_) δ: −134.90 (s, 2F), −145.01 to −145.29 (m, 1F), −158.00 to −158.30 (m, 2F). LRMS (ESI+) *m*/*z* calcd for [C_24_H_19_F_5_N_2_O_4_SNa]^+^: 549.09, found: 549.43.

#### 4-(((3,5-Dichloro-*N*-cyclopropyl-2,4,6-trifluorophenyl)sulfonamido)methyl)-*N*-((tetrahydro-2*H*-pyran-2-yl)oxy)benzamide (**S6b**)

The product was obtained using synthetic procedure (f) as an amorphous solid (88%). ^1^H NMR (400 MHz, CDCl_3_) δ: 9.24 (s, 1H), 7.76 (d, *J* = 8.1 Hz, 2H), 7.61 (m, 1H), 7.43 (d, *J* = 8.0 Hz, 2H), 5.08 (s, 1H), 4.56 (s, 2H), 4.03 (dd, *J* = 10.4, 9.1, 2.9 Hz, 1H), 3.70–3.61 (m, 1H), 1.99–1.75 (m, *J* = 11.0, 9.8, 5.3 Hz, 3H), 1.75–1.47 (m, *J* = 14.5, 10.6, 5.1 Hz, 4H), 1.33–1.17 (m, 1H), 0.69–0.49 (m, 4H). ^13^C NMR (101 MHz, CDCl_3_) δ: 140.7, 131.6, 128.5, 127.6, 112.7, 102.7, 68.5, 62.6, 54.0, 30.3, 28.0, 27.7, 25.0, 22.1, 18.6, 7.0. ^19^F NMR (376 MHz, CDCl_3_) δ: −133.36 to −133.98 (m, 1F), −135.31 (s, 1F), −145.79 to −146.76 (m, 1F), −150.49 to −151.20 (m, 1F). LRMS (ESI) *mass not observed*.

#### *N*-(Benzyloxy)-4-(((*N*-cyclopropyl-3,4,5-trifluorophenyl)sulfonamido)methyl)benzamide (**S6c**)

The product was obtained using synthetic procedure (f) as an amorphous solid (71%). ^1^H NMR (400 MHz, CD_3_CN) δ: 9.99 (s, 1H), 7.76–7.67 (m, 2H), 7.67–7.57 (m, 2H), 7.53–7.46 (m, 2H), 7.47–7.36 (m, 5H), 4.99 (s, 2H), 4.43 (s, 2H), 2.26–2.18 (m, 1H), 0.72–0.64 (m, 4H). ^13^C NMR (101 MHz, CD_3_CN) δ: 164.9, 152.2, 149.7, 144.0, 141.1, 136.0, 134.4, 131.7, 129.2, 128.5, 128.4, 127.2, 114.6, 77.7, 54.1, 31.1, 6.8. ^19^F NMR (376 MHz, CD_3_CN) δ: −132.13 to −132.99 (m, 2F), −154.83 (s, 1F). LRMS (ESI-) *m*/*z* calcd for [C_24_H_21_F_3_N_2_O_4_SCl]^−^: 525.09, found: 525.19.

#### *N*-(Benzyloxy)-4-(((*N*-cyclopropyl-2,4,6-trifluorophenyl)sulfonamido)methyl)benzamide (**S6d**)

The product was obtained using synthetic procedure (f) as an off-white solid (61%). ^1^H NMR (400 MHz, CD_3_CN) δ: 10.01 (s, 1H), 7.80–7.65 (m, 2H), 7.57–7.34 (m,7H), 7.12–6.97 (m, 2H), 4.99 (s, 2H), 4.59 (s, 2H), 2.27–2.16 (m, 1H), 0.72–0.58 (m, 4H). ^13^C NMR (101 MHz, CD_3_CN) δ: 178.0, 154.3, 151.0, 150.4, 141.4, 135.9, 131.7, 129.2, 128.5, 128.4, 127.3, 117.3, 77.8, 53.5, 30.2, 6.3. ^19^F NMR (376 MHz, CD_3_CN) δ: −100.83 to −101.19 (m, 1F), −103.33 to −103.70 (m, 2F). LRMS (ESI-) *m*/*z* calcd for [C_24_H_21_F_3_N_2_O_4_SCl]^−^: 525.09, found: 525.33.

#### *N*-(Benzyloxy)-4-(((*N*-cyclopropyl-4-fluorophenyl)sulfonamido)methyl)benzamide (**S6e**)

The product was obtained using synthetic procedure (f) as a yellow solid (67%). ^1^H NMR (400 MHz, CD_3_CN) δ: 7.96–7.83 (m, 2H), 7.71–7.63 (m, 2H), 7.52–7.46 (m, 2H), 7.45–7.29 (m, 7H), 4.40 (s, 2H), 2.17–2.05 (m, 1H), 0.67–0.57 (m, 4H). ^13^C NMR (101 MHz, CD_3_CN) δ: 169.9, 152.3, 151.9, 151.8, 151.5, 150.9, 142.2, 136.4, 131.0, 129.0, 128.3, 112.5, 112.3, 77.8, 53.0, 23.3, 21.5, 13.9, 6.0. ^19^F NMR (376 MHz, acetone-*d*_6_) δ: −107.10 to −107.39 (m, 1F). LRMS (ESI-) *m*/*z* calcd for [C_24_H_23_FN_2_O_4_SCl]^−^: 489.11, found: 489.35.

#### 4-(((3,5-Dichloro-*N*-cyclopropyl-2,4,6-trifluorophenyl)sulfonamido)methyl)-*N*-((tetrahydro-2*H*-pyran-2-yl)oxy)benzamide (**S6f**)

The product was obtained using synthetic procedure (f) as an amorphous solid (82%). ^1^H NMR (400 MHz, CDCl_3_) δ: 9.14 (s, 1H), 7.76 (d, *J* = 8.1 Hz, 2H), 7.45 (d, *J* = 8.0 Hz, 2H), 5.10 (s, 1H), 4.60 (s, 2H), 4.12–3.91 (m, 1H), 3.71–3.62 (m, 1H), 1.97–1.82 (m, 3H), 1.74–1.56 (m, 3H), 1.33–1.23 (m, 1H), 0.69 (d, *J* = 5.3 Hz, 4H). ^13^C NMR (101 MHz, CDCl_3_) δ: 156.4, 145.4, 143.2, 140.5, 131.7, 128.5, 127.6, 102.7, 62.6, 54.2, 30.4, 28.0, 25.0, 18.6, 7.0. ^19^F NMR (376 MHz, CDCl_3_) δ: −101.73 (s, 1F), −105.97 to −106.12 (m, 2F). LRMS (ESI) *mass not observed*.

#### 4-(((3-Chloro-*N*-cyclopropyl-2,4,6-trifluorophenyl)sulfonamido)methyl)-*N*-((tetrahydro-2*H*-pyran-2-yl)oxy)benzamide (**S6f’**)

The product was obtained using synthetic procedure (f) as an amorphous solid (73%). ^1^H NMR (400 MHz, CDCl_3_) δ: 9.12 (s, 1H), 7.76 (d, *J* = 8.2 Hz, 2H), 7.45 (d, *J* = 8.0 Hz, 2H), 6.96 (m, 1H), 5.09 (s, 1H), 4.59 (s, 2H), 4.12–3.95 (m, 1H), 3.73–3.57 (m, 1H), 2.36 (p, *J* = 5.3 Hz, 1H), 1.99–1.74 (m, 4H), 1.74–1.47 (m, 4H), 0.75–0.58 (m, *J* = 5.3 Hz, 4H). ^13^C NMR (101 MHz, CDCl_3_) δ: 165.7, 145.1, 144.2, 144.0, 140.8, 131.6, 127.6, 102.7, 62.6, 54.1, 30.3, 28.0, 25.0, 18.6, 6.9. ^19^F NMR (376 MHz, CDCl_3_) δ: −101.01 (s, 1F), −103.18 to −104.54 (m, 1F), −104.00 (t, *J* = 9.9 Hz, 1F). LRMS (ESI) *mass not observed*.

#### 4-(((Perfluorophenyl)sulfonamido)methyl)-*N*-((tetrahydro-2*H*-pyran-2-yl)oxy)benzamide (**S6l**)

The product was obtained using synthetic procedure (f) as an off-white solid (70%). ^1^H NMR (400 MHz, acetone-*d*_6_) δ 7.77 (d, *J* = 8.3 Hz, 2H), 7.44 (d, *J* = 8.2 Hz, 2H), 5.08 (s, 1H), 4.46 (s, 2H), 2.13–1.28 (m, 8H). ^13^C NMR (101 MHz, acetone) δ 205.5, 154.2, 145.7, 140.3, 139.1, 132.0, 127.9, 102.0, 62.3, 47.9, 28.0, 25.4, 25.3, 25.0, 19.9, 18.4. ^19^F NMR (376 MHz, acetone-*d*_6_) δ −138.08 to −139.04 (m, 2F), −150.03 (s, 1F), −161.43 to −162.65 (m, 2F). LRMS (ESI-) *m*/*z* calcd for [C_19_H_16_F_5_N_2_O5_S_]^−^: 479.07, found: 479.38.

#### Benzyl 4-(Aminomethyl)benzoate Hydrochloride (**S2c**)

The product was obtained using synthetic procedure (i) as a white solid (83%). ^1^H δ/ppm (400 MHz, DMSO-d6) 4.10 (s, 2H), 5.36 (s, 2H), 7.34–7.48 (m, 5H), 7.66 (d, *J* = 8.2 Hz, 2H), 8.02 (d, *J* = 8.2 Hz, 2H); ^13^C δ/ppm (100 MHz, DMSO-*d6*) 41.7, 66.3, 127.9, 128.1, 128.5, 129.2, 129.4, 129.5, 136.1, 139.6, 165.2; LRMS (ESI+) *m*/*z* calcd for [C_15_H_15_NO_2_Na]^+^: 264.10, found: 264.08.

#### Benzyl 4-(((Perfluorophenyl)sulfonamido)methyl)benzoate (**S4i**)

The product was obtained using synthetic procedure (b) as a light yellow solid (69%). ^1^H δ/ppm (400 MHz, CDCl_3_) 4.67 (d, *J* = 5.9 Hz, 2H), 5.34 (s, 2H), 6.51 (s, 1H), 7.33–7.45 (m, 7H), 8.03 (d, *J* = 8.3 Hz, 2H); ^13^C δ/ppm (100 MHz, CDCl_3_) 44.0, 67.0, 117.4, 127.6, 128.3, 128.5, 128.8, 129.9, 130.4, 136.0, 138.9, 142.2, 143.2, 145.6, 157.6, 166.2; ^19^F δ/ppm (376 MHz, CDCl_3_) −159.6 to −160.0 (m, 2F), - 150.1 (s, 1F), −140.2 to −140.4 (m, 2F); LRMS (ESI+) *m*/*z* calcd for [C_22_H_14_F_5_NO_3_Na]^+^: 458.08, found: 458.41.

#### *tert*-Butyl 4-(((2,3,4,5,6-pentafluoro-*N*-isopropylphenyl)sulfonamido)methyl)benzoate (**S11a**)

The product was obtained using synthetic procedure (d) as a viscous pale yellow oil (27%). ^1^H NMR (400 MHz, acetonitrile-*d*_3_) δ 7.92 (d, *J* = 8.4 Hz, 2H), 7.48 (d, *J* = 8.4 Hz, 2H), 4.60 (s, 2H), 4.33 (p, *J* = 6.8 Hz, 1H), 1.60 (s, 9H), 1.11 (d, *J* = 6.8 Hz, 6H). ^13^C NMR (101 MHz, CDCl_3_) δ: 165.50, 141.43, 135.97, 130.10, 129.93, 128.64, 128.31, 128.29, 127.60, 65.01, 54.25, 32.24, 7.07. ^13^C NMR (101 MHz, CDCl_3_) δ 165.5, 147.4, 147.3, 145.8, 145.0, 138.8, 133.6, 133.5, 112.0, 112., 39.71, 39.6, 23.6, 17.6, 12.3. ^19^F NMR (376 MHz, acetonitrile-*d*_3_) δ −137.37 to −137.57 (m, 2F), −149.44 (s, 1F), −161.26 to −161.46 (m, 2F). LRMS (ESI-) *m*/*z* calcd for [C_21_H_22_ClF_5_NO_4_S]^+^: 514.09, found: 514.40.

#### 4-(((2,3,4,5,6-Pentafluoro-*N*-isopropylphenyl)sulfonamido)methyl)benzoic Acid (**S5j**)

The product was obtained using synthetic procedure (e) as an off-white solid in a quantitative yield. ^1^H NMR (400 MHz, acetonitrile-*d*_3_) δ 7.98 (d, *J* = 8.3 Hz, 2H), 7.52 (d, *J* = 8.3 Hz, 2H), 4.62 (s, 2H), 4.32 (p, *J* = 6.8 Hz, 1H), 1.10 (d, *J* = 6.8 Hz, 6H). ^13^C NMR (101 MHz, CDCl_3_) δ: 165.5, 141.4, 135.9, 135.7, 129.0, 128.9, 128.3, 127.9, 124.1, 72.0, 50.5, 20.3. ^19^F NMR (376 MHz, acetonitrile-*d*_3_) δ −137.37 to −137.57 (m, 2F), −149.44 (s, 1F), −161.26 to −161.46 (m, 2F). LRMS (ESI) *mass not observed*.

#### *N*-Cyclopropyl-2,3,5,6-tetrafluorobenzenesulfonamide (**S9d**)

The product was obtained using synthetic procedure (b) as a yellow oil without further purification (68%). ^1^H NMR (400 MHz, chloroform-*d*) 7.39–7.28 (m, 1H), 2.47–2.35 (m, 1H), 0.74–0.63 (m, 4H). ^13^C NMR (101 MHz, CD_3_CN) δ: 140.3, 133.9, 120.3, 15.7, 4.9. ^19^F NMR (376 MHz, acetonitrile-*d*_3_) δ −137.67 to −138.01 (m, 2F), −137.79 to −138.07 (m, 2F). LRMS (ESI) *mass not observed*.

#### *tert*-Butyl 4-(((*N*-Cyclopropyl-2,3,5,6-tetrafluorophenyl)sulfonamido)methyl)benzoate (**S11b**)

The product was obtained using synthetic procedure (d) as a pale yellow viscous oil (77.4%). ^1^H NMR (400 MHz, chloroform-*d*) δ 7.97 (d, *J* = 8.2 Hz, 2H), 7.44 (d, *J* = 8.2 Hz, 2H), 7.39–7.28 (m, 1H), 4.61 (s, 2H), 2.44–2.33 (m, 1H), 1.60 (s, 9H), 0.74–0.63 (m, 4H). ^13^C NMR (101 MHz, CDCl_3_) δ: 166.0, 141.7, 136.0, 130.3, 130.0, 129.8, 128.6, 128.3, 128.2, 128.1, 67.7, 54.1, 30.2, 7.0. ^19^F NMR (376 MHz, chloroform-*d*) δ −135.41 (s, 2F), −135.86 to −136.09 (m, 2F). LRMS (ESI) *mass not observed*.

#### Benzyl 3-Formylbenzoate (**S1b**)

The product was obtained using synthetic procedure (i) as a light yellow oil (98%). ^1^H NMR (400 MHz, chloroform-*d*) δ 10.07 (s, 1H), 8.56 (m, 1H), 8.34 (dd, *J* = 7.7, 1.5 Hz, 1H), 8.09 (dd, *J* = 7.7, 1.5 Hz, 1H), 7.63 (m, 1H), 7.51–7.46 (m, 2H), 7.45–7.36 (m, 3H), 5.42 (s, 2H). ^13^C NMR (101 MHz, chloroform-*d*) δ 191.3, 165.3, 136.6, 135.6, 135.2, 133.1, 131.3, 131.2, 129.3, 128.7, 128.5, 128.4, 67.2. LRMS (ESI+) *m*/*z* calcd for [C_15_H_12_O_3_]^+^: 240.08, found: 241.11.

#### Benzyl 3-((Cyclopropylamino)methyl)benzoate (**S2b**)

The product was obtained using synthetic procedure (a) as a pale yellow oil (93%). ^1^H NMR (400 MHz, chloroform-*d*) δ 8.06 (s, 1H), 8.00 (dt, *J* = 7.8, 1.5 Hz, 1H), 7.55 (dt, *J* = 7.7, 1.5 Hz, 1H), 7.49 (d, 2H), 7.42 (dt, *J* = 7.7, 1.9 Hz, 3H), 7.38 (d, 1H), 5.40 (s, 2H), 3.90 (s, 2H), 2.16 (p, 1H), 0.48–0.45 (m, 2H), 0.45–0.40 (m, 2H). ^13^C NMR (101 MHz, chloroform-*d*) δ 166.5, 141.0, 136.1, 133.0, 130.2, 129.4, 128.6, 128.4, 128.3, 128.2, 128.2, 66.7, 53.3, 43.5, 30.1, 6.5. LRMS (ESI+) *m*/*z* calcd for [C_18_H_19_NO_2_]^+^: 281.36, found: 282.11.

#### Benzyl 3-(((*N*-Cyclopropyl-2,3,4,5,6-pentafluorophenyl)sulfonamido)methyl)benzoate (**S4h**)

The product was obtained using synthetic procedure (b) 78%. ^1^H NMR (400 MHz, chloroform-*d*) δ 8.07–8.02 (m, 2H), 7.67 (m, 1H), 7.50 (s, 1H), 7.48 (s, 2H), 7.45–7.40 (m, 3H), 5.40 (s, 2H), 4.62 (s, 2H), 2.40 (p, 1H), 0.74 (s, 2H), 0.73 (s, 2H). ^13^C NMR (101 MHz, chloroform-*d*) δ 166.0, 136.4, 135.9, 133.0, 130.5, 129.5, 129.3, 128.9, 128.6, 128.3, 128.2, 66.8, 54.1, 30.3, 7.1. ^19^F NMR (376 MHz, chloroform-*d*) δ −134.65 (dq, *J* = 21.0, 6.9, 6.0 Hz, 2F), −143.09 to −149.37 (m, 1F), −156.12 to −161.92 (m, 2F). LRMS (ESI+) *m*/*z* calcd for [C_24_H_18_F_5_NO_4_S]^+^: 511.09, found: 512.11.

#### 3-(((*N*-Cyclopropyl-2,3,4,5,6-pentafluorophenyl)sulfonamido)methyl)benzoic Acid (**S5h**)

The product was obtained using synthetic procedure (c) as an amorphous white solid in a quantitative yield (98%). ^1^H NMR (400 MHz, acetone-*d*_6_) δ 8.06 (s, 1H), 8.01 (d, *J* = 7.8 Hz, 1H), 7.66 (d, *J* = 7.6 Hz, 1H), 7.52 (t, *J* = 7.7 Hz, 1H), 4.70 (s, 2H), 2.54 (p, *J* = 6.9, 3.8 Hz, 1H), 0.78–0.61 (m, 4H). ^13^C NMR (101 MHz, acetone-*d*_6_) δ 167.4, 137.2, 132.4, 132.0, 129.4, 128.9, 128.6, 53.7, 30.1, 6.4. ^19^F NMR (376 MHz, acetone-*d*_6_) δ −136.80 (s, 2F), −145.37 to −152.60 (m, 1F), −161.51 to −162.30 (m, 2F). LRMS (ESI+) *m*/*z* calcd for [C_17_H_12_F_5_NO_4_S]^+^: 421.04, found: 422.21.

#### 3-(((*N*-Cyclopropyl-2,3,4,5,6-pentafluorophenyl)sulfonamido)methyl)-*N*-((tetrahydro-2*H*-pyran-2-yl)oxy)benzamide (**S6h**)

The product was obtained using synthetic procedure (f) as a clear oil (84.5%). ^1^H NMR (400 MHz, chloroform-*d*) δ 9.32 (s, 1H), 7.73 (s, 1H), 7.68 (d, *J* = 7.7 Hz, 1H), 7.56 (d, *J* = 7.8 Hz, 1H), 7.41 (t, *J* = 7.7 Hz, 1H), 5.08 (s, 1H), 4.56 (s, 2H), 1.93–1.75 (m, 4H), 1.73–1.53 (m, 4H), 0.68 (d, *J* = 5.7 Hz, 4H). ^13^C NMR (101 MHz, DMSO-*d*_6_) δ 146.0, 143.4, 139.5, 138.1, 133.1, 131.6, 129.0, 127.4, 126.8, 115.1, 101.5, 61.9, 53.7, 30.6, 28.4, 25.2, 18., 14.25, 6.9. ^19^F NMR (376 MHz, chloroform-*d*) δ −134.85 to −135.69 (m, 2F), −145.52 to −146.03 (m, 1F), −154.98 to −162.40 (m, 2F). LRMS (ESI+) *m*/*z* calcd for [C_22_H_21_F_5_N_2_O_5_S]^+^: 520.11, found: 520.54.

### Western Blotting

Briefly, MV4-11 cells were incubated with inhibitors prior to cell lysis with radioimmunoprecipitation assay (RIPA) buffer (20 mM Tris pH 7.4, 150 mM NaCl, 0.5% deoxycholate, 1% Triton X-100, and 0.1% sodium dodecyl sulfate (SDS)). Total protein content was determined through a BCA assay (ThermoFisher), resolved via a 4–20% polyacrylamide SDS gel, and transferred to a nitrocellulose membrane (Bio-Rad). The membranes were blocked with a 5% solution (skimmed milk powder in PBS-T). This was followed by incubation at 4 °C (overnight) with the following antibodies: acetylated α-tubulin mouse monoclonal (MABT868, EMD Millipore), acetylated histone H3 (Ac-Lys18, 07–354, Sigma), PARP-1 (ab227244, Abcam), apoptosis Western blot cocktail (136812, Abcam), cleaved PARP-1 (ab32561, Abcam), and HSC70 (sc-7298, Santa Cruz). Following overnight incubation, horseradish peroxidase (HRP)-conjugated goat anti-mouse IgG secondary antibody (7076, Cell Signaling) or HRP-linked anti-rabbit IgG secondary antibody (7074, Cell Signaling) was applied to the membrane in a 1:5000 dilution. The bands were visualized using clarity Western ECL substrate luminal/enhancer solution and peroxide solution. Western blotting analysis was carried out using Image lab software (Bio-Rad).^47,50,70^

### Cytotoxicity Assays

HeLa cells were grown in Dulbecco’s modified Eagle’s medium (DMEM) supplemented with 10% fetal bovine serum (FBS) (Sigma-Aldrich). MV4-11 were grown in Iscove’s modified Dulbecco’s medium (IMDM) supplemented with 10% FBS. MOLM-13 and MRC-9 cells were maintained in RPMI-1640 supplemented with 10% FBS. HeLa, MOLM-13, MRC-9, and MV4-11 were obtained from the American Type Culture Collection (ATCC, USA).

The PTCL cell lines, KHYG-1, NK-92, SNK6, DERL-2, Mac1, 1305 Myla, Hut78, SU-DHL-1 or T-ALL cell lines SUP-T11, DND-41, and MOLT4 were maintained in RPMI-1640 supplemented with 10% FBS, 0.06 g/L penicillin/0.1 g/L streptomycin (Pen/Strep, Gibco), and 2 mM l-glutamine (Gibco). Culture media of KHYG-1, NK-92, SNK6, and DERL-2 cells was additionally supplemented with 2.5 ng/mL recombinant human IL-2 (ImmunoTools GmbH, Germany). The authenticity of PTCL cell lines was confirmed by analysis of highly polymorphic short tandem repeat loci (STR) using the PowerPlex 16 HS System (Promega; performed by Microsynth AG, Switzerland).

Hut78 cells were obtained from CLS Cell Lines Service GmbH, Germany. Mac1, SU-DHL-1, HH, DERL-2, KHYG-1, NK-92, SUP-T11, and YT cell lines were obtained from the Deutsche Sammlung von Mikroorganismen and Zellkulturen GmbH (DSMZ, Germany). SNK6 and NK-YS were a generous gift from Dr. Wing C. Chan (City of Hope Medical Center, Duarte, CA, USA), and the DND-41 cell line was a generous gift from A. Thomas Look (Dana-Farber Cancer Institute, Boston, MA, USA). Myla cells were a generous gift from Dr. K. Kaltoft, Institute of Human Genetics, University of Aarhus (Aarhus, Denmark) via European Collection of Authenticated Cell Cultures (ECACC). MOLT4 cells were obtained from ATCC, USA.

NHF and pPF were grown with Cell System growth medium, supplemented with Culture Boost, whereas the U87-MG cells were grown in DMEM (and 10% FBS). HUVECs were grown in Vascular Cell Basal medium supplemented with Endothelial Cell Grow Kit-VEGF. NHF and pPF were obtained from Cell Systems, USA. U87-MG and HUVEC cell lines were obtained from ATCC, USA.

Cell lines were regularly tested for mycoplasma using the MycoAlert mycoplasma detection kit (Lonza Group AG, Switzerland). All cell lines were cultured at 37 °C in a humidified atmosphere containing 5% CO_2_. Experiments were performed within 20 passages after cell resuscitation. None of the above-mentioned cell lines are listed in the register of cell lines that are known to be misidentified through cross-contamination.

Cells were plated in 96-well flat-bottom sterile culture plates with low-evaporation lids (Costar #3997). The inhibitors and a vehicle control (0.5% DMSO) were added to the cells following 24 h. After 72 h, Cell Titer-Blue (Promega #G808A) was added to each well (20 μL), and the fluorescence was measured at 560/590 nm using a Cytation S63 spectrophotometer (BioTek) or a GloMax Discover Microplate Reader (Promega, Madison, WI, USA). GraphPad Prism 6.0 (GraphPad Software Inc.) was used to determine the IC_50_ values.

### Immunofluorescence Assay

HeLa cells were plated to subconfluency on a 96-well plate (clear bottom black from Fisher Scientific). As above, inhibitors were introduced following 24 h. The cells were washed with 1× PBS, fixed with 4% formaldehyde (Millipore Sigma), permeabilized with 1% Triton X-100 (Millipore Sigma), and blocked in 5% bovine serum albumin (BSA) (BioShop) for 1 h at room temperature. Following this treatment, the cells were incubated in a cocktail composed of acetylated α-tubulin mouse monoclonal (1:100 dilution, MD Millipore) and acetylated histone H3 (1:50 dilution, Ac-Lys18, Sigma) antibodies. Secondary antibodies anti-mouse Alexa Fluor 647 and anti-rabbit Alexa Fluor 488 were used for immunofluorescent detection. Cells were counterstained for nucleic acids using 4′,6-diamidino-2-phenylindole (DAPI) (ThermoFisher Scientific). The cells were imaged with a Cytation S63 spectrophotometer and the fluorescence intensity was determined using ImageJ (*n* = 3). Two-way ANOVA with Tukey’s multiple comparisons test were performed using GraphPad Prism 6.0 (GraphPad Software Inc.). A *p*-value ≤ 0.05 was considered significant.

### FACS Apoptosis Detection Assay

MV4-11 cells were prepared, dosed, and washed with cold 1× PBS. The resulting cell pellets were resuspended in 1× Binding Buffer (1 × 10^6^ cell/mL) from the FITC Annexin V Apoptosis Detection Kit I (BD Pharmingen). Subsequently, the dyes annexin V (5 μL) and propidium iodide (PI, 5 μL) were added to 2.5 × 10^5^ cells (250 μL). The suspension was thoroughly mixed and incubated in the dark for 15 min. Following the addition of 250 μL of 1× binding buffer, the cells were analyzed by flow cytometry within 1 h using the Cytoflex S system (Beckman Coulter).

### Maximum Tolerated Dose Studies

Acute toxicity of the lead inhibitors was studied using 4–12 week old male CD-1 mice (Charles River). A concentration of 20 mg/kg of inhibitor via oral gavage was administered to mice (*n* = 5) over 5 days. The vehicle consisted of 10% DMSO, 5% Cremophor EL, and 85% saline. Mice were monitored daily for toxicity effects including excessive changes in weight and hair as well as changes to posture and inactivity. All mouse studies were approved by the Local Animal Care Committee at the University of Toronto and Office of Laboratory Animal Welfare (OLAW). The mice were housed in Tecniplast Blueline ventilated cages with environmental enrichment and fed a Teklad 2019 diet supplemented with municipal water.

### Computational Modeling and Docking

Computational modeling and docking was performed with Schrödinger Maestro 11.9.011 software using Glide, Epik, Optimized Potentials for Liquid Simulations 3e (OPLS3e) force-field, Glide, LigPrep and the Protein Preparation Wizard. All ligand poses as well as protein structure images were generated with Maestro version11.9.011. Relevant compounds were prepared via a ligand preparation workflow (Epik to generate possible protonation states at target pH 7.0 ± 2.0) and generating tautomers. Protein structures were retrieved from the protein data bank (https://www.rcsb.org): HDAC6, 5EDU; HDAC8, 1T64. For protein model preparation, appropriate bond orders were assigned as well as including hydrogen atoms and creating zero-order bonds to any metal centers. Any of the loops and side chains that were not present in the protein structures were added using Prime. Furthermore, any water molecules that were located >5.0 Å from heteroatoms were removed. All residues with steric clashes were individually minimized (OPLS3e). Ramachandran plot analysis was used to examine the structure output. Receptor grids (10 × 10 × 10 Å^3^) were defined surrounding the cocrystallized ligands (Trichostatin A, TSA) within the HDAC active site during docking. The relevant HDACi were screened against HDAC6 and HDAC8 using standard precision (SP) docking, and the top 25 binding poses were generated. In all simulations, the ligands were flexible and the protein remained static (excluding rotatable groups). After postdocking minimizations, the top binding pose for each ligand was produced and analyzed for protein–ligand interactions using Maestro 11.9.011 and Ligand Interaction Diagrams.

### Permeability Determination by Lipid-PAMPA

A 1.8% solution (w/v) of lecithin in dodecane was added to each acceptor plate well (top), followed by application of the artificial membrane and addition of 300 μL of PBS (pH 7.4) solution to each well of the acceptor plate. Compounds were added to the donor plate and incubated at 25 °C, 60 rpm for 16 h. After incubation, aliquots of 50 μL from each acceptor well and donor plate were transferred into a 96-well plate, vortexed at 750 rpm for 100 s and centrifuged at 3220g for 20 min. Compound concentrations and effective permeability (*P*_e_) were determined by LC/MS/MS.

### Stability in Mouse Plasma

The plasma stability of the compounds was evaluated in mouse plasma provided from Sigma-Aldrich, Oakville, Canada. The main stock solutions of the compounds were prepared at 10 mM in DMSO, which was diluted to 100 μM in 50% acetonitrile (ACN)/50% water. Aliquots of 356.5 μL of plasma proteins were placed in a heating block and allowed to equilibrate at 37 °C for 5 min. Then, 3.5 μL of 100 μM compound solutions were added to each vial (final concentration of 1 μM). Three quality control (QC) samples at 100, 500, and 1000 nM were prepared in 0.5% ACN. The vial contents were transferred in 50 μL aliquots at time points of 0.5, 5, 10, 20, 30, and 60 mi to a 96-well autosampler plate containing 150 μL of protein precipitation solution. Ice-cold ACN containing internals standards (100 nM glyburide) were used as the protein precipitation solution. After centrifugation at 4 °C, 5500 rpm for 15 min, the supernatant was diluted in MQ water, and injected into the LC/MS/MS for quantitative analysis.

The mobile phase consisted of (A) 0.1% (v/v) formic acid in Milli-Q water; (B) 0.1% (v/v) formic acid in acetonitrile. Gradients were run over 15 min and proceeded as follows: A:B, 85:15, 0.0–1 min, 85:15 → 10:90, 1–7 min, 10:90, 7–9 min, 10:90 → 85:15, 9–9.5 min, 85:15, 9.5–15 min. The analytical column was a Waters T3 HSS iKey 1.7 μm (50 × 0.15 mm) column. The MS data was collected via multiple reaction monitoring in positive ion mode. All calculations were carried out using Microsoft Excel. Peak areas were determined from extracted ion chromatograms. The *in vitro* half-life (*t*_1/2_) of parent compounds were determined by regression analysis of the percent parent disappearance vs time curve.

### Glutathione Stability Assay

Compound was added to PBS and 5 mM GSH to reach a final concentration of 5 μM. Samples were incubated at 25 °C at 60 rpm, and aliquots were taken at 0, 30, 60, and 120 min and quenched with internal standard, IS (100 nM alprazolam, 200 nM caffeine, 200 nM Labetalol, and 100 nM tolbutamide). Samples were vortexed and centrifuged for 45 min at 3220*g*. Aliquots were diluted by ultrapure water, and used for LC/MS/MS analysis. Peak area ratios were determined and percent compounds remaining was calculated by the following equation:
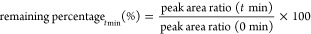
where peak area ratio (*t* min) is the peak area ratio of control and test compounds at *t* min; peak area ratio_0min_ is the peak area ratio of control and test compounds at the zero time point. *In vitro* half-life (*t*_1/2_) = −(0.693/*k*).

### Reversibility of Inhibition (*K*_off_ Kinetics)

The enzyme was preincubated with compound or DMSO, followed by dilution of the [compound/enzyme] complex into buffer with a substrate peptide to bring the compound concentration ∼10× below its respective IC_50_ value. Progress curves were obtained after dilution, and enzyme recovery was fitted with the following equation: % conv = *V*_s_ × *t* + (*V*_i_ – *V*_s_)/*K*_obs_ app × (1 – exp(−*K*_obs_ app × t), in which *V*_i_ is the initial velocity, *V*_s_ is steady state velocity, and *K*_obs_ is the observed rate constant. The obtained *K*_obs_ rate constant was used to determine the reversibility of inhibition and calculate residence time as 1/2*T*_res_ = ln2 × 1/*K*_obs_.

### Determination of *K*_i_ (*K*_on_ Kinetics)

Serial dilutions of compound were mixed with 3 nM HDAC6 enzyme and substrate peptide in assay buffer. Progress curves were acquired without preincubation for ∼5 h on a Labchip3000 instrument. Progress curves in the presence of compound were fit with the following equation: ((*A* + (*V*_s_ × *x*)) + (((*V*_o_ – *V*_s_) × (1 – exp((−1 × *K*_obs_) × t)))/*K*_obs_)). To determine observed rate of inhibition, V_o_ was locked to that in control (V_i_, DMSO only). Fractional steady state velocity (*V*_s_/*V*_i_) and *K*_obs_ values were plotted against [compound] to determine *K*_i_ and residence time.

### *In Vitro* Stability in Mouse Hepatocytes

The inhibitors (and controls) were formulated to concentrations of 100 μM in 50% acetonitrile/50% water. The medium (William’s E Medium supplemented with GlutaMAX) and hepatocyte thawing medium were allowed to reach room temperature and the hepatocytes and thawing medium and centrifuged at 100 g for 10 min. The supernatant was removed, and hepatocytes resuspended in the incubation medium (∼1.5 × 10^6^ cells/mL). The cells were diluted to 0.5 × 10^6^ viable cells/mL. A volume of 198 μL of hepatocytes was transferred to a 96-well noncoated plate and incubated at 37 °C for 10 min. Following this, 2 μL of the 100 μM test compounds or positive control was transferred into respective wells to start the reaction. At different time points (0, 15, 30, 60, 90, and 120 min), 25 μL aliquots were mixed with 6 volumes (150 μL) of acetonitrile containing internal standard, IS (100 nM alprazolam, 200 nM labetalol, 200 nM caffeine, and 200 nM diclofenac) to terminate the reaction. Samples were vortexed for 5 min and centrifuged for 45 min at 3220*g*, diluted by ultrapure water, followed by LC/MS/MS analysis. The *in vitro* half-life (*T*_1/2_) was determined by regression analysis of the percent parent disappearance vs time curve (*T*_1/2_ = 0.693/k) using Microsoft Excel. Conversion of the *in vitroT*_1/2_ into the *in vitro* intrinsic clearance (*in vitro* CL_int._ in μL/min/10^6^ cells) was performed using the following equation (mean of duplicate determinations): CL_int_ = *kV*/*N*, where *V* = incubation volume (0.2 mL) and *N* = number of hepatocytes per well (0.1 × 10^6^ cells)

### *In Vivo* PK Study in CD-1 Male Mice (Pharmaron, MA, USA)

The test compounds were formulated within a 4 mg/mL solution (10% DMA, 65% PEG400, 25% saline). CD-1 mice were administered the test compound (20 mg/kg, I.P) once, and blood samples were obtained from each mouse at 0.25, 0.5, 1, 2, 4, 8, and 24 h postdose. The working solutions of 5 μL at different concentrations (2, 4, 10, 20, 100, 200, 1000, 2000 ng/mL) were added to CD-1 mouse plasma (10 μL) to generate calibration standards of 1, 2, 5, 10, 50, 100, 500, and 1000 ng/mL. Four quality control (QC) samples at 2, 5, 50, and 800 ng/mL for plasma were prepared independently of calibration curves. Standards, QC samples, and unknown samples (total volume 15 μL) were added to acetonitrile (200 μL) containing IS (2 ng/mL verapamil, and 50 ng/mL dexamethasone) for precipitation of protein. Samples were vortexed and centrifuged (4 °C, 3900 rpm, 15 min), and the supernatant was diluted 3-fold with ultrapure water. Diluted supernatant was injected into the LC/MS/MS system for quantitative analysis.

### HDAC Target Engagement (Nanosyn, CA, USA)

The full-length human HDACs were recombinantly expressed and purified from SF9 insect cells. All compounds were serially prediluted in DMSO from a top concentration of 10 or 1 μM and introduced into the 384-well plates through an acoustic dispenser (Labcyte550) into the reaction buffer (100 mM HEPES, pH 7.5, 25 mM KCl, 0.1% BSA, 0.01% Triton X-100, and enzyme). The total concentration of DMSO was 1% in all wells. The deacetylation reaction was initiated by introduction of a FAM-labeled acetylated peptide substrate. The reaction was monitored through the change in the relative fluorescence intensity of the substrate and product peaks. The activity was determined as the product sum ratio (PSR): *P*/(*S* + *P*), where *P* = product peak height and *S* = substrate peak height. The negative control (0% inhibition in absence of inhibitor, DMSO only) and positive control (100% inhibition in absence of enzyme) were determined from *n* = 4 samples. Percent inhibition (*P*_inh_) was determined through the equation: *P*_inh_ = (PSR_0%_ – PSR_inh_)/(PSR_0%_ – PSR_100%_) × 100, where PSR_inh_ is the product sum ratio in the presence of inhibitor, PSR_0%_ is the product sum ratio in absence of inhibitor, and PSR_100%_ is the product sum ratio in 100% inhibition samples. Inhibition curves (*P*_inh_ vs inhibitor concentration) were fitted by a four-parameter sigmoid dose–response model using XLfit software (IDBS) to determine the IC_50_ values.

### Primary T-PLL Patient Sample Studies

Primary T-PLL cells were isolated from peripheral blood (PB) of 10 T-PLL patients. All patients were diagnosed according to WHO criteria based on clinical features, immunophenotyping (flow cytometry and histochemisty; including TCL1 expression), and FISH/karyotyping.^71,72^ Samples were obtained from patients under IRB-approved protocols following written informed consent according to the Declaration of Helsinki. Collection and use of patient material have been approved for research purpose by the ethics committee of the University Hospital of Cologne (EudraCT-Nr.: #2008-001421-34 and AZ11-319) and Helsinki (303/13/03/01/2011).

All patients except for one (patient 9: alemtuzumab and bendamustine) were untreated before the time of sample collection, and all the patients, except for two (Patient 8 (50%) and Patient 10 (25%)) had a > 80% T-cell fraction of their PB leukocytes. PB mononuclear cells (PBMCs) of T-PLL samples were obtained by density gradient centrifugation (Histopaque; Sigma-Aldrich). All drug screening in primary T-PLL patient samples were conducted as previously described.^34,73^

### Oncomine Gene Expression Analysis

*HDAC* gene expression data and associated statistical analyses were extracted from the Oncomine Research Premium Edition database (Thermo Fisher, Ann Arbor, MI),^46,59^ from the data sets described in Figure 7 using the following reporters: *HDAC1* (201209_at), *HDAC2* (201833_at), *HDAC3* (216326_s_at), *HDAC4* (204225_at), *HDAC5* (202455_at), *HDAC6* (206846_s_at), *HDAC9* (205659_at), and *HDAC11* (219847_at).

### Statistical Testing

Statistical analysis was performed using two-way ANOVA with Tukey’s multiple comparisons test in GraphPad Prism 6.0 (GraphPad Software Inc.). To determine differences between drug treatments in T-PLL primary samples, Wilcoxon test was performed in R with the function wilcox.test. A *p*-value of <0.05 was considered significant. *HDAC* gene expression data and associated statistical analyses were extracted from the Oncomine Research Premium Edition database (Thermo Fisher, Ann Arbor, MI) from the Durig Leukemia data set.^46,59^

## Supporting Information Available

Raw data, structures of inhibitors, additional biochemical assay results, docking results, flow cytometry, pharmacokinetic evaluations, cytotoxicity assays, and compound characterizations (PDF)
Molecular formula strings (CSV)
*In silico* docking binding pose of **5** in human HDAC6 (PDB)
*In silico* docking binding pose of **14** in human HDAC6 (PDB)
*In silico* docking binding pose of **14** in human HDAC8 (PDB)
*In vitro* IC_50_ values for **1**–**14**, SAHA, ricolinostat, and citarinostat (XLSX)
*In vitro* IC_50_ values for **14** and citarinostat (XLSX)
Pharmacology of **14** and citarinostat (XLSX)
Cellular IC_50_ values for **14** and citarinostat (XLSX)
Pharmacokinetic parameters of **14** (XLSX)

## Supplementary Material

jm1c00420_si_002.csv

jm1c00420_si_003.pdb

jm1c00420_si_004.pdb

jm1c00420_si_005.pdb

## Abbreviations Used

ABT-199: venetoclax
AcOH: acetic acid
MeCN: acetonitrile
ADME: absorption, distribution, metabolism, and excretion
AITL: angioimmunoblastic T-cell lymphoma
ALCL: Anaplastic large cell lymphoma
ALK: anaplastic lymphoma kinase
AML: acute myeloid leukemia
ATM: ataxia telangiectasia mutated
AUC: area under the curve
BCL: B-cell lymphoma
Boc: *tert*-butoxycarbonyl
FAM: carboxyfluorescein
CCLE: Cancer Cell Line Encyclopedia
CoA: coenzyme A
*C*_max_: maximum concentration the drug achieves
Cs_2_CO_3_: cesium carbonate
CTCL: cutaneous T-cell lymphomas
DCE: dichloroethane
DCM and CH_2_Cl_2_: dichloromethane
DMF: dimethylformamide
DMSO: dimethyl sulfoxide
DNA: deoxyribonucleic acid
*i*Pr_2_NEt: diisopropylethylamine
DSRT: drug sensitivity and resistance testing
DSS: drug sensitivity score, EMSA, electrophoretic mobility shift assay
FACS: fluorescence-activated cell sorting
FITC: fluorescein isothiocyanate
FDA: U.S. Food and Drug administration
GSH: reduced glutathione
*t*_1/2_: half-life
h: hour
HCl: hydrochloric acid
HPLC: high-performance liquid chromatography
HRMS: high-resolution mass spectrometry
HPOB: *N*-hydroxy-4-(2-[(2-hydroxyethyl)(phenyl)amino]-2-oxoethyl)benzamide
HSP: heat shock protein
HUVEC: normal human umbilical vein endothelial cells
HIF: hypoxia-inducible factors
IC_50_: half maximal inhibitory concentration
IS: internal standard
IP: intraperitoneal
JAK: Janus-activated kinase
LC/MS: liquid chromatography/mass spectrometry
LRMS: low-resolution mass spectrometry
MaTCL: mature T-cell leukemias and lymphomas
MeOH: methanol
min: minutes
NAD^+^: nicotinamide adenine dinucleotide
NHF: normal human fibroblasts
NK: natural killer
NMR: nuclear magnetic resonance
(COCl)_2_: oxalyl chloride
p300/CBP: EA1 binding protein 300/CREB-binding protein
Pd(OAc)_2_: palladium acetate
Pd/C: palladium on carbon
PAMPA: parallel artificial membrane permeability assay
PBMCs: peripheral blood mononuclear cells
*P*_e_: effective permeability
PFBSCl: pentafluorobenzyl sulfonyl chloride
PTCL: peripheral T-cell lymphomas
PTCL-NOS: peripheral T-cell lymphoma, not otherwise specified
PG: protecting group
PK: pharmacokinetic
P.O.: oral administration
pPF: primary pooled fibroblasts
rpm: revolutions per minute
RT: room temperature
Na(AcO)_3_BH: SCT, stem cell transplantation
sDSS: selective drug sensitivity score, sodium triacetoxyborohydride
SI: Supporting Information
T-ALL: T-cell acute lymphocytic leukemia
TC50: toxic concentration in 50% of PBMC population
TCL1A: T-cell leukemia 1A
THF: tetrahydrofuran
Et_3_N: triethylamine
SAR: structure–activity relationship
SAHA: suberanilohydroxamic acid
Sir2: sirtuin
STAT: signal transducer and activator of transcription factor
T-PLL: T-cell prolymphocytic leukemia
ZBG: zinc-binding group
