# Supplementary material for: Development of HDAC Inhibitors Exhibiting Therapeutic Potential in T-Cell Prolymphocytic Leukemia
Source: J Med Chem. 2021 Jun 8;64(12):8486–509. doi: 10.1021/acs.jmedchem.1c00420 (PMC8237267; doi:10.1021/acs.jmedchem.1c00420)
Supplement: Supplementary file 1 — jm1c00420_si_001.pdf [file jm1c00420_si_001.pdf]

Supporting Information

Development of HDAC Inhibitors Exhibiting Therapeutic Potential in T-cell Prolymphocytic Leukemia

Krimo Toutah[1,#], Nabanita Nawar[1,2#], Sanna Timonen[3,4,5], Helena Sorger[6], Yasir S. Raouf[1,2], Shazreh Bukhari[1,2], Jana von Jan[7,8,9], Aleksandr Ianevski[5], Justyna M. Gawel[1], Olasunkanmi O. Olaoye[1,2], Mulu Geletu[1], Ayah Abdeldayem[1,2], Johan Israelian[1,2], Tudor B. Radu[1,2], Abootaleb Sedighi[1], Muzaffar N. Bhatti[1], Muhammad M. Hassan[1,2], Pimyupa Manaswiyoungkul[1,2], Andrew E. Shouksmith[1], Heidi A. Neubauer[6], Elvin D. de Araujo[12], Tero Aittokallio[5,10,11], Oliver H. Krämer[13], Richard Moriggl[6*], Satu Mustjoki[3,4,14*], Marco Herling[7,8,9*], Patrick T. Gunning[1,2,12*]

[#] These authors contributed equally

[1]Department of Chemical and Physical Sciences, University of Toronto Mississauga, 3359 Mississauga Road, Mississauga, Ontario, L5L 1C6, Canada

[2]Department of Chemistry, University of Toronto, 80 St. George Street, Toronto, Ontario M5S 3H6, Canada

[3]Hematology Research Unit Helsinki, Helsinki University Hospital Comprehensive Cancer Center, Helsinki, Finland

[4]Translational Immunology Research Program and Department of Clinical Chemistry and Hematology, University of Helsinki, Helsinki, Finland

[5]Institute for Molecular Medicine Finland (FIMM), HiLIFE, University of Helsinki, Helsinki, Finland

[6]Institute of Animal Breeding and Genetics, University of Veterinary Medicine Vienna, Vienna, Austria

[7]Department of Internal Medicine, Center for Integrated Oncology Aachen-Bonn-Cologne-Duesseldorf (CIO ABCD), University of Cologne (UoC), Cologne, Germany

[8]Excellence Cluster for Cellular Stress Response and Aging-Associated Diseases (CECAD), UoC, Cologne, Germany

[9]Center for Molecular Medicine Cologne (CMMC), UoC, Cologne, Germany

[10]Department of Cancer Genetics, Institute for Cancer Research, Oslo University Hospital, Oslo, Norway

[11]Oslo Centre for Biostatistics and Epidemiology, University of Oslo, Oslo, Norway

[12]Centre for Medicinal Chemistry, University of Toronto Mississauga, 3359 Mississauga Road, Mississauga, Ontario, L5L 1C6, Canada

[13] Department of Toxicology, University Medical Center, 55131 Mainz, Germany

[14]iCAN Digital Precision Cancer Medicine Flagship, Helsinki, Finland

Table of Contents

Fig. S1. Examples of validated HDAC inhibitors in literature 2

Fig S2. In vitro IC_{50} values of 5 and 14 (KT-531) for a range of preincubation times 2

Fig S3. K_{on} experiment to determine kinetic parameters of time-dependent inhibition of compound 14 (KT-531). 3

Fig. S4. Ramachandran plot outputs of human HDAC6, HDAC8 3

Fig. S5. In silico docking binding pose of 14 (KT-531) and 13 in human HDAC6 4

Fig. S6. Flow cytometry data 4

Fig. S7. Cleavage of Caspase 3 and PARP-1 upon dose-escalation of citarinostat 4

Fig S9. In vivo PK parameters of 14 5

Table S1a. Stability of selected compounds towards glutathione (GSH). 6

Table S1b. Stability of 5 and 12 in hepatocytes. 6

Table S2a. Stability of 6 and 14 (KT-531) in mouse plasma. 6

Table S2b. In vitro and in cellulo activity of Nexturastat. 6

Table S3. K_{off} kinetic parameters 6

Table S4. Cytotoxicity of SAHA and belinostat in healthy non-cancerous cell lines 7

Table S5 Comparison of HDAC8 selective inhibitors (PCI-34051 and MMH-410) with KT-531. 7

^{1}H NMR spectra and Analytical HPLC traces 7

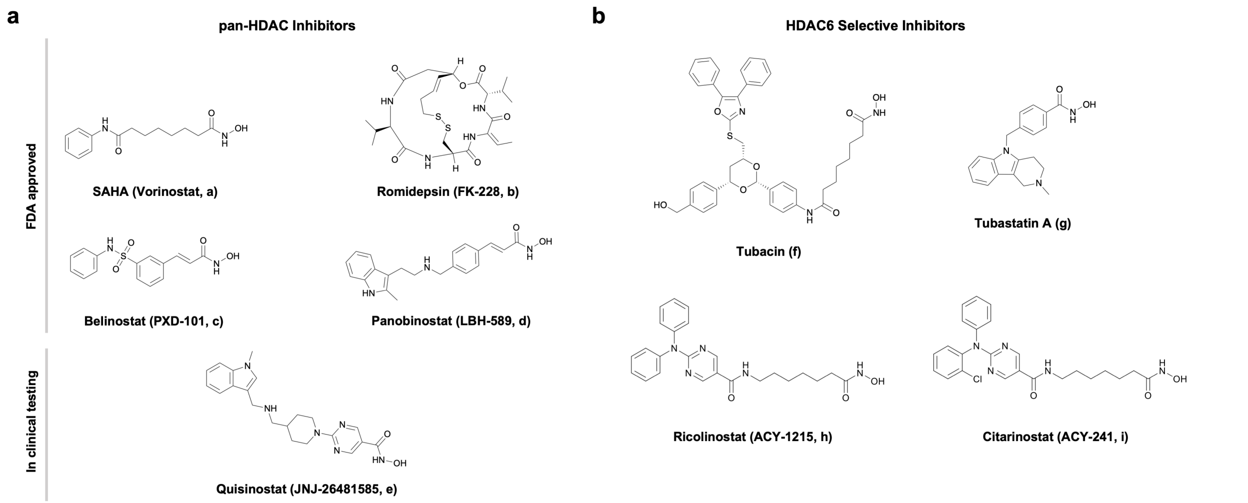

Fig. S1. Examples of validated HDAC inhibitors in literature. a Selected pan-HDAC inhibitors (a-e). a-d are FDA approved. **b** HDAC6-selective inhibitors (f-i).

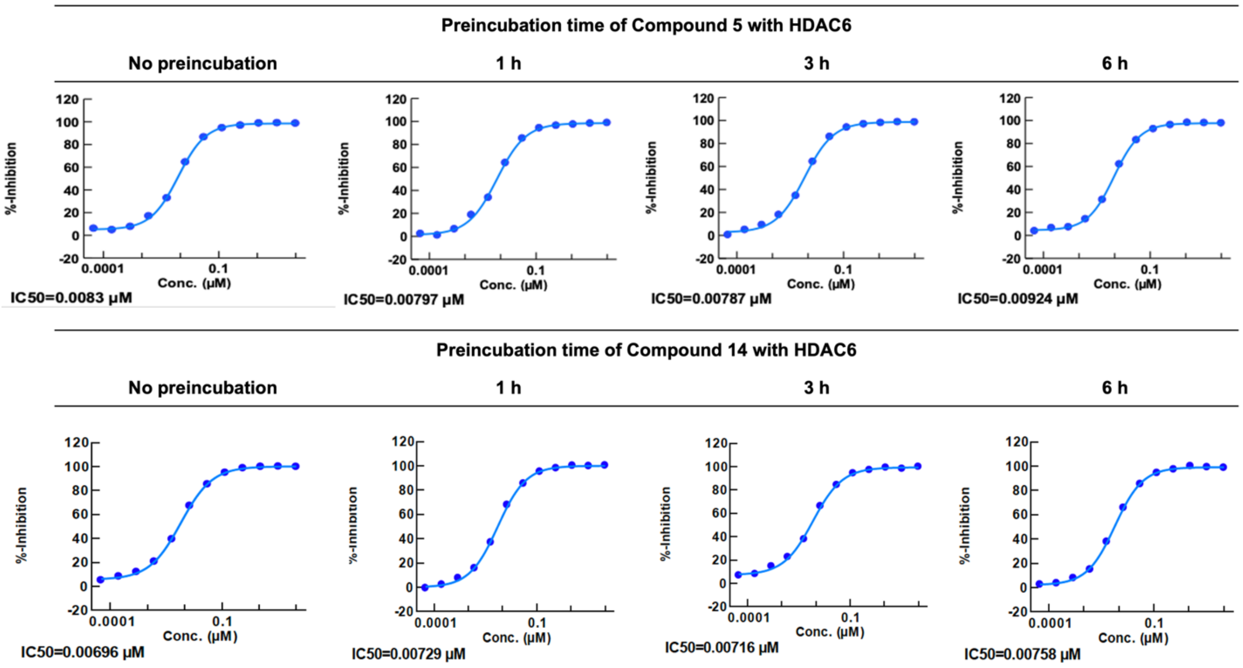

Fig S2. *In vitro* IC_{50} values of **5** and **14** (**KT-531**) for a range of preincubation times (0, 1, 3 and 6 h) with HDAC6.

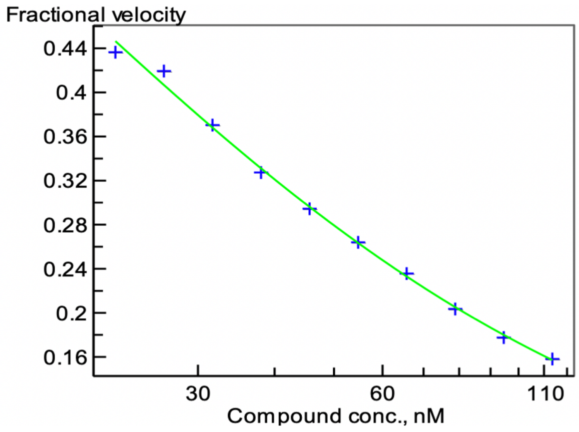

K-on kinetic parameter	Compound 14
K_i	17.29 nM
Slope (D)	-0.89

$$E + I \underset{k_{off}}{\overset{K_{on}}{\rightleftarrows}} E{:}I^*$$

Fit = (1+((Ki/[I])^D))))

Fig S3. K_{on} experiment to determine kinetic parameters of time-dependent inhibition of compound **14** (**KT-531**).

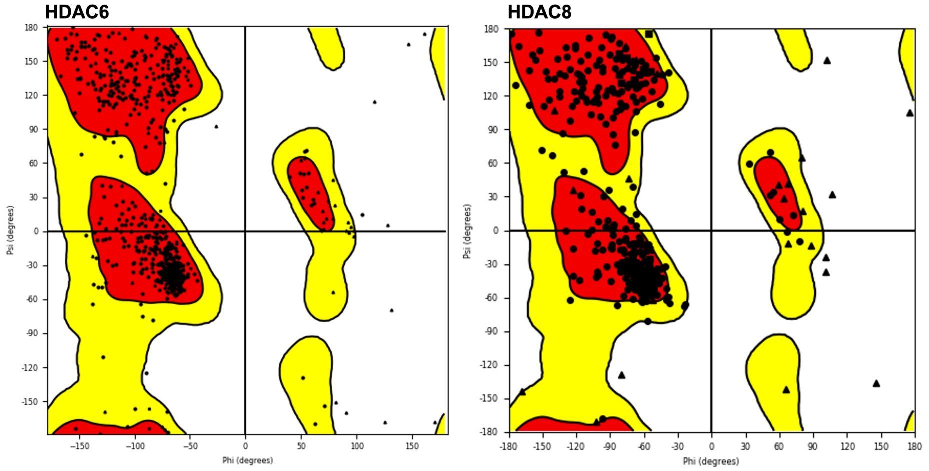

Fig. S4. Ramachandran plot outputs of human HDAC6, HDAC8 (PDB 5EDU, 1T64 respectively) post protein-preparation. As expected, due to their structural flexibility, only Gly and Ala residues were found to occupy the disfavoured region (quadrant IV, bottom right). No other sterically forbidden angles were observed.

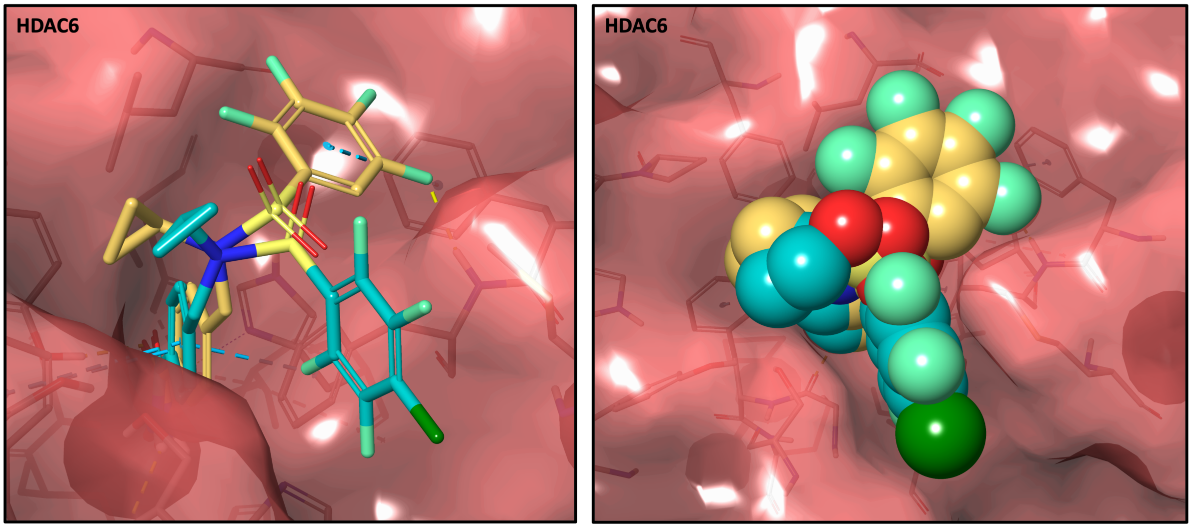

Fig. S5. *In silico* docking binding pose of **14** (**KT-531**) and **13** in human HDAC6 (PDB 5EDU), hydrogen-bonds (yellow-dashed line), π-π stacking (blue-dashed line) **KT-531** (C, yellow); **13** (C, blue-green) (N, blue; O, red; C, green; F, blue-green). The binding poses of each represent the difference in their conformation and interacting residues.

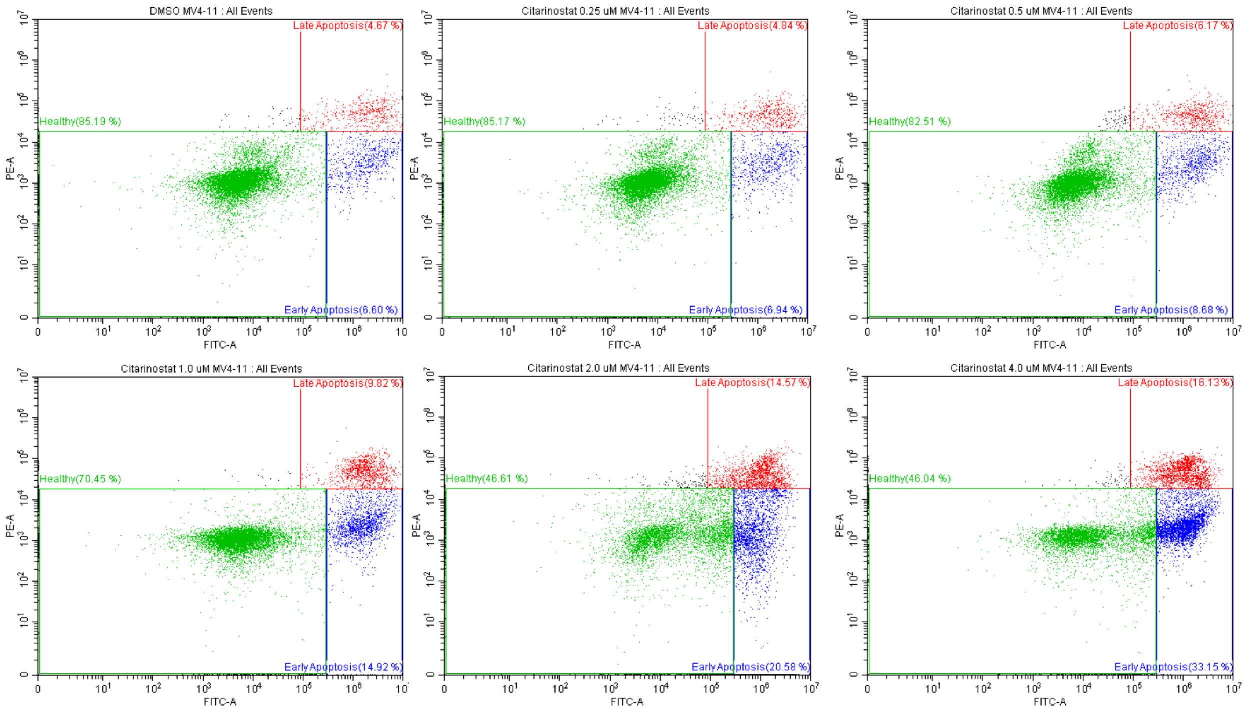

Fig. S6. Flow cytometry data of MV4-11 cells treated with increasing concentrations of citarinostat for 18 h and analyzed for apoptosis via Annexin V/PI staining. Representative dot blots from three independent experiments are presented.

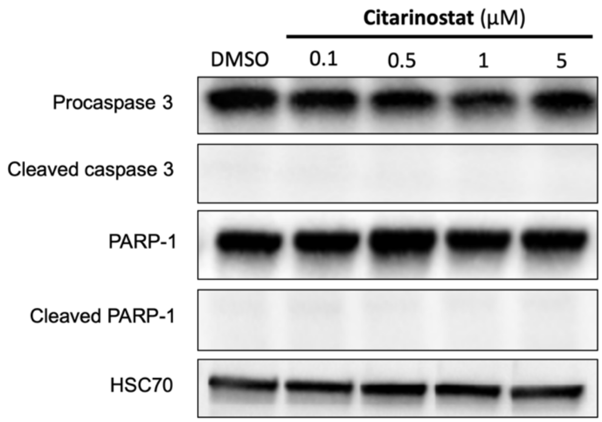

Fig. S7. Cleavage of Caspase 3 and PARP-1 upon dose-escalation of citarinostat for 6 h in MV4-11 cells. Protein extracts were prepared and subjected to SDS-PAGE and immunoblotting with Procaspase 3, cleaved Caspase-3, PARP-1, cleaved PARP-1, and HSC70 antibodies. A representative Western blot of three independent experiments is shown.

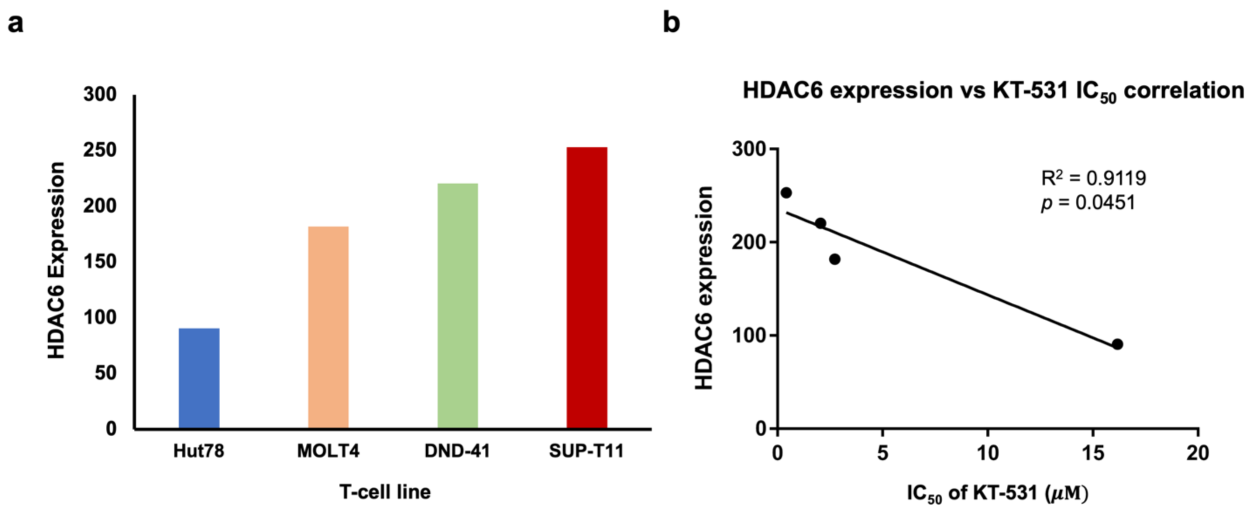

Fig. S8. a. Publicly available HDAC6 mRNA expression levels of available PTCL and T-ALL model cell lines (data from the Cancer Cell Line Encyclopedia, obtained via the Betastasis web portal). SUP-T11, a quasi-model of T-PLL showed the highest HDAC6 level. **b.** Correlation (linear regression model) of HDAC6 expression levels in cells with their sensitivity to **KT-531** (IC_{50} values).

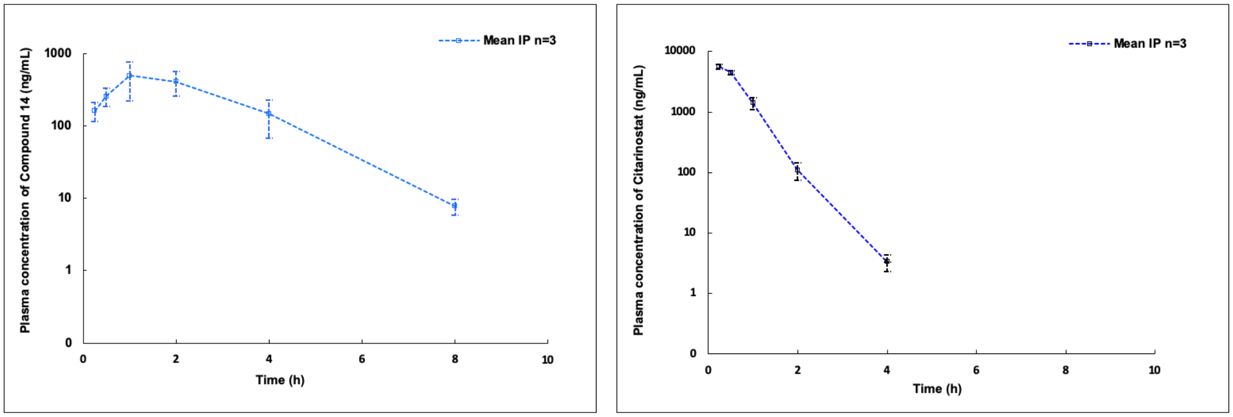

Compound	$t_{1/2}$ (h)	t_{max} (h)	C_{max} (ng/mL)	AUC_{last} (h*ng/mL)	AUC_{Inf} (h*ng/mL)	AUC Extr (%)	MRT (h)	AUC/D (h*mg/mL)
Compound 14	1.05	1.33	493	1576	1519	0.82	2.10	79
Citarinostat	0.352	0.250	5640	4331	4163	0.0365	0.5764	217

Fig S9. *In vivo* PK parameters of **14** (**KT-531**) and citarinostat in male CD-1 mice (n=3) via IP (20mg/kg).

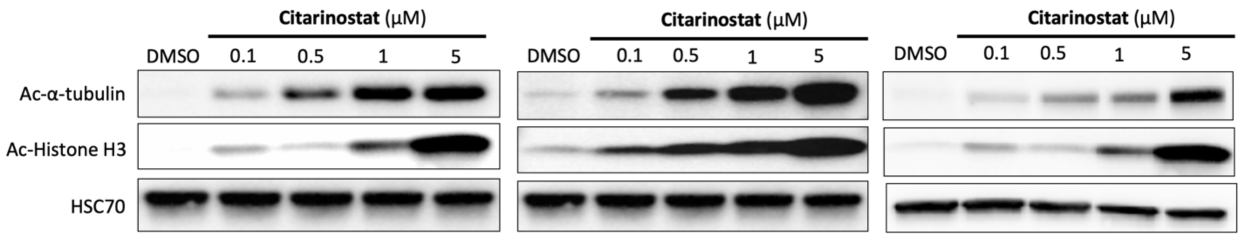

Fig. S10. Western blot illustrating α-tubulin acetylation and histone H3 acetylation levels in MV4-11 AML cells following 6 h treatment with varying concentrations of clinical candidate citarinostat. Protein extracts were prepared, resolved by SDS-PAGE and immunoblotted with acetylated α-tubulin, acetylated histone H3, and HSC70 antibodies.

Table S1a. Stability of selected compounds towards glutathione (GSH).

In vitro Stability (GSH)	
**Compound**	**$t_{1/2}$ (min)**
**3**	106.33
**4**	222.52
**6**	∞
**12**	∞
**14 (KT-531)**	∞

Table S1b. Stability of **5** and **12** in hepatocytes.

In vitro Stability in hepatocytes			
**Compound**	**Species**	**$T_{1/2}$ (min)**	**CL_{int} (μL/min/10^6 cells)**
**5**	Mouse	<2.26	>614.02
	Human	3.84 $\pm$ 0.01	361.41 $\pm$ 1.85
**12**	Mouse	5.08 $\pm$ 0.03	272.65 $\pm$ 1.92

Table S2a. Stability of **6** and **14** (**KT-531**) in mouse plasma.

Compound	Species	Remaining Percentages (%)						Half-life (min)
		0 min	15 min	30 min	45 min	60 min	120 min	
**6**	Mouse	100	67.8	32.7	44.5	19.7	3.4	25
**14**	Mouse	100	38.5	15.7	9.1	9.3	9.8	41

Table S2b. *In vitro* and *in cellulo* activity of Nexturastat.

	In vitro IC_{50} (μM)				HDAC6 fold-selectivity	*In cellulo* IC_{50} (μM)
**Compound**	**HDAC3**	**HDAC6**	**HDAC8**	**HDAC11**		**MV4-11**
**14 (KT-531)**	>1	0.00850	0.334	>1	39.29	0.42
**Nexturastat**	0.238	0.0124	>1	>1	19.19	1.68

Table S3. K_{off} kinetic parameters of **1**, **5** and **14** (**KT-531**) with HDAC6.

K-off kinetic parameter	Compound 1	Compound 5	Compound 14
**V_0 (10^{-6},% conversion/s)**	**3.66**	**0**	**5.75**
**Observed rate (s^{-1})**	**0.00156**	**0.015163**	**0.000718**
**Recovery (%)**	**89.3**	**85.7**	**112.8**
**Residence time (min)**	**10.716**	**1.0991**	**23.184**

Table S4. Cytotoxicity of SAHA and belinostat in healthy non-cancerous cell lines (Normal Human Fibroblasts (NHF) and Human Umbilical Vein Endothelial Cells (HUVEC)).

	Cellular IC_{50} (μM)	
**Compound**	**NHF**	**HUVEC**
**SAHA**	4.54	3.55
**Belinostat**	1.13	0.60

Table S5 Comparison of HDAC8 selective inhibitors (PCI-34051 and MMH-410) with **KT-531**.

	In vitro IC_{50} (μM)				*In cellulo* IC_{50} (μM)	
**Compound**	**HDAC3**	**HDAC6**	**HDAC8**	**HDAC11**	**MV4-11**	**MRC-9**
**14 (KT-531)**	>1	0.00850	0.334	>1	0.42	>20
**PCI-34051**	>1	>1	0.00403	0.482	>50	>50
**MMH-410**	>1	>1	0.0655	>1	>50	>50

^{1}H NMR spectra and Analytical HPLC traces

Compound 1

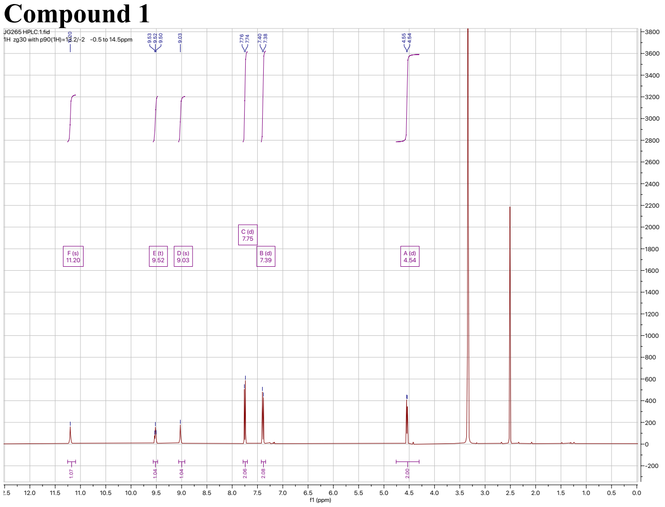

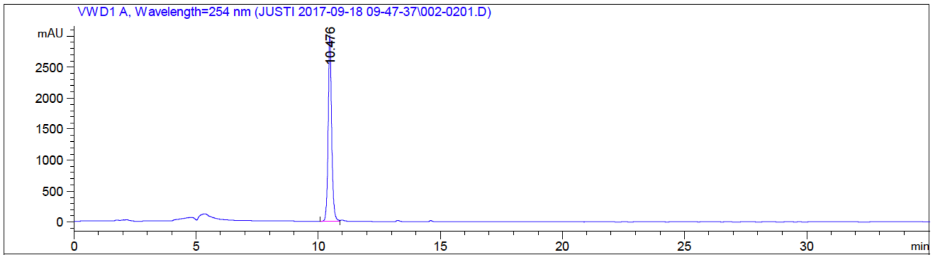

Compound 2

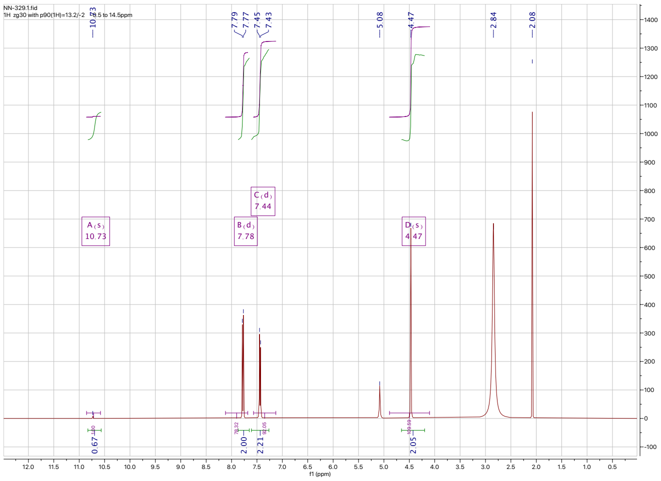

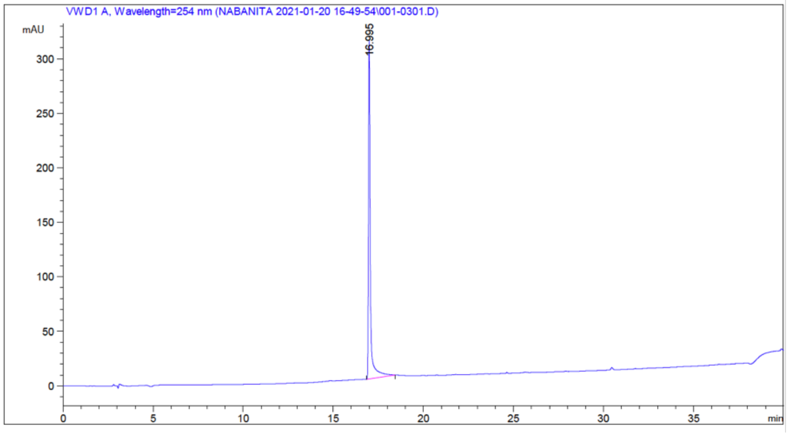

Compound 3

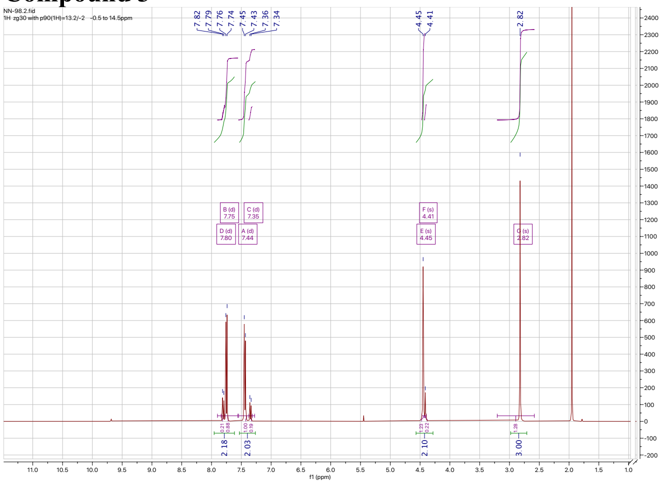

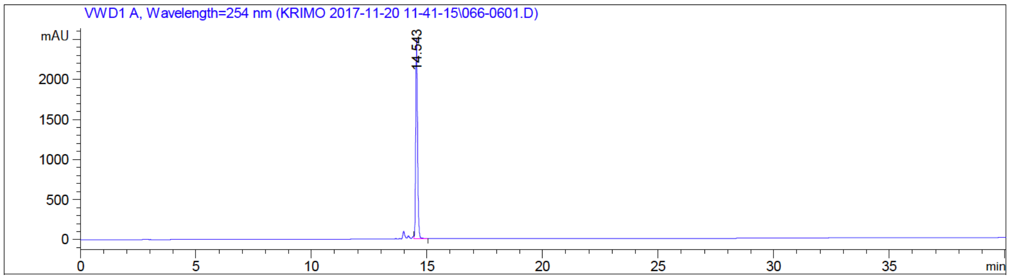

Compound 4

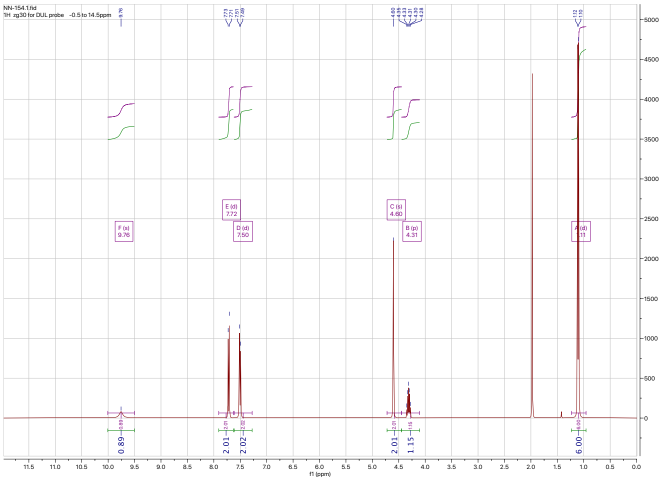

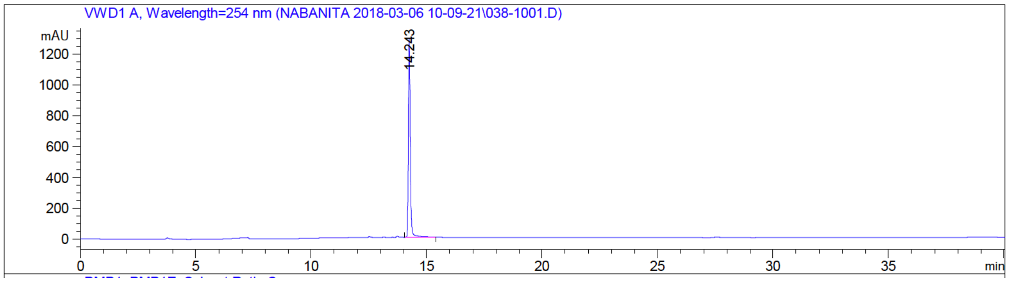

Compound 5

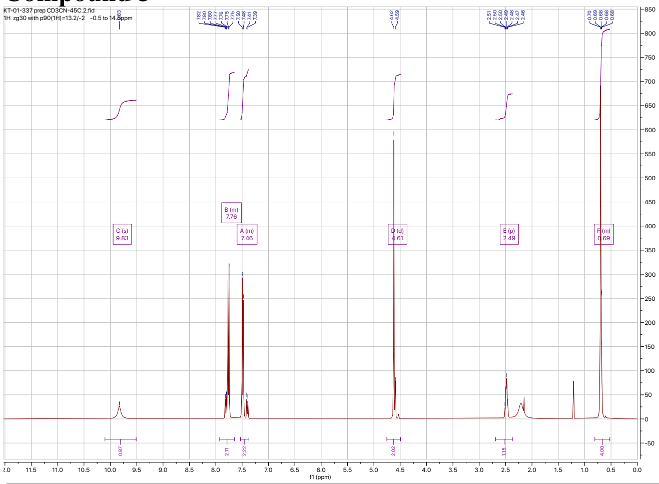

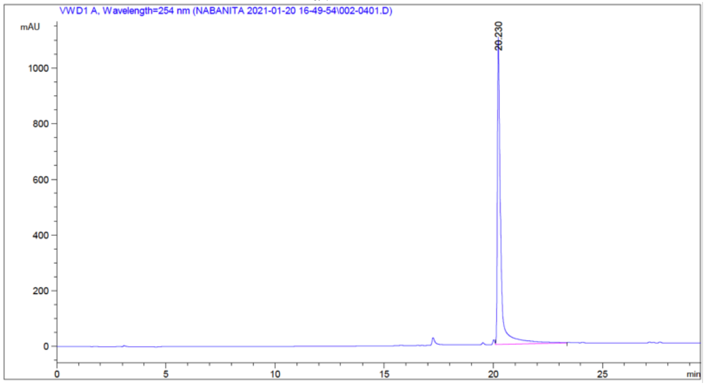

Compound 6

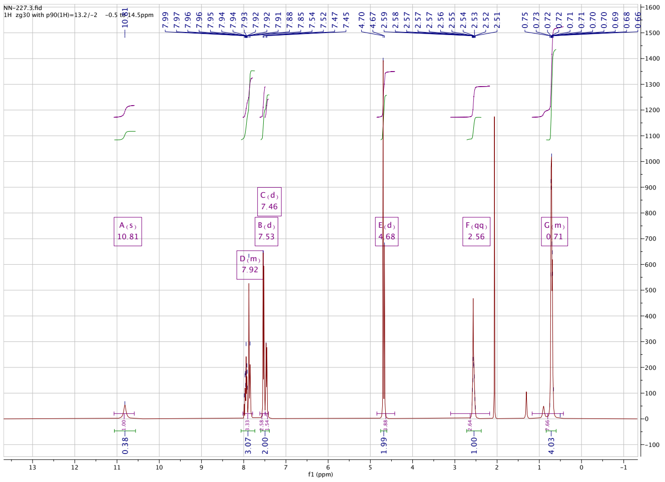

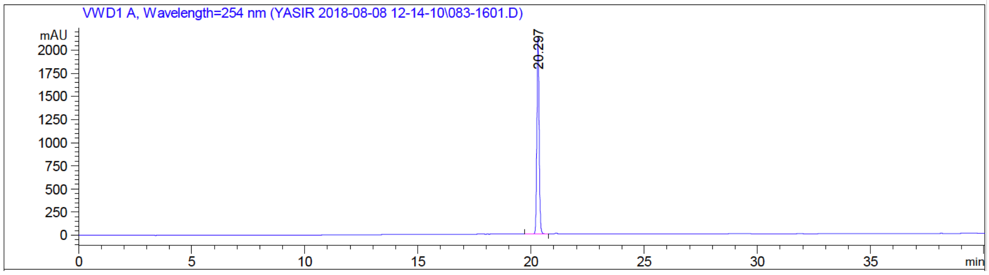

Compound 7

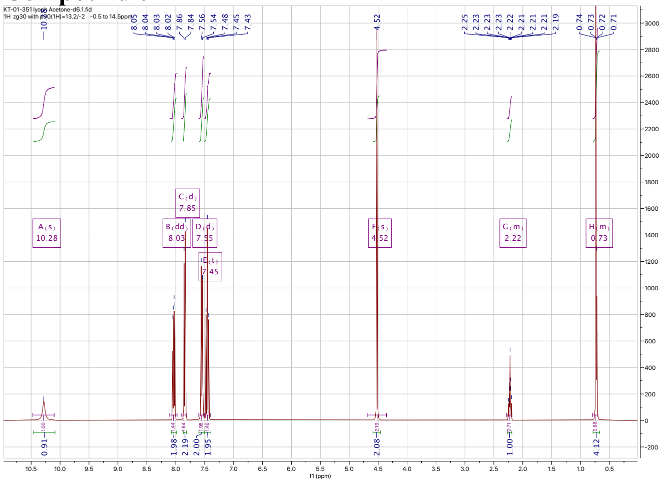

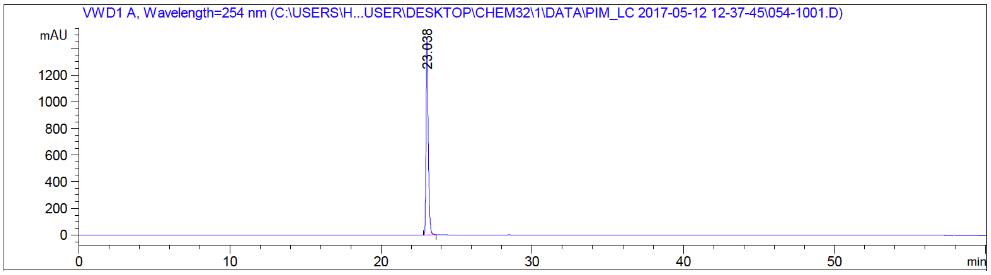

Compound 8

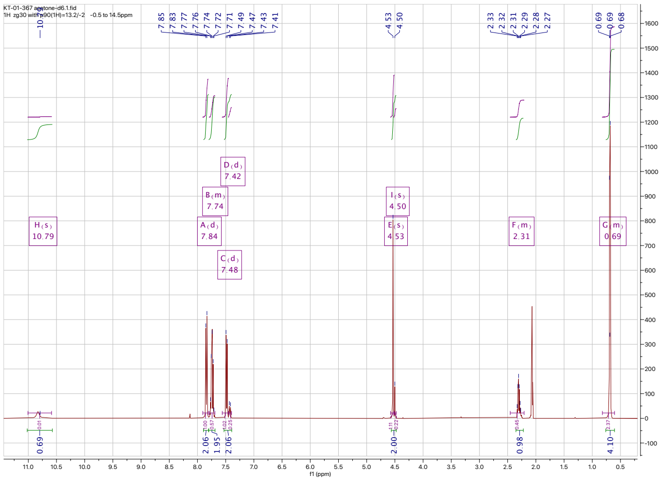

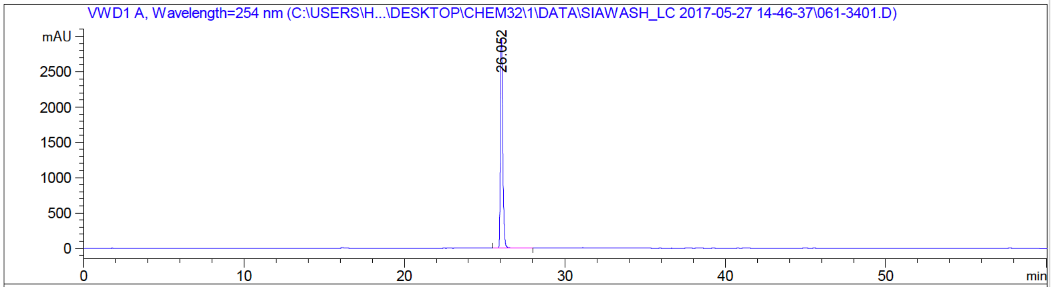

Compound 9

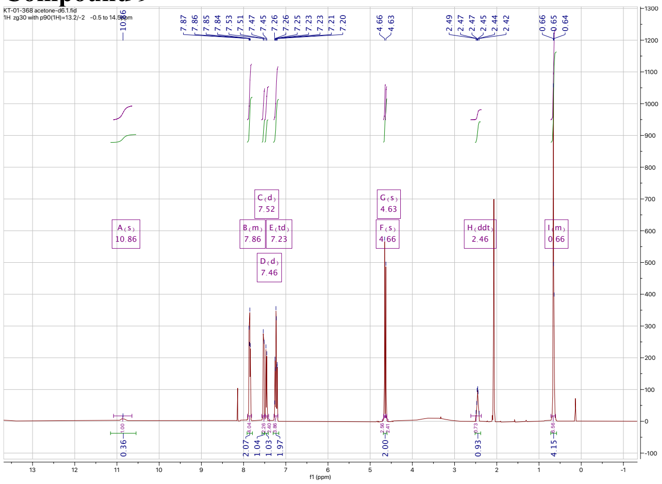

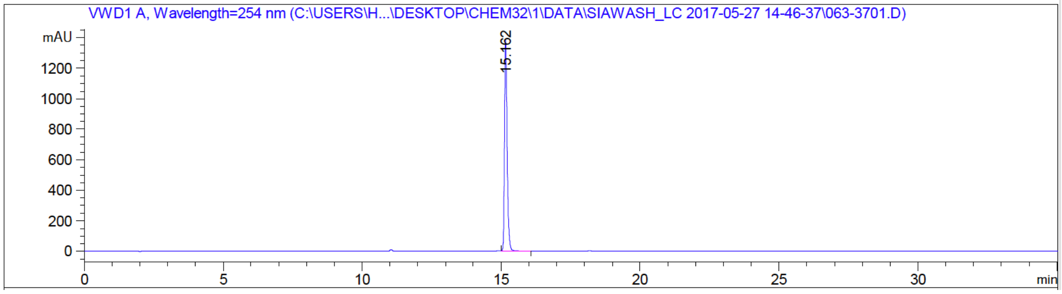

Compound 10

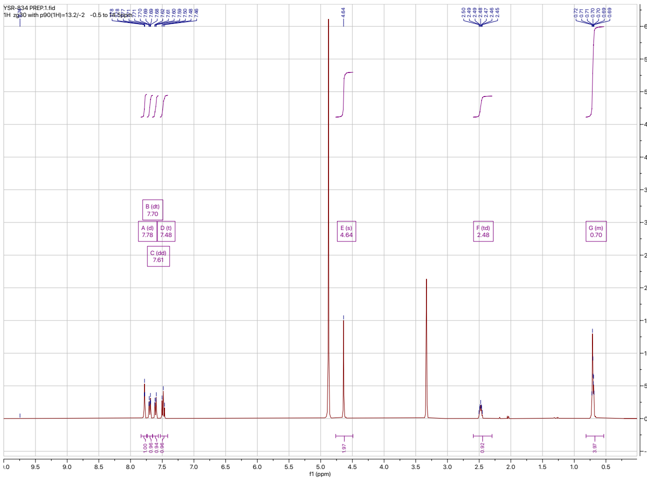

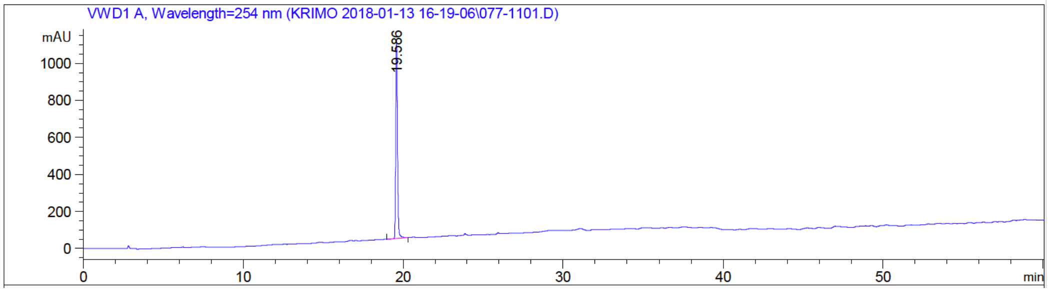

Compound 11

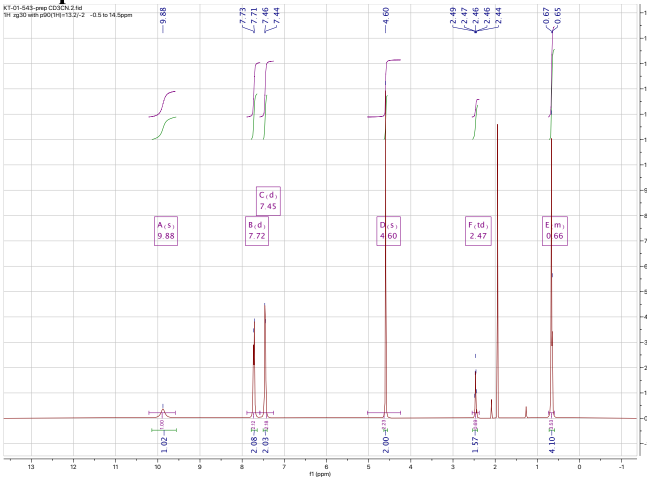

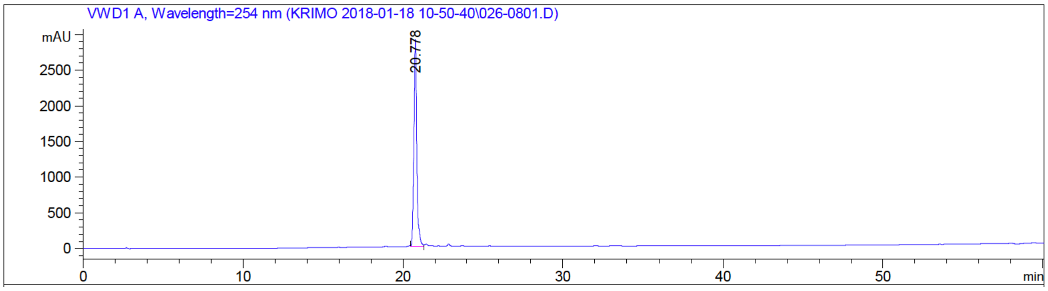

Compound 12

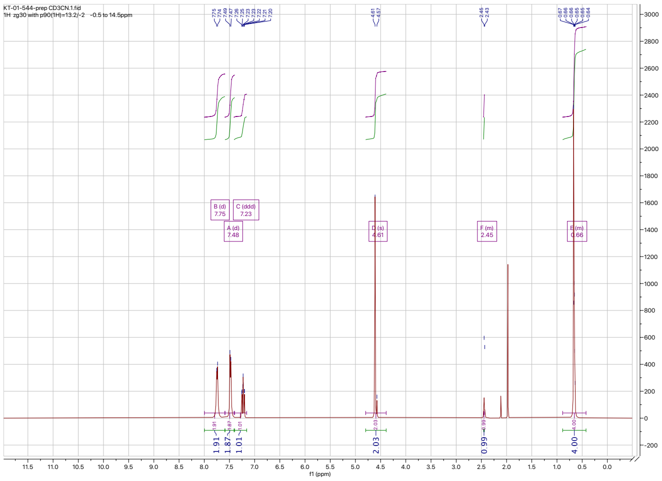

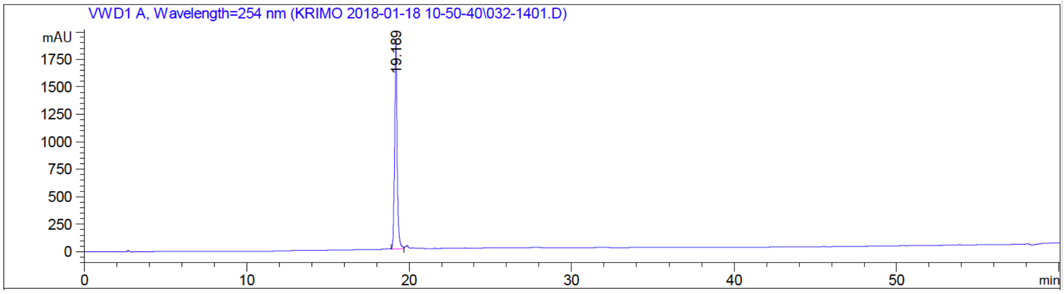

Compound 13

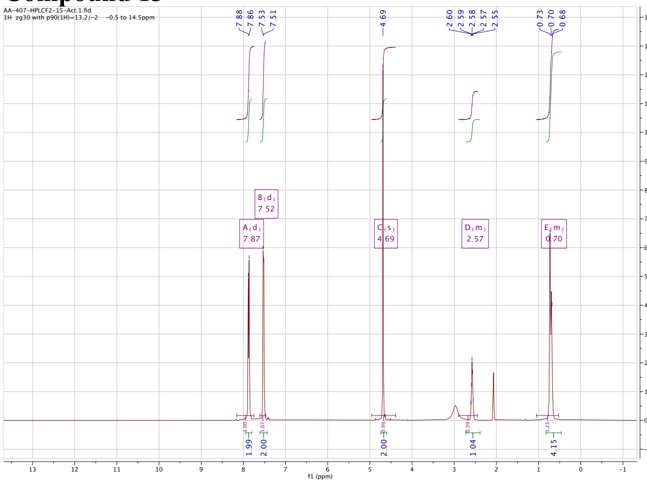

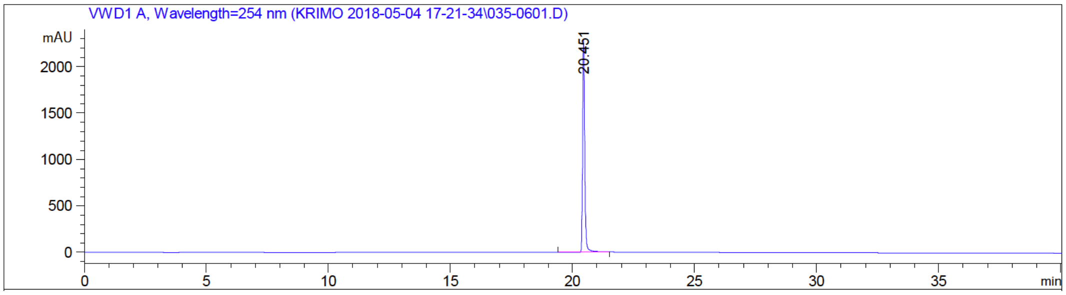

Compound 14

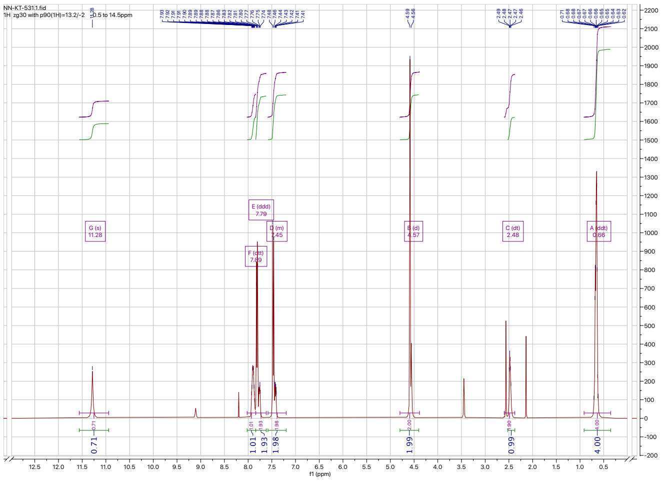

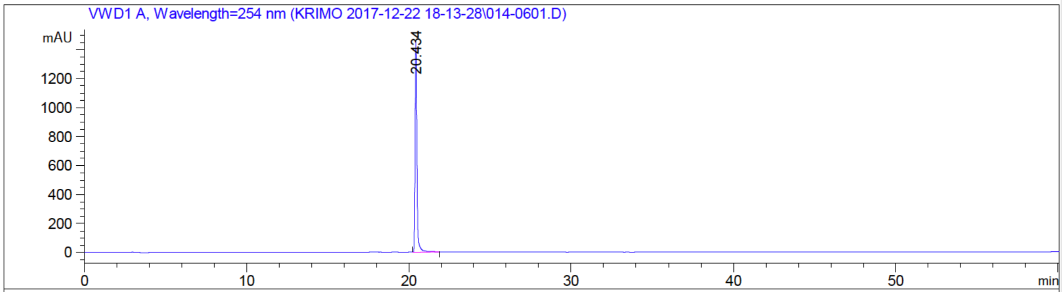
VWD1 A, Wavelength=254 nm (KRIMO 2017-12-22 18-13-28\014-0601.D)
mAU
20.434
min
